# Identifying research priorities in breast cancer surgery: a UK priority setting partnership with the James Lind Alliance

**DOI:** 10.1007/s10549-022-06756-4

**Published:** 2022-11-01

**Authors:** Shelley Potter, Katherine Fairhurst, Katherine Cowan, Simon Vincent, Ian Lewis, Ramsey I. Cutress, Hilary Stobart, Patricia Fairbrother, Sophia Turner, Kayleigh Davies-Crowley, Ranjeet Jeevan, Tim Rattay, Rachel O’Connell, Nigel Bundred, Stuart A. McIntosh

**Affiliations:** 1 Bristol Medical School, Bristol, BS10 5NB UK; 2 Bristol Breast Care Centre, North Bristol NHS Trust, Southmead Hospital, Southmead Road, Bristol, BS10 5NB UK; 3 James Lind Alliance, Southampton, UK; 4 Katherine Cowan Consulting Limited, St Leonards-On-Sea, UK; 5 Breast Cancer Now, London, UK; 6 National Cancer Research Institute, 2 Redman Place, London, E20 1JQ UK; 7 University of Southampton and University Hospital Southampton, Somers Building, Tremona Road, Southampton, SO16 6YD UK; 8 Trustee, Independent Cancer Patients Voice, London, UK; 9 Liverpool University Hospitals NHS Foundation Trust, Liverpool, UK; 10 University Hospitals South Manchester, Manchester, UK; 11 Leicester Cancer Research Centre, Department of Genetics and Genome Biology, Clinical Sciences Building, University of Leicester, Leicester, LE1 7RH UK; 12 Royal Marsden NHS Foundation Trust, Downs Road, Sutton, Surrey, SM2 5PT UK; 13 Division of Cancer Sciences, University of Manchester, Manchester, UK; 14 Centre for Cancer Research, Queen’s University Belfast, 97 Lisburn Road, Belfast, BT9 7AE UK

**Keywords:** Breast cancer surgery, Research priorities, James Lind Alliance, Consensus

## Abstract

**Purpose:**

A James Lind Alliance priority setting partnership was developed to identify research priorities in breast cancer surgery from individuals with lived experience, at high genetic risk of breast cancer, and healthcare professionals (HCPs).

**Methods:**

‘Uncertainties’ were collected using an online survey. Following an evidence check and development of summary questions, an interim survey asked participants to rank their top 10 research priorities from the question list. Top-ranked questions from patient/carer, high-risk and professional groups were carried forward for discussion to a final online prioritisation workshop.

**Results:**

260 participants (101 patients/carers, 156 HCPs) submitted 940 uncertainties via the initial survey. These were analysed thematically into 128 summary questions in six topic areas. Following evidence checking, 59 questions were included in the interim survey which was completed by 572 respondents. Marked differences were seen in questions prioritised by patients/carers, HCPs and women at high-risk. The top eight priorities in patient/carer and professional groups and top two priorities for high-risk women were carried forward to the online workshop at which 22 participants discussed and agreed the final top 10. Key themes included de-escalation of breast and axillary surgery, factors impacting the development/detection of locoregional recurrence and optimal provision of support for informed treatment decision-making.

**Conclusion:**

The top 10 research priorities in breast cancer surgery have been agreed. However, the observed differences in research priorities identified by patients and professional groups were not anticipated. Top priorities from both groups should inform future UK breast cancer surgical research, to ensure that it addresses questions that are important to breast cancer community as a whole.

**Supplementary Information:**

The online version contains supplementary material available at 10.1007/s10549-022-06756-4.

## Background

Breast cancer is the most common malignancy in the UK [1] and the second most common cancer worldwide [2]. Breast cancer affects over 55,000 patients each year in the UK and one in seven women will develop breast cancer at some point in their lives [1]. Earlier diagnosis and improvements in breast cancer treatments have led to dramatic improvements in survival over the last three decades [1, 3] and more than 75% of women diagnosed with breast cancer will now survive more than 10 years following their diagnosis [1]. As most patients will now be long-term survivors, quality as well as quantity of life are both critical considerations following treatment.

Research is central to improving outcomes, but funding is limited, and research questions must be appropriately prioritised to guide best use of resources. An initial breast cancer research gap analysis was published in 2013 and brought together international experts in breast cancer research who identified 10 critical research gaps and translational priorities [4]. Whilst acknowledging that surgery was the primary treatment for most women with breast cancer, little attention was paid to surgery or the potentially pivotal role of surgeons in breast cancer research. Indeed, surgeons are responsible for diagnosis, treatment and follow-up of patients with breast cancer, as well as the management of women at high risk of breast cancer. They are key members of the multidisciplinary team, integral to forming treatment plans to optimise sequencing and integration of loco-regional and systemic therapies. Surgeons also play a vital role in research, both in terms of conducting surgical research studies and in acting as gatekeepers to trials of systemic therapy. Despite this acknowledged key role, only limited funding is directed to surgical research.

To address these issues, the UK Association of Breast Surgery (ABS) established an Academic and Research Committee with the aim of enhancing care and outcomes for patients with breast disease through the promotion and support of research and innovation. As a first step in the process, the group undertook a research gap analysis to identify opportunities and priorities for breast surgical research [5]. Although this process involved patients, it primarily reflected expert opinion. There remained a need to explore, understand and prioritise research questions which are important to individuals with lived experience of breast cancer as well as healthcare professionals (HCPs), to ensure future research addresses issues that are important and meaningful to patients and clinicians alike.

The ABS therefore undertook a James Lind Alliance (JLA) Priority Setting Partnership (PSP) in breast cancer surgery to identify and prioritise unanswered questions that are important to patients, carers and HCPs to inform the future breast cancer surgery research agenda. This is a robust process, with a clearly defined methodology, which is accepted by funders and professional bodies for identifying and prioritising questions for research [6, 7].

## Methods

The Breast Cancer Surgery PSP was conducted in accordance with the process outlined in the JLA Guidebook over a 26 month period between February 2020 and April 2022 [8]. Delays attributable to the Covid-19 pandemic meant that this process took longer than usual for a PSP.

### Steering group and partner organisations

Steering group members were recruited from UK professional and charitable organisations and included patients, breast and plastic surgeons and specialist nurses. Stakeholder representation on the steering group included The ABS (representing breast surgeons and breast care nurse specialists), Independent Cancer Patients’ Voice (ICPV – a patient advocate group independent of UK cancer charities and researchers), Breast Cancer Now (BCN – the UK’s largest breast cancer charity, which provides support for patients and is also a research funder) and the National Cancer Research Institute (NCRI – which connects government departments, charities, industry and research councils to support and develop cancer research in the UK). A JLA advisor (KC) facilitated the process, providing support and guidance to ensure that JLA principles and methods were adhered to throughout. An information specialist team with appropriate qualitative and quantitative methodological expertise (KF, SP) managed the data and undertook the analysis. Each step was overseen by the steering group.

Partners for the PSP were defined as i) people who been treated for breast cancer, ii) those at high-risk of breast cancer requiring surveillance or active risk management, iii) carers or partners of people who have had breast cancer, iv) HCPs including surgeons, breast care nurse specialists and clinicians from other disciplines with clinical experience of the diagnosis and management of breast cancer. As the SARS-CoV-2 pandemic limited the range of PSP activities that were feasible, the steering group focussed on optimising engagement online. This included developing a comprehensive list of partner organisations with whom the PSP could be shared and promoted.

### Scope

The scope of the PSP was defined as including all areas of breast cancer care where breast surgeons were primarily involved in clinical management or where surgical input was central to multi-disciplinary treatment. This included but was not limited to:

- Assessment, diagnosis and primary treatment selection for women and men with invasive and non-invasive breast cancer
- Surgical techniques, technologies and devices, including oncoplastic and reconstructive breast cancer surgery, and their implementation and evaluation
- Interactions between surgical treatments and neoadjuvant/adjuvant systemic and loco-regional therapies
- Quality of life issues related to the surgical treatment of breast cancer
- Identification and management of people at increased risk of breast cancer

Excluded were questions relating to aesthetic breast surgery in individuals without breast cancer; adjuvant breast cancer treatments including chemotherapy, radiotherapy and endocrine therapy; and preclinical or basic science research relating to breast disease.

Decisions about whether questions were in or out of scope were made by the information specialist team and ratified by the steering group.

### Initial survey and identification of themes

The initial survey was developed by the steering group and invited participants to submit research uncertainties in 3 main areas (i) The diagnosis and initial treatment of people with breast cancer, or the care of people at high-risk of developing breast cancer (ii) The choice and timing of breast cancer surgery (iii) Experiences around breast cancer surgery. Examples were included with each question as a prompt to the types of issues that respondents may wish to consider (online Appendix 1). Uncertainties were collected as free text together with simple respondent demographics. Following a successful pilot, the survey was launched in March 2021. The link was disseminated widely to PSP partners through professional groups, charities, patient groups and via social media. The survey was open for a 14-week period between 30/3/21 and 8/7/21. Responses were monitored and efforts made to reach underrepresented groups including men, individuals at the extremes of age and ethnic minorities.

Following the close of the survey, responses were downloaded, cleaned and analysed by the information specialist team. Simple summary statistics were used to summarise respondent demographics. Each free-text response was reviewed. If a response included more than one question, it was broken down into its components so that individual questions could be reviewed and coded separately.

Initially, questions were coded as being in or out of scope (OOS) and OOS questions excluded from further analysis. Questions considered in scope were then reviewed in detail and following a period of data immersion, analysed thematically [9]. Indicative questions were drafted based on emerging themes and iteratively refined as data analysis progressed to ensure they were grounded in the data. Where possible, indicative questions were grouped into summary questions covering a broader topic area.

Batches of indicative and summary questions together with the raw submitted data were reviewed by pairs of steering group members to ensure that the proposed questions captured the essence/meaning of the submitted responses and were comprehensive. Summary questions were revised based on feedback from each small group and the full list of summary questions circulated to the wider steering group for review. The list was iteratively modified based on further feedback from the steering group and the final list of summary questions for evidence checking agreed.

### Evidence checking

The summary questions were checked against evidence to determine which, if any, had already been answered by research and could be excluded from further prioritisation. A high-level evidence check was undertaken focussing on four high-quality clinically relevant data sources to identify the most up-to-date and relevant evidence. The selected data sources were (i) UK guidelines including those from the National Institute of Health and Care Excellence (NICE) and relevant professional surgical associations (ABS] and the British Association of Plastic, Reconstructive and Aesthetic Surgeons [BAPRAS]; (ii) the Cochrane database of systematic reviews, (iii) reviews undertaken by the Early Breast Cancer Clinical Trialists’ Collaborative Group (EBCTCG) and iv) targeted PUBMED searches using key words for each summary question to identify recently published systematic reviews and meta-analyses. Only recent guidelines or reviews published in the last 5 years were considered in the evidence check to ensure they reflected the most up-to-date evidence in each area. Questions were considered answered if recent systematic reviews identified moderate or high-quality evidence to address the topic. The JLA process does not consider ongoing studies when evaluating evidence of uncertainty as it is possible that these studies may not address the uncertainty identified. Ongoing clinical trials were noted but were not included in the assessment of whether or not an uncertainty had been addressed. Questions addressing overlapping issues were merged to generate a smaller number of broader questions to be taken forward to the next stage. Following completion of the evidence check, the final list of answered and unanswered summary questions together with supporting evidence were reviewed and ratified by the steering group. The unanswered questions were then reworded and reviewed by lay members of the steering group to ensure they could be understood by a broad audience and carried forward for prioritisation in the interim priority setting survey.

### Interim prioritisation survey

The interim prioritisation survey asked respondents to choose their top 10 research priorities from the list of summary questions, presented in random order. Simple respondent demographics including respondent group, age, geographical location and ethnicity were also collected and participants asked to express an interest in participating in the final priority setting workshop. The survey was open between 8/2/22 and 21/3/22 and was disseminated widely as previously described.

All questions ranked in the top 10 by respondents were given one point and the total score for each item used to determine the overall rank. Each respondent group was considered separately to promote equal weighting of stakeholder groups. The steering group reviewed the rankings and decided on a manageable number of questions to be carried forward to the final prioritisation workshop.

### Final prioritisation workshop

The final prioritisation workshop was held online on 28th April 2022. Survey respondents expressing an interest were purposively invited to participate based on stakeholder group, geographical location, age and gender to ensure the broadest possible representation of views. Individuals with lived experience were selected to include those who had undergone breast conserving surgery and mastectomy with and without breast reconstruction, neoadjuvant treatment and those individuals at high-risk for breast cancer. An honorarium was offered to lay workshop participants in line with NIHR recommendations.

The consensus process followed standard JLA methodology for online consensus workshops [8]. Prior to the workshop, individuals were sent introductory materials and videos and asked to rank the questions from highest to lowest priority. During the workshop, participants were divided into small groups of 5–6 each including professionals and individuals with lived experience. A JLA advisor facilitated each group and asked each participant in turn to list their highest and lowest priorities and discuss the rationale for their choice. Each small group then discussed and agreed a full ranked order of the questions. The JLA advisors combined the respective rankings from each small group to create a shared rank order that was shared with the wider group. Participants were then allocated to a new small group to review, discuss and revise the shared ranked order of the questions, with the JLA advisor ensuring that all participants had the opportunity to share their reviews before each group re-ranked of the questions. Final rankings from all groups were then combined and to create the final ranked list of consensus priorities that were fed back to the whole group.

### Feedback following presentation of top 10 research priorities

Following the consensus workshop, participants were sent a survey asking for reflections on the process and feedback about the workshop itself.

## Results

**Fig. 1:**
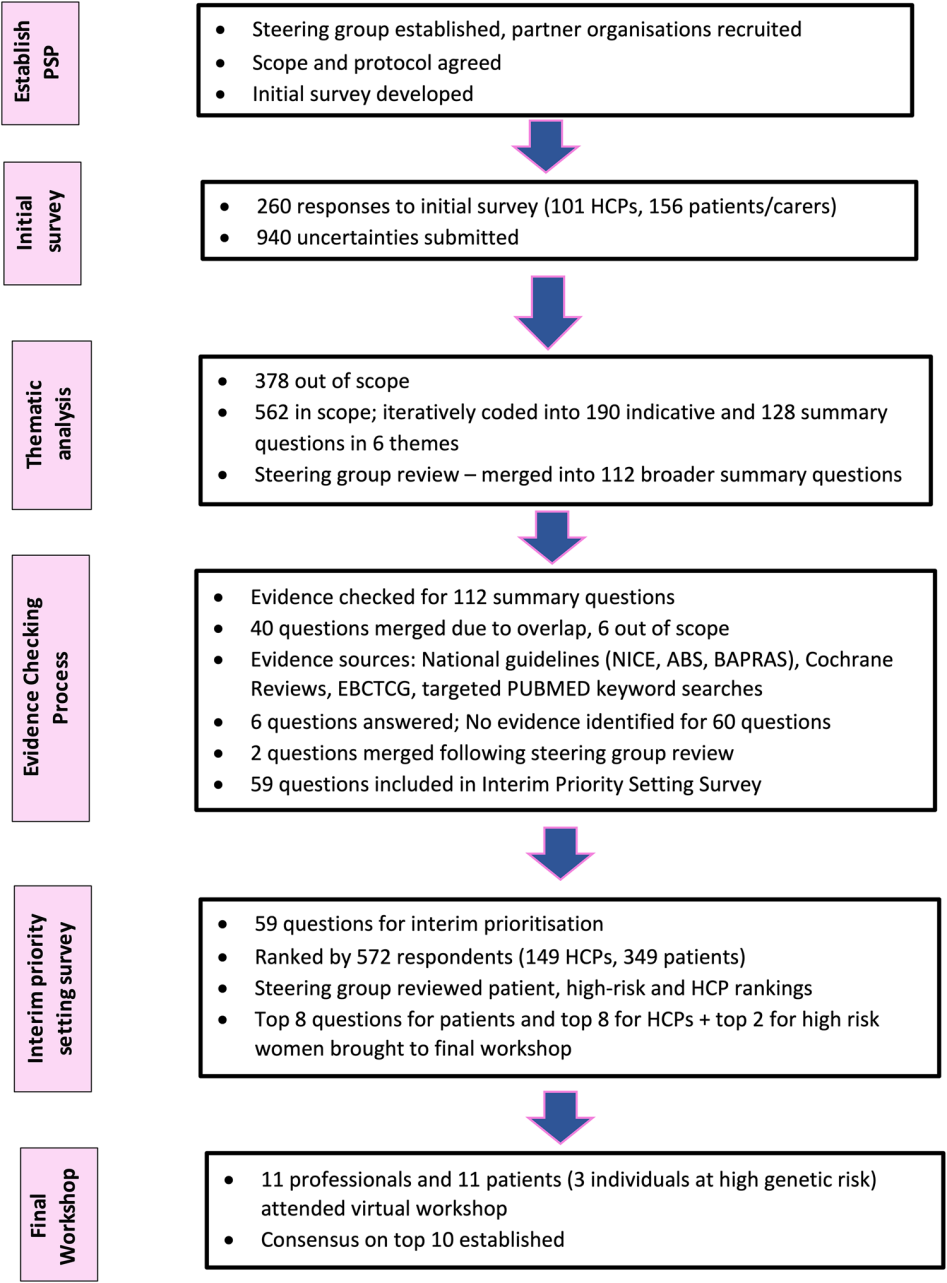
Flow diagram summarising breast cancer surgery priority setting partnership process

This PSP is reported according to REPRISE guidelines [10] and summarised in Fig. 1.

### Initial survey, identification of themes and creation of indicative questions

260 individuals (101 [38.8%] HCPs and 156 [60.0%] patients/carers, 3 [1.1%] unknown) (Table 1) submitted a total of 940 individual questions and/or statements. Of these, 378 (40.2%) were considered OOS and excluded. The remaining 562 questions (289 [51.4%] from HCPs, 219 [39.0%] from patients/carers, 30 [5.3%] from women at high-risk of breast cancer) were grouped in to 190 indicative and 128 summary questions within 6 broad themes reflecting the patient journey and scope of the PSP. There were (i) Breast cancer diagnosis, improving the diagnostic pathway and information and support required at diagnosis; (ii) Treatment sequencing and neoadjuvant treatment; (iii) Breast cancer surgery; (iv) Oncoplastic and reconstructive surgery; (v) Follow-up, detection of recurrence and management of long-term complications of surgery and (vi) issues relating to the management of patients at high-risk of breast cancer (Table 2, full details online Appendix 2).

**Table 1:** Demographics of breast cancer surgery JLA survey participants

	Initial survey *n* = 260 (%)	Interim priority setting survey *n* = 572 (%)
Healthcare professionals	*N* = 101 (39.3)	*N* = 149 (26.1)
Breast surgeon	47	72
Specialist nurses/Advanced nurse practitioners	15	31
Plastic surgeons	3	15
Other/prefer not to say^a^	36	20
Patients/carers/patient representatives	*N* = 156 (60.7)*	*N* = 426 (74.0)
Person being treated/previously treated for breast cancer	146	349
Person at high risk of breast cancer	23	42
Partner/carer of person with cancer/at high risk	17	16
Representatives of organisation representing people with breast cancer	11	16
Age at diagnosis for person treated for breast cancer	*N* = 168	*N* = 349
21–30	1 (0.6)	6 (1.7)
31–40	30 (17.9)	43 (12.3)
41–50	66 (39.3)	116 (33.2)
51–60	47 (28.0)	120 (21.0)
61–70	15 (8.9)	54 (15.5)
71–80	2 (1.2)	10 (2.9)
80 +	0 (0)	0 (0)
Prefer not to say/missing	4 (3.9)	0 (0)
Geographical location		
England	190 (73.1)	321 (56.1)
Scotland	21 (8.1)	22 (3.8)
Northern Ireland	18 (6.9)	47 (8.2)
Wales	9 (3.5)	46 (8.0)
Missing/prefer not to say	4 (1.5)	136 (23.8)
Ethnicity		
White	207 (79.6)	401 (70.1)
Asian	16 (6.2)	19 (3.3)
Black	4 (1.5)	1 (0.1)
Mixed/multiple ethnic groups	5 (1.9)	6 (1.0)
Other/missing/prefer not to say	28 (10.8)	145 (25.3)
Gender		
Female	209 (80.4)	398 (69.6)
Male	31 (11.9)	42 (7.3)
Prefer not to say/missing	20 (7.7)	132 (23.1)
Age of respondents		
21–30	3 (1.2)	5 (0.9)
31–40	22 (8.5)	39 (6.8)
41–50	61 (23.5)	120 (21.0)
51–60	96 (36.9)	166 (29.0)
61–70	46 (17.7)	88 (15.4)
71–80	12 (4.6)	22 (4.9)
80 +	3 (1.2)	1 (0.1)
Prefer not to say/missing	17 (6.5)	131 (22.9)

*Choose one or more responses; ^a^included radiologists/radiographers *n* = 6, oncologists *n* = 3, psychologists *n* = 2

**Table 2:** Numbers of submitted questions, indicative questions and summary questions

Category	Number of submitted questions by respondent type					Number of indicative questions	Number of summary questions
	HCPs (%)	Pts (%)	High risk (%)	Not stated (%)	Total (%)		
Breast cancer diagnosis, improving the diagnostic pathway and information and support required at diagnosis	35 (12.1)	53 (24.2)	11 (36.7)	4 (16.7)	103 (18.3)	22	16
Treatment sequencing and neoadjuvant therapy	29 (10.0)	18 (8.2)	0 (0)	1 (4.2)	48 (8.5)	19	15
Breast cancer surgery	46 (15.9)	39 (17.8)	1 (3.3)	1 (4.2)	87 (15.5)	29	23
Oncoplastic and reconstructive surgery	66 (22.8)	45 (20.5)	4 (13.3)	7 (29.2)	122 (21.7)	54	34
Follow-up, detection of recurrence and management of long-term complications of surgery	79 (27.3)	57 (26.0)	8 (26.7)	10 (41.7)	154 (27.4)	43	22
Management of patients at high risk of breast cancer	34 (11.8)	7 (3.2)	6 (20.0)	1 (4.2)	48 (8.5)	23	18
Total number of questions	289	219	30	24	562	190	128

**Fig. 2:**
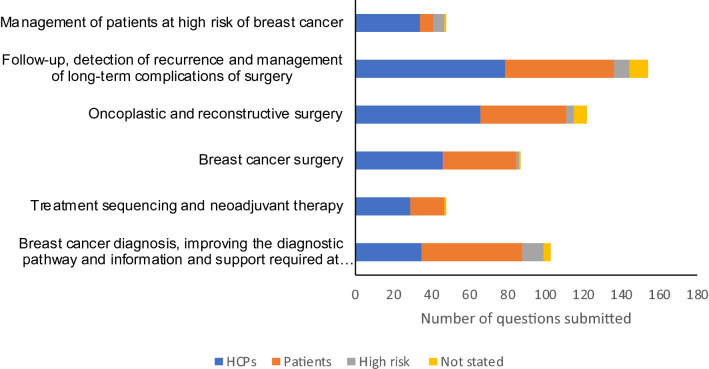
Number of submitted questions by topic and respondent group

More than a quarter of submitted uncertainties (*n* = 154, 27.4%) related to breast cancer follow-up, detection of recurrence and management of long-term complications; over 20% (*n* = 122) related to uncertainties in oncoplastic and reconstructive breast surgery and 18% related to uncertainties around breast cancer diagnosis and the information and support required. Fewer uncertainties related to neoadjuvant therapies/treatment sequencing and management of patients at high-risk patients (Table 2, Fig. 2). Full details of the submitted uncertainties and summary questions can be found in online Appendix 2.

Following review and feedback from the steering group 16 questions were merged to create 112 broader summary questions that were taken forward to the evidence checking stage.

### Evidence checking

During the evidence check, 40 questions were found to overlap and were merged to create broader questions within a similar topic. Details of the evidence identified for each summary question can be found in online Appendix 3. On further review, six questions were considered OOS as they addressed provision/organisation of care and six were excluded as they were considered answered (online Appendix 3). The list of 60 merged unanswered, six answered and six OOS questions were reviewed and ratified by the steering group. Two further questions were merged following steering group discussion and 59 questions were included in the interim prioritisation survey (Supplementary Table 1).

### Interim prioritisation survey

572 individuals completed the interim priority setting survey (Table 1) with 448 (78.3%) respondents (patients/carers n = 342, HCPs n = 106) prioritising at least one question as a top 10 research priority. Marked differences were seen in the research questions prioritised by patients and professionals. Only one question was ranked as a top 10 research priority by both patients/carers and professionals and only seven questions were common to the top 20 priorities in both groups (Supplementary Table 2). Full details of responses by respondent group can be found in online Appendix 4. Patients/carers prioritised questions relating to information provision for decision-making and survivorship issues including outcomes of different types of surgery and detection of recurrence. Professionals prioritised more short-term issues such as the surgical management of screen-detected lesions and the axilla. Furthermore, when the responses of individuals at high-risk as a pre-specified group were considered separately, they too prioritised a different set of questions to both HCPs and the wider patients/carers group (online Appendix 4). As a maximum of 18 questions could practically be discussed at a virtual consensus meeting, a decision was made to take forward the top eight questions from both the patient/carer and HCP groups and the top two questions from the high-risk group to ensure the broadest representation of views (Supplementary Table 3).

### Final consensus workshop

The final meeting was attended by 22 individuals; 11 HCPs (four breast surgeons, four specialist nurses, one plastic surgeon, one physiotherapist and one radiologist) and 11 patients who had undergone a range of surgical treatments including three women at high genetic risk of breast cancer. The final top 10 research priorities are shown in Table 3. Of note, three of the four questions ranked most highly by HCPs in the interim survey, including their top two questions were included in the final top 10 priorities. By contrast, only one of the four questions ranked most highly by patients/carers in the interim surgery were included in the final top 10. Indeed, the question that was the most highly ranked by patients in the interim survey (prioritised by 49.1% of interim survey participants) was not identified as a final top 10 research priority. Despite this, workshop participants reported that they felt the process of reaching consensus was transparent, fair and reflected the views and priorities of the group as a whole.

**Table 3:** Top 10 research priorities in breast cancer surgery

Rank	Question
1	Can complete lymph node removal (axillary clearance) be avoided in patients with spread of cancer to the armpit (axilla); what are the alternatives and the outcomes of this approach?
2	What factors increase the risk of breast cancer returning; Is it possible to predict which patients are at higher risk to help them make a more informed decision about breast cancer surgery?
3	Are minimally invasive, image-guided techniques (e.g. vacuum excision or freezing) to remove or destroy the breast cancer a safe and effective alternative to breast cancer surgery?
4	In patients having breast chemotherapy before surgery, what is the best way of monitoring the cancer and is it possible to tell whether the cancer has completely responded to treatment without performing an operation? How long, if at all, after finishing chemotherapy should an operation be performed?
5	What is the best management of ductal carcinoma in situ (pre-invasive breast cancer) and how is this influenced by tumour and patient characteristics (e.g. patient age, hormone receptor status)?
6	Are there some low-risk breast cancers or lesions detected by breast screening that do not need treatment at all and how it possible to work out which ones these are?
7	How does a breast cancer diagnosis impact on patients’ wellbeing? What information and support do patients want around the time of diagnosis, during and after treatment, and what are the best methods to individualise this?
8	What are the outcomes of mastectomy with and without breast reconstruction; how should these be discussed with patients so that they have realistic expectations of outcomes and can make informed decisions?
9	What is the best method of follow up imaging to detect whether the cancer has returned following breast cancer surgery and how is this influenced by tumour and patient characteristics (e.g. patient age, hormone receptor status)?
10	What is the impact of mastectomy with or without breast reconstruction on quality of life for women at high risk of breast cancer, and when and/or at what age should surgery be performed?

## Discussion

This process has used robust and recognised methodology to identify the top 10 research priorities for breast cancer surgery to inform the future UK research agenda. Key themes in the top 10 include de-escalation of surgery in both the breast (priorities 3, 4, 6) and axilla (priority 1); factors impacting locoregional recurrence and how best to detect this (priorities 2 and 9) and optimal provisional of information and support around diagnosis, treatment decision-making and longer-term outcomes (priorities 7, 8 and 10). Priorities around information and support in breast cancer surgery are consistent with those identified by related PSPs including the NCRI’s Living With and Beyond Cancer PSP [11] and the Canadian breast reconstruction PSP [12]. This reflects the central importance of these questions to patients and professionals and the urgent need for further well-designed research in this area.

Whilst these top 10 questions will be a key priority for future research, a further major finding is the unanticipated dramatic difference in research questions prioritised by patients and professionals in the interim survey. Patients/carers prioritised questions relating to optimising support and informed treatment decision-making whereas professionals generally focussed on clear surgical questions including de-escalation of surgery for low-risk lesions and the axilla. It is notable that only one of the top four questions prioritised by patients during the interim priority setting process was included in the top 10 compared with three out of four top questions in the HCP group. Feedback from the final consensus workshop, however suggested that all participants were satisfied that the final top 10 reflected the views of the wider group. It should be noted that the prioritisation survey is a different process from the final workshop. In the former, participants make decisions alone based on their individual values whereas in the workshop perspectives are shared and the views of the group as a whole used to achieve consensus. Therefore, the final results could legitimately be expected to be different from the interim priorities, although the differences between patient and HCP priorities at this stage of the process remains striking. This is likely to reflect differing priorities directly resulting from an individual’s lived experiences contrasting with the professional experiences of healthcare providers. As such, the value of the insights generated by this work cannot be overstated, as this is the first time that the views of individuals with lived experience of breast cancer have comprehensively been surveyed and prioritised in this way. For this reason, the top 20 patient priorities, in addition to the overall top 10 should be considered an important resource for researchers aiming to undertake work that is important to patients that would meaningfully address the research priorities of this group. The robust process used to generate these priorities should provide reassurance that they are indeed reflective of key uncertainties for the breast cancer community.

There are limitations to this work that warrant consideration. The main challenge was that despite extensive attempts to include and engage partners and partner organisations, engagement from the wider surgical community, especially plastic surgeons was low. Only 79 surgeons submitted uncertainties and only 54 breast and 11 plastic surgeons completed the interim prioritisation survey. In addition, the scope of the PSP was extremely broad and specifically included all aspects of breast cancer care that involved surgeons. Individuals were however conceptually more inclined to focus on and prioritise questions related to breast cancer surgery itself rather than the broader surgical remit and this focus is at least partially reflected in the final top 10 research priorities selected (and may perhaps account for some of the observed differences between patients and clinicians).

The treatment of early breast cancer is by nature multimodality; this PSP focussed on the surgical aspects of the management pathway, and indeed specifically excluded questions related to systemic therapies and radiation treatment except where there was a direct interaction with surgery. A PSP including other disciplines may have generated a different list of research priorities; however, previous gap analyses have addressed unanswered questions around other aspects of the treatment pathway, and this PSP was deliberately intended to identify unanswered questions in breast cancer surgery.

The PSP was undertaken during the Covid-19 pandemic which necessitated the use of exclusively online methods for identifying and prioritising uncertainties. This may have influenced engagement opportunities – for example, it has previously been reported that the use of online methods can result in lower engagement with people from ethnic minority communities [13]. To mitigate this, online surveys were kept open longer than normal, to facilitate completion by those (including clinicians) with many competing commitments. Every effort was made through the PSP partners to engage with men, women at extremes of age and ethnic minorities but this was only partially successful.

Furthermore, the online only approach may have limited the number of questions that could be feasibly discussed at the final consensus workshop. Whilst it is unclear whether it would have impacted the results, discussion of a larger number of questions at an in-person meeting may perhaps have been useful, especially given the divergent views of the stakeholder groups. Similarly, during an in-person workshop, there would have been a final third round of question prioritisation in plenary, which was not possible in the online format. However, by the time of the final workshop, the JLA’s methods for online working were well-developed and the facilitation team experienced in the virtual setting. It is not clear that the pandemic impacted the outcomes of this process, and despite the challenges, the PSP successfully engaged with a large number of individuals with lived experience of breast cancer and provides excellent insight into research areas that are important to patients and therefore worthy of research funding.

Although considered ‘unanswered’ by the JLA, several priorities are the subject of ongoing work both in the UK and internationally. These include the UK NIHR HTA-funded SMALL trial [14] (ISRCTN 12,240,119), comparing open surgery versus minimally invasive vacuum-assisted excision for small screen-detected breast cancer (priority 3); in the setting of neoadjuvant chemotherapy, the UK NOSTRA study (NCT04118192) and European RESPONDER trial (NCT02948764) assessing the ability of imaging and biopsy to identify patients with a complete response to treatment, and the US MD Anderson Exceptional Responders Trial evaluating the omission of surgery in patients with a complete response [https://clinicaltrials.gov/ct2/show/NCT02945579] (priority 4); the European TAXIS trial (https://clinicaltrials.gov/ct2/show/NCT03513614) evaluating de-escalation of axillary surgery [15] (priority 1); the MARECA locoregional recurrence study [16] (priority 2), and the Brighter long-term breast reconstruction outcomes studies [17] (priority 8). Inclusion of these questions in the top 10 priorities for breast cancer surgery research reflects the importance of this work and the need to support these ongoing studies.

Furthermore, the fact that many of the questions are already the subject of ongoing trials in other high-income countries suggests that at least some of the research priorities identified in the JLA process are likely to be shared by the breast cancer community world-wide. However, given the global inequities in breast cancer care that have been highlighted recently [18], it is likely that lower income countries and those with different healthcare systems may have alternative research priorities.

High-quality research is central to improving outcomes for patients with breast cancer and this PSP has set the agenda for future breast cancer surgery research, both by agreeing the top 10 research priorities and identifying previously unanticipated differences in key research priorities for patients and professionals. The next steps will be to translate these priorities into to researchable questions and engage with research funders, patients, clinicians and methodologists to design and deliver well-designed studies that allow these important questions to be addressed.

## Appendices

- Appendix 1: Initial Survey
- Appendix 2: Details of submitted, indicative and summary questions by category
- Appendix 3: Evidence check
- Appendix 4: Interim priority setting survey results by respondent group

## Supplementary Information

Below is the link to the electronic supplementary material.

Supplementary file1 (DOCX 29 KB)

Supplementary file2 (DOCX 27 KB)

Supplementary file3 (DOCX 140 KB)

Supplementary file4 (DOCX 179 KB)

Supplementary file5 (DOCX 30 KB)

## Data Availability

The datasets supporting the conclusions of this article can be obtained by contacting the corresponding authors, or from the James Lind Alliance website (https://www.jla.nihr.ac.uk/priority-setting-partnerships/breast-cancer-surgery/).
