# Supplementary material for: Identifying research priorities in breast cancer surgery: a UK priority setting partnership with the James Lind Alliance
Source: Breast Cancer Res Treat. 2022 Nov 1;197(1):39–49. doi: 10.1007/s10549-022-06756-4 (PMC9628302; doi:10.1007/s10549-022-06756-4)
Supplement: Supplementary file 1 — Supplementary file1 (DOCX 29 KB) [file 10549_2022_6756_MOESM1_ESM.docx]

Breast Cancer Surgery

Priority Setting Partnership

# Why are we doing this?

**We want to make sure that future research in breast cancer surgery is focussed on answering questions that are important to patients, as well as to their families and to healthcare professionals.**

Breast cancer remains one of the commonest cancers in the UK. Everyone diagnosed with breast cancer will see a breast surgeon at some stage, and almost everyone has an operation as part of their treatment. Breast surgeons are responsible for many aspects of the breast cancer patient journey, including:

- Seeing people with new breast symptoms in the outpatient clinic to make a diagnosis
- looking after people who are at high risk of getting breast cancer for example because of a history in the family
- working with other healthcare professionals, including nurse specialists, oncologists, radiologists and pathologists, to deliver the full range of breast cancer treatments
- minimising complications and improving quality of life after breast cancer treatment

# Who should take part?

- Women and men who are being treated for or who have had breast cancer
- People who have been told that they are at high risk of developing a breast cancer
- Friends and family of people who are being treated for or who have been treated for breast cancer
- Health and social care providers (including doctors, nurses, and others) who care for patients with breast cancer

# What’s involved if I take part?

Taking part is voluntary. You don’t have to answer any questions that you don’t want to. By completing the survey, you have given consent for us to analyse your responses and use anonymised responses in our project reports (no individuals or organisations that have taken part will be identifiable).

More information can be found in our Frequently Asked Questions

# Part 1 – what do you feel are the questions about breast cancer surgery that could be answered through research?

We are asking for questions about **all aspects of breast cancer** surgery and the work done by breast surgeons. When you answer please think about your own personal or professional experience with breast cancer, breast cancer surgery and involvement with the breast cancer surgical team. Questions can include physical, mental, emotional or social factors.

Don’t worry about what box to write your question(s) in.

Some of the questions for breast cancer surgical research might seem similar or overlap, so please write your responses in any box that you feel is appropriate.

Write any question that you feel is important, regardless of whether or not you think that someone else may ask them or that they may already have been answered.

What questions for research do you have about:

1. The diagnosis and initial treatment of people with breast cancer, or the care of people at high risk of developing breast cancer?

- Some examples might include identifying the best methods of counselling, support and provision of information at the time of diagnosis, or the choice, order and timeliness of tests and treatments

1. The choice and timing of breast cancer surgery?

- Some examples might include understanding advantages of different types of operations, reconstruction of the breast, and the timing of cancer and reconstruction surgery in relation to other treatments (such as chemotherapy or radiotherapy)

1. Experiences around breast cancer surgery?

- Some examples might include best pain treatments, drain and dressing management, discharge home, follow-up and support, complications, short- and long-term outcomes and side-effects.

# Part 2: about you

We ask for some information about you, so that we can understand who has responded to our questionnaire and make sure that we have heard from all groups.

This will help us keep track of who is completing the questionnaire, and make sure we hear from all groups

1. Which of the following best describes you (tick all that apply)?

Person currently being treated for breast cancer, or who had been treated for breast cancer at any time in the past

Partner/relative/friend of a person with breast cancer (either now or in the past)

Caregiver/former caregiver of a person with breast cancer

Healthcare professional – breast surgeon. plastic surgeon, other medical professional, breast care nurse, research nurse, physiotherapist, other, prefer not to say

Person at high risk of breast cancer (e.g. due to a gene mutation or a strong family history of breast cancer)

Partner/relative/friend of a person at high risk

Part of an organisation representing people with breast cancer

No direct experience of breast cancer but interested in it

Other (please specify)

1. Which part of the UK do you live in? – England/Wales/Scotland/Northern Ireland/prefer not to say
2. Ethnic group (Asian, Black, Mixed/multiple ethnic groups, white, other, prefer not to say)?
3. How do you identify? (male/female/other/prefer not to say)
4. Current age? (by 10 year groups) (≤20, 21-30, 31-40, 41-50, 51-60, 61-70, 71-80, >80, prefer not to say)

Age at diagnosis (if a breast cancer patient or have had breast cancer in the past) (≤20, 21-30, 31-40, 41-50, 51-60, 61-70, 71-80, >80, prefer not to say).

# Part 3: Your contact information

The next part of the project involves putting the research topics identified from this questionnaire into order of importance. This will help to decide which of the research areas identified by the survey are the most important.

If you are interested in taking part in this, please enter your name and email address below (we will only contact you if you provide this). If you don’t give us your contact details, your participation will be anonymous, and we will not contact you again.
