# Supplementary material for: Identifying research priorities in breast cancer surgery: a UK priority setting partnership with the James Lind Alliance
Source: Breast Cancer Res Treat. 2022 Nov 1;197(1):39–49. doi: 10.1007/s10549-022-06756-4 (PMC9628302; doi:10.1007/s10549-022-06756-4)
Supplement: Supplementary file 2 — Supplementary file2 (DOCX 27 KB) [file 10549_2022_6756_MOESM2_ESM.docx]

**Appendix 2: Breast cancer surgery JLA -Details of submitted, indicative and summary questions by category**

***Questions about breast cancer diagnosis; improving the diagnostic pathway and information and support required at diagnosis***

| ***Q no*** | | ***Raw data*** | ***Resp type*** | | ***Indicative question(s)*** | | | ***Summary questions*** |
| --- | --- | --- | --- | --- | --- | --- | --- | --- |
| ***1. How well do different breast cancer symptoms (e.g. breast pain) predict for a breast cancer diagnosis and what is the best way of investigating them?*** | | | | | | | | |
| **A130.2** | | Understanding the significance and diagnostic prediction of different presenting symptoms eg pain v lump v discharge | H | | How well do different breast cancer symptoms predict for a breast cancer diagnosis and how should they be investigated? | | | ***How well do different breast cancer symptoms (e.g breast pain) predict for a breast cancer diagnosis and what is the best way of investigating them?*** |
| **A174.1** | | Absolute clarity on the significance (or not) of pain as a potential breast cancer symptom. If this message can be safely and with clinical data there are huge savings to be made in time for patients, GPs and hospital doctors and decreasing unnecessary worry for all concerned. | H | |  |  |  |  |
| **A130.1** | | Identifying the best method of assessment of those with breast symptoms (which tests, what order, when) to ensure rapid and sustainable care | H | |  |  |  |  |
| ***2. Are there any breast lesions that we can avoid biopsying based on patient or mammographic characteristics?*** | | | | | | | | |
| **A18.3** | | Are there any lesions we can avoid doing biopsies on based on appearance on mammo/patient characterics? | H | | Are there any breast lesions that we can avoid biopsying based on patient or mammographic characteristics? | | | ***Are there any breast lesions that we can avoid biopsying based on patient or mammographic characteristics?*** |
| ***3. How can we improve the experience of men attending breast clinics?*** | | | | | | | | |
| **A129.1** | | Q. What provision could be made to limit the additional stress men attending breast clinics might be feeling, surrounded by a totally pink environment, both visually and in written information, notice boards, waiting rooms, etc. | PR | | How can we improve the experience of men attending breast clinics? | | | ***How can we improve the experience of men attending breast clinics?*** |
| ***4. How much information does a patient want to know before a formal breast cancer diagnosis (i.e. when a highly suspicious lesion is biopsied) and what is the best way to support patients during this period?*** | | | | | | | | |
| **A62.1** | | How much information would the patient want to know at initial visit when highly suspicious lesion identified? | H | | How much information does a patient want to know prediagnosis (i.e when a highly suspicious lesion is biopsied) and how can be best support patients during this period? | | | ***How much information does a patient want to know before a formal breast cancer diagnosis (i.e. when a highly suspicious lesion is biopsied) and what is the best way to support patients during this period?*** |
| **A122.3** | | I still don't think that we have the psychological care around the early stages of diagnosis well mapped out. I am distressed when a woman walks out of a one stop clinic after a biopsy with no opportunity to discuss findings/possible outcomes or an appointment for results. I think that there needs to be much more clear directions about how to support women through this time and resources for ensuring women go through the early steps of diagnosis in a timely fashion. | H | |  |  |  |  |
| **A123.3** | | Should major screening centres have an on-site counsellor? | P | |  |  |  |  |
| ***5. How can we improve the efficiency and accuracy of the diagnostic and pre-operative pathway for patients with breast cancer?*** | | | | | | | | |
| **A7.1** | | What is the best method of assessment of breast cancer | H | | How can the diagnostic process for breast cancer be improved? | | | ***How can we improve the efficiency and accuracy of the diagnostic and pre-operative pathway for patients with breast cancer?*** |
| **A90.7** | | How can biopsy and scanning procedures be improved to Provide a more accurate diagnosis before surgery? | P | |  |  |  |  |
| **A38.2** | | Why use the initial needle biopsy which seems to be so often inconclusive then have to wait another week for further biopsy? | P | |  |  |  |  |
| **A90.8** | | How can biopsy and scanning procedures be improved to Reduce the number of upgraded / downgraded diagnoses after surgery? | P | |  |  |  |  |
| **A166.1** | | Being able to determine the need for actual surgery (i.e. cancer) vs exploratory surgery (unknown, suspected) vs ensuring clear margins during first surgery to avoid a repeat visit to theatre (lumpectomy) | PSR | |  |  |  |  |
| **A12.1** | | Reducing the discomfort of breast biopsies, especially looking at the number of repeat biopsies done and how this can be avoided. | H | | How can we reduce the need for repeat breast biopsies when making a breast cancer diagnosis and how can we make these less painful | | |  |
| **A127.1** | | What are the best methods for identifying the size of the cancer so that the patient / Doctor can make the best choice of surgery (e.g. Mammogram suggested 18mm lump, ultrasound suggested 20mm, actual lump on removal was 12mm). | P | | What is the best way to determine the size of the cancer preoperatively to guide breast cancer surgery? | | |  |
| **A85.2** | | Who needs MRI or other contrast imaging at diagnosis and does the use of contrast imaging improve surgical outcomes? | H | |  | | |  |
| **A161.2** | | What is the optimum time for tests to be carried out and what can be done to speed the process and get timely results? | P | | How can we improve the speed and efficiency of the diagnostic pathway prior to breast cancer surgery | | |  |
| **A171.2** | | How long is a reasonable wait for scan results? | P | |  | | |  |
| **A176.1** | | Order and timeliness of tests | P | |  | | |  |
| A84.1 | | How can the time from biopsy to diagnosis be reduced? The most stressful time is the period of uncertainty, once you know you can start digesting and coping with the news. | P | |  | | |  |
| **A105.2** | | How can we reduce time from diagnosis to treatment ? | H | |  | | |  |
| **A79.1** | | The diagnoses is very fragmented. You are given a cancer diagnosis yet no further Information. Offer type of breast cancer ie hormone positive, triple neg comes at a later stage. Scans are also conducted after the diagnosis and the wait to check for spread is very tough. Why is the wait for results so long after a scan. If results were read quicker and discussed at the MDT quicker or not discussed and the ox is informed as soon as they are available it would be much better. Why can the BCN not discuss results? | H | |  | | |  |
| ***6. Are there some patients with breast cancer who don’t require axillary staging? (e.g. ultrasound and/or biopsy at diagnosis or sentinel node biopsy)*** | | | | | | | | |
| **A96.1** | | Should we be undertaking USS in older women with clinically impalpable nodes. | H | | Should we be undertaking axillary USS in older women with clinically impalpable nodes? | | | ***Are there some patients with breast cancer who don’t require axillary staging? (e.g. ultrasound and/or biopsy at diagnosis or sentinel node biopsy)*** |
| **A119.4** | | The value of US Axilla given that only 50% of cancer detected. Should we be biopsying all cancer axillae regardless of appearance or should we biopsy none and do SLN on all? | H | | Should all women with breast cancer have an axillary core biopsy preoperatively or could we perform sentinel node biopsy on all women to avoid the need for biopsy? | | |  |
| B16.4 | | Next step is further evidence on the need or lack thereof of any axillary surgery. | H | | Can we avoid axillary surgery (sentinel node biopsy) in women with low risk biologically favourable breast cancer? | | |  |
| B19.6 | | De-escalation axillary treatment | H | |  |  |  |  |
| A20.2 | | Omitting SNLB in low risk patients | H | |  |  |  |  |
| B20.9 | | Omitting SNLB in low risk (substituting with core biopsy of node) | H | |  |  |  |  |
| C20.15 | | Omit SNLB in favour of core of relevant node | H | |  |  |  |  |
| B85.7 | | Is there any benefit to surgical staging of the axilla in the era of molecular diagnostics? | H | |  |  |  |  |
| A96.2 | | Should we be doing any axillary surgery in older women with Grade 1 or 2 ER+ breast cancer. | H | |  |  |  |  |
| ***7. What are patients’ experiences of a breast cancer diagnosis, what impact does it have on their well-being and how could this be improved?*** | | | | | | | | |
| **A105.1** | | What is the patient experience of receiving a diagnosis of breast cancer in the UK? Has this been addressed at non teaching centres? | H | | What are patients’ experiences of a breast cancer diagnosis, what impact does it have and how could this be improved? | | | ***What are patients’ experiences of a breast cancer diagnosis, what impact does it have on their wellbeing and how could this be improved?*** |
| **A49.3** | | Do you think the timeline between testing, diagnosis and communication of a diagnosis of cancer is reasonable? How do think the person experiences the waiting time between testing and diagnosis? | P | |  |  |  |  |
| **A66.1** | | Sometimes breast patients are treated very quickly after diagnosis, some a few weeks later - what is the psychological impact of having surgery within a few days or a week of diagnosis in comparison to 2 - 3 weeks later after there has been an opportunity to take in all the information / have counselling , tell the family, do some research etc. Does waiting 2 - 3 weeks prior to surgery have a negative impact on psychological health? | H | |  |  |  |  |
| **A169.1** | | How do you assess the impact of the diagnosis of breast cancer? Do you recognise that sometimes the reaction is delayed...I went into a kind of bubble that didn't let me feel the impact and people thought I was very 'up' about it. | P | |  |  |  |  |
| **A56.1** | | Questions about the mental and emotional impact on patients who are diagnosed or at risk. In particular those of us without partners who don’t have that level of close emotional support. | PSR | |  |  |  |  |
| **A2.1** | | Do you consider the impact of ethnicity on a breast cancer diagnosis ? | P | | How does ethnicity impact a breast cancer diagnosis? | | |  |
| ***8. What information and support do patients want when they are first diagnosed with breast cancer and what is the best way to provide this?*** | | | | | | | | |
| **A24.1** | | How best to give patients information about their surgery to take away from the appointment where they are first told they have breast cancer and would be needing surgery. (eg some sort of booklet with tick boxes and links for further support). | P | | ***What information and support do patients want when they are first diagnosed with breast cancer and what is the best way to provide this?*** | | | ***What information and support do patients want when they are first diagnosed with breast cancer and what is the best way to provide this?*** |
| **A55.1** | | What information do patients want at diagnosis and how should this be provided e.g leaflets or talking and opportunity to ask questions later | P | |  |  |  |  |
| **A110.5** | | What is the best information to provide at the time of diagnosis? Consistency around the format of what & how information is given to patients is essential. I have never received any information in written format along my cancer journey. A written individual treatment plan/log would have been extremely helpful. |  | |  |  |  |  |
| **A110.6** | | How can we best support people at the time of their breast cancer diagnosis? This could be included in a written treatment plan. as suggested above. |  | |  |  |  |  |
| **A30.4** | | Who, when, how to best support the patient with a breast cancer diagnosis - rehabilitation, post-habilitation. | PO | |  |  |  |  |
| **A49.1** | | How can you best prepare a person for the news that they have cancer? Are you giving clear and precise information to the person? Do you think that they are able to understand what you are telling them? When a person goes for a mammogram how can you prepare them for the possibility that they might need further tests and explain what those involve. Do you think the timeline between testing, diagnosis and communication of a diagnosis of cancer is reasonable? How do think the person experiences the waiting time between testing and diagnosis? You are making a treatment plan, do you think your patient should be involved in the planning? | P | |  |  |  |  |
| **A179.1** | | There is a lot of information to process when someone receives a diagnosis. How can we ensure patients and carers fully understand the pathway and timeline of the treatment we've agreed on? | PO | |  |  |  |  |
| **A142.1** | | How to best support people at the time of their breast cancer diagnosis? How helpful would written literature be at the time of diagnosis. (Patients have so many questions, but often unable to vocalise these at the time of diagnosis - many of these questions will be similar for all) | P | |  |  |  |  |
| **A170.5** | | What is the best information to provide at the time of diagnosis? | P | |  |  |  |  |
| **A172.1** | | copies of my own results sent to me and access to a person to discuss this with before speaking to the surgeon choice of tests - what is essential? e.g. understand the role of MRI in diagnosis and surgical planning | H | |  |  |  |  |
| **A158.1** | | What support can I get? A more less analytical complex explanation of my diagnosis |  | |  |  |  |  |
| **A74.2** | | I think that once pathology has been confirmed and your 'type' of cancer is known, this info should be given to you in a written format and I think that more detail would be useful for the patient. It shouldn't take a patient asking many months or even a year down the line for more info on their diagnosis. Hormone status, Her2 status, ductal, lobular etc...all this info should be available and even the scores. For example, if a patients has an 8/8 estrogen score this may increase understanding of risk and later drug adherence to anti- estrogen meds. I think that some degree of uniformity of information delivery would benefit patients. I feel that sometimes patients are at the mercy of a consultant or doctor's personality, perspective on risk, experience and manner in which they can relay info. Some are very statistic driven, some can drip information while they assess how much they think you should know, some can make a surgery decision with very little consultation of the patient's wishes or fail to explain why a particular surgery decision has been made and what this means for your chance of recurrence or risk of MBC. I think that this can be very problematic and can lead to major emotional strain for the patient. | P | |  |  |  |  |
| **A78.2** | | *it is information that is required but as and when it is relevant for each step and as I can take it in. Good, clear information and contact to ask further questions. Not bombarded with leaflets * what tests and scans are needed and WHY * surgery type and why that recommendation | P | |  |  |  |  |
| **A124.1** | | Finding more support on a one to one basis at the time of diagnosis, with gentle explanation of what lies ahead of treatment. | P | |  |  |  |  |
| **A161.1** | | What kind of emotional support should be given at time of diagnosis? What is the optimum time for tests to be carried out and what can be done to speed the process and get timely results? How should the order of treatment be prioritised ie surgery before chemo, or the other way round | P | |  |  |  |  |
| **B137.2** | | Was lucky to have lumpectomy four weeks after biopsy etc. But you don’t have time bro take in what’s been said - it’s all a blur and you don’t want ask questions in case it’s already been covered. It was done over the phone so felt very isolated. It would be nice when you called back you didn’t feel like a pest. I think more time should be given to take it all in. | P | |  |  |  |  |
| **A75.2** | | What effect does the ANP have on the breast patient and breast unit as whole? | H | |  |  |  |  |
| **A84.2** | | How much support from CNSs is required at the different stages of the disease and when do patients need support most? In my experience, the screening centre was full of CNSs, but once diagnosed, it was a struggle to get in touch with anybody for support. | P | |  |  |  |  |
| **B90.12** | | Can patient support be improved with a combined, co-ordinated support programme from a CNS and former patient(s)? | P | |  |  |  |  |
| **B135.2** | | In view of nursing shortages what is the best way to support patients with psychosocial support, emotional needs and information (Assuming that the Key Worker for every patient cannot be sustained/ achieved) | H | |  |  |  |  |
| **A99.1** | | Is it better to offer diagnosis and treatment planning in the same appointment, or separately? - do people find it hard to make decisions when they have just been given a difficult diagnosis? or can they cope better with the diagnosis if they've had a chance to make clear treatment plans? or is it different for different people? | P | |  |  |  |  |
| **A74.5** | | One of the biggest areas of concern relates to fertility for younger patients. There needs to be more of an effort to help women understand the risks to their fertility and what their options are...particularly for those beginning neo-adjuvent chemo as this seems to reduce this as an option. For women under 40, this can have life changing consequences and i think that an awkward 'so did you want to have any more children?' when you have been given the news you have stage 3 cancer and need to start chemo asap is not really adequate. There is not enough support at this very delicate decision making stage. All women under 40 should be referred for a fertility consultation even if it is to understand why fertility preservation is perhaps not an option. | P | |  |  |  |  |
| ***9. What is the best way to support partners, carers and children when a patient is diagnosed with breast cancer?*** | | | | | | | | |
| **A145.1** | | Could there be more support for family members, especially under 18s? Two of my children were 12 and 14 at the time of diagnosis. They now suffer from anxiety and depression which they think relates to my having had cancer. My family has a high incidence of breast cancer on my mum's side, although no genetic link has been traced. I have two daughters who are very concerned about their future in terms of developing breast cancer themselves. I think Girls in this position would benefit from some form of counselling/intervention to make them aware of what it means form them in terms of the timing of future breast screening. Also, they need to be taught about checking their breasts regularly. I have done this, but I think they would benefit more if it was part of a wider support package. | P | | What is the best way to support partners, carers and children when a patient is diagnosed with breast cancer? | | | ***What is the best way to support partners, carers and children when a patient is diagnosed with breast cancer?*** |
| **A112.2** | | What is the best way to support partners/carers and advise them on how to support the patient. | P | |  | | |  |
| **A56.2** | | And questions as to the impact for those not only without partners, but without close family support, perhaps because they don’t have surviving relatives, or children, or family are separated by distance. Even getting to hospital for surgery and treatment can be difficult. Questions need to be asked around the impact on people who have little practical support in their lives as well as limited or no emotional support. Ask if outcomes of treatment differ for people who have limited access to that kind of basic support, which in my experience is always assumed to be in place by the professionals. | PSR | |  | | |  |
| ***10. How can we establish how much information patients want and how much they wish to be involved in decision-making about their breast cancer treatments?*** | | | | | | | | |
| **A125.1** | | I understand that different people want different levels of information, but work could usefully be done on establishing that at the outset for each patient, then identifying sources of information depending on how much the individual wants to know. I spent a lot of time trying to find stuff out for myself (particularly in relation to staging, grading, treatment options and prognosis) because clinicians didn't seem interested in talking about these things. | P | | How do we establish how much information patients want and how much they wish to be involved in decision-making about their breast cancer treatments? | | | ***How can we establish how much information patients want and how much they wish to be involved in decision-making about their breast cancer treatments?*** |
| **A168.1** | | How much information do people want about their possible treatments - all up front or on a ‘need to know’ basis? | P | |  |  |  |  |
| **A168.2** | | How much say do people want in decisions about their treatment plan? | P | |  |  |  |  |
| **A151.1** | | I had bilateral breast cancer Explaining what is about to you and why. Went through a number of procedures before even the surgery but not always sure what exactly was happening to me and why. I simply surrendered to their better judgement | P | |  |  |  |  |
| ***11. What are the most effective ways of providing information to patients with breast cancer?*** | | | | | | | | |
| **B62.2** | | Would video resources talking about the cancer procedure be useful or is written literature more helpful? | H | | What are the most effective ways of providing information to patients with breast cancer? | | | ***What are the most effective ways of providing information to patients with breast cancer?*** |
| **A97.1** | | Best format to give information (i.e. written/website addresses) How much and what type of information to give. | H | |  |  |  |  |
| **A104.1** | | more information on what tools best used for giving information (written/social media/visual) | H | |  |  |  |  |
| **C116.3** | | My after surgery was fine, but felt confined to a booklet. WE get lots of booklets, and they can be overwhelming | P | |  |  |  |  |
| **A148.1** | | How to provide full accurate information Where is the best place for me, with my individual wants and needs, to be treated | P | |  |  |  |  |
| **C161.8** | | How should outcomes be explained prior to op? | P | |  |  |  |  |
| **C77.5** | | There is nothing better than speaking to someone who has been through the same thing. I know everyone's experience is different but speaking to another woman who has had the same surgery is so helpful. Doctors and nurses can only tell you so much. I think having peer support is great, this is something that could be set up? Having a group of volunteers who have previously had surgery, who a patient could call for support and to discuss their feelings and experience. I know that Action Cancer already do this but it only happens after surgery, so giving a patient the option of this from diagnosis would be great. | POR | |  |  |  |  |
| **B98.3** | | How do we give patients the chance to meet with others having surgery when the timeline is so quick? How many patients are offered the chance of a face to face meeting with a patient who has undergone the same type of treatment? | H | |  |  |  |  |
| ***12. What psychological support do patients with breast cancer need; when do they need it and what type of support is most effective?*** | | | | | | | | |
| **A2.2** | | Should psychological support be offered throughout diagnosis and treatment? | P | | What psychological support do patients with breast cancer need; when do they need it and what type of support is most effective? | | | ***What psychological support do patients with breast cancer need; when do they need it and what type of support is most effective?*** |
| **C3.2** | | Does offering psychological support at the point of diagnosis avoid later depression and anxiety problems in survivorship (i.e. can we prevent some of the depression and anxiety seen in survivors through early intervention)? | H | |  |  |  |  |
| **C168.5** | | What psychological support does the cancer patient need - and when do they need it? I am just coming to the end of one year of treatment and I know the psychological impacts will hit soon, but I have no idea where to go to for support. | P | |  |  |  |  |
| **C49.11** | | What psychological support do you think a patient might need post surgery? | P | |  |  |  |  |
| **C3.1** | | How can the psychological issues best be identified and treated? | H | |  |  |  |  |
| **C3.3** | | Which type of psychological support works best? | H | |  |  |  |  |
| **C3.4** | | And which [type of psychological support] is actually implementable? | H | |  |  |  |  |
| *C169.5* | | Increasing availability of psychological support and getting research on the positive impact of psychological services. | P | |  |  |  |  |
| ***13. What are the best methods for counselling patients diagnosed with breast cancer; when should counselling be offered and does it improve long-term outcomes for patients with breast cancer?*** | | | | | | | | |
| **A7.2** | What are the best methods of counselling for people diagnosed with breast cancer? | | | H | | What are the best methods for counselling patients diagnosed with breast cancer; when should this be offered and does it improve long-term outcomes for breast cancer patients? | ***What are the best methods for counselling patients diagnosed with breast cancer; when should counselling be offered and does it improve long-term outcomes for patients with breast cancer?*** | |
| **A110.1** | What are the best methods of counselling for people diagnosed with breast cancer? | | | PH | |  |  |  |
| **A29.1** | Getting the right counselling / support at the right time | | | P | |  |  |  |
| **A74.3** | I think that given waiting times, referrals for counselling should be made at point of diagnosis. Most patients will need some emotional support at some stage and this should be more readily available. | | | P | |  |  |  |
| **A77.3** | Counselling is a great help and should be offered at all stages of the cancer journey. | | | POR | |  |  |  |
| **A80.1** | Support at the consultation phase for counselling referral. | | | R | |  |  |  |
| **A110.2** | Do people actively referred for counselling have better long term outcomes esp mental health? | | |  | |  |  |  |
| **A129.6** | Q. Why is counselling only offered years later not at the start. | | | PR | |  |  |  |
| **A108.1** | how has covid affected our ability to counsel and support patients and will we go back to doing this as we used to? The majority of cases now receive very little in the way of face to face support and there are those who don't feel we should go back to this (for various reasons including money saving) - this is not in the patients best interest. | | | H | |  |  |  |
| ***14. What is the best way to support men with breast cancer?*** | | | | | | | | |
| **B129.10** | Q. Why are men made to feel they can cope, and left with very little follow up or during treatment support. | | | PR | | What is the best way to support men with breast cancer? | ***What is the best way to support men with breast cancer?*** | |
| **C129.16** | Q. Why are men less likely to be consoled, or offered emotional support than ladies. | | | PR | |  |  |  |
| **C129.17** | Q. Why post treatment are men offered no follow up support, even in the way of information re external support groups. | | | PR | |  |  |  |
| **A131.1** | Counselling is a big question particularly for men | | | P | |  |  |  |
| **A133.1** | Support is important As a man who has had breast cancer , the support for me was non existent . I was only offered support with women which was not what was wanted . More separate focusing on MEN | | | P | |  |  |  |
| ***15. What is the role of charities and support groups for patients with breast cancer?*** | | | | | | | | |
| **A2.4** | Should charities such as Macmillan play a role immediately after diagnosis particularly for patients from low income families? | | | P | | What is the role of charities and support groups for patients with breast cancer? | ***What is the role of charities and support groups for patients with breast cancer?*** | |
| **A35.1** | Benefits of signposting patients to groups/charities to create support/information networks early on. | | | P | |  |  |  |
| **A86.1** | How useful are support groups and on - line communities in giving up to date information and support? | | | P | |  |  |  |
| **A86.2** | When is the best time to join in with such {support} groups? | | | P | |  |  |  |
| **C140.12** | support and patient groups which might be a source of comparative information to understand whether the experience is typical or not (and how much to worry) | | | P | |  |  |  |
| **C160.3** | Access to patients who have already had successful surgery should be offered. They know what to expect and can offer practical support and reassurance. | | | P | |  |  |  |
| ***16. How can we improve communication between patients with breast cancer and the clinical team?*** | | | | | | | | |
| **A84.3** | How can communication be improved between patient/carer and clinical team? Would e.g. the use of email be acceptable to clinicians? Would services be more efficient if patients carried their own notes (as well)? I have experienced so many delays due to notes going astray is a source of frustration for patients and clinicians alike. | | | P | | ***How can we improve communication between patients with breast cancer and the clinical team?*** | ***How can we improve communication between patients with breast cancer and the clinical team?*** | |

***Questions about treatment sequencing and neoadjuvant therapy***

| ***Q no*** | ***Raw data*** | ***Resp type*** | ***Indicative question(s)*** | ***Summary questions*** |
| --- | --- | --- | --- | --- |
| ***17. What is the best order of breast cancer treatments (surgery, chemotherapy and radiotherapy) and how can we tailor treatment decisions for individual patients?*** | | | | |
| A7.3 | What is the best order of treatments for people diagnosed with breast cancer? | H | What is the best order of breast cancer treatments (surgery, chemotherapy and radiotherapy) and how can we tailor decisions for individual patients? | ***What is the best order of breast cancer treatments (surgery, chemotherapy and radiotherapy) and how can we tailor treatment decisions for individual patients?*** |
| B110.9 | What is the best order of treatments for people diagnosed with breast cancer? What order gives the best outcomes? |  |  |  |
| A112.3 | Best order of treatments. | P |  |  |
| B130.4 | Understanding the optimum sequencing of breast surgery to adjuvant treatments in different circumstances | H |  |  |
| A140.1 | choice, order and timeliness of tests and treatments | P |  |  |
| A170.1 | What is the best order of treatments for people diagnosed with breast cancer? What are the advantages/disadvantages of the | P |  |  |
| A63.1 | -How to determine (perhaps based on molecular information) which patient groups will most likely to benefit from neoadjuvant treatment before surgery. Specifically those patients who are ER+HER2- node positive as they may not respond optimally to chemotherapy. | H | Can molecular testing be used to determine who would benefit from neoadjuvant treatment (and with what) |  |
| C12.4 | Enhancing neoadjuvant endocrine therapy and predictive tests to look at those likely to most benefit from this rather than upfront surgery | H |  |  |
| A20.7 | Up front genomic testing to guide management | H |  |  |
| **A90.9** | Does knowing Ki-67 at diagnosis change the treatment options and/or treatment success e.g. neo-adjuvant vs adjuvant chemotherapy? | P |  |  |
| ***18. How can we decide which patients with breast cancer would benefit from neoadjuvant chemotherapy or neoadjuvant endocrine therapy before surgery?*** | | | | |
| B85.4 | Which patients with ER+ disease benefit from pre-surgical treatment, and which should have neoadjuvant chemotherapy vs endocrine therapy? | H | How can we best decide which patients with breast cancer would benefit from neoadjuvant chemotherapy or neoadjuvant endocrine therapy before surgery? | ***How can we decide which patients with breast cancer would benefit from neoadjuvant chemotherapy or neoadjuvant endocrine therapy before surgery?*** |
| ***19. Can we predict which patients with breast cancer who need a mastectomy at diagnosis will be able to have breast conserving surgery if they have neoadjuvant chemotherapy (chemotherapy before surgery)?*** | | | | |
| **B127.3** | Would it be possible to have a model (like NHS Predict) into which you could put your breast cancer details and see the percentages of survival / risk of recurrence/ need for further surgery for different options of surgery, timing in relation to other treatments. e.g. If you are offered chemotherapy first to shrink the lump what are the chances that i would lead to a lumpectomy rather than a mastectomy? | P | Possible to predict when down-staged from mastectomy to wide local excision | ***Can we predict which patients with breast cancer who need a mastectomy at diagnosis will be able to have breast conserving surgery if they have neoadjuvant chemotherapy (chemotherapy before surgery)*** |
| ***20. How can we best support patients with breast cancer having neoadjuvant chemotherapy?*** | | | | |
| **A74.6** | For patients starting neo-adjuvant chemo, they tend to move to oncology and therefore do not have access to a breast care nurse which seems to fall under the surgery side of things which i think can be very isolating for the patient as they aren't able to build up a rapport and this can also lead to a delay in referral or signposting to support services. | P | How can we best support patients with breast cancer having neoadjuvant chemotherapy? | ***How can we best support patients with breast cancer having neoadjuvant chemotherapy?*** |
| **A54.1** | For patients undergoing neo adjunctive chemo more support needed for the pre surgical period. | P |  |  |
| ***21. What is the best order of breast cancer treatments (surgery, chemotherapy and radiotherapy) in women having immediate breast reconstruction to minimise complications and improve outcomes?*** | | | | |
| B14.2 | 5 cm cancer er8 pr8 her2 negative with a positive biopsy of lymphnode in axilla Is it not better to do chemotherapy first so any minimal complication of breast reconstruction is not causing any delay in the systemic treatment? | H | Is giving chemotherapy before surgery a better option for women opting for immediate breast reconstruction to reduce complications and avoid delays to treatment? | ***What is the best order of breast cancer treatments (surgery, chemotherapy and radiotherapy) in women having immediate breast reconstruction to minimise complications and improve outcomes?*** |
| B112.5 | Best order of treatment for successful outcome - Pros and cons to different orders of treatment Best order of treatment for successful reconstruction | P |  |  |
| B170.10 | When reconstruction is due before chemotherapy, should surgeons anticipate weight gain due to steroids/ changes in body shape due to chemotherapy | P |  |  |
| B67.5 | Radiotherapy prior to breast reconstruction after chemotherapy. Does this lead to an increase in complications or a reduction in long-term complications? | H | What is the role of neoadjuvant radiotherapy in women having immediate breast reconstruction and how does it impact short and long term complications? |  |
| B109.2 | I would like to know more about changing the order of treatments given. e.g. Undertaking mastectomy and breast reconstruction immediately after neo-adjuvant radiotherapy? | H |  |  |
| ***22. Which patients with breast cancer should have neoadjuvant therapy before surgery and what are the benefits of this approach (e.g. does it improve breast cancer survival)?*** | | | | |
| A178.4 | What is the role of neoadjuvant chemotherapy, radiotherapy and hormone therapy? | H | What is the role of neoadjuvant treatment | ***Which patients with breast cancer should have neoadjuvant therapy before surgery and what are the benefits of this approach (e.g. does it improve breast cancer survival)?*** |
| B20.10 | Neo adjuvant treatment beneficial or not | H | Does neoadjuvant therapy improve outcomes (e.g. survival) for women with breast cancer |  |
| B22.5 | Does neoadjuvant chemotherapy with new agents improve length of quality of life of patients (compared with adjuvant chemotherapy) - the RCT evidence has already shown that NACT with older drugs does not improve survival at all and could be detrimental to survival - it has also shown a worse local control with NACT. - the question is whether continual use of neoadjuvant chemotherapy is justified except in patients in whom initial surgery is just not possible without NACT. | H |  |  |
| B78.4 | *theatre time / recovery time / possible long term consequences of your decision / what does the timing mean in relation to my survival - what does the evidence show - should I have surgery before chemo / after chemo - being alive is the main concern at this point | P |  |  |
| B7.4 | When should chemotherapy be given before breast cancer surgery? | H | Which patients should have neoadjuvant chemotherapy before surgery and what are the benefits of this approach? |  |
| B79.2 | Why do some people have chemo before surgery and some after. | H |  |  |
| A123.4 | Which to do first: chemo or surgery; does this choice affect outcome? | P |  |  |
| B43.3 | Do they do chemo first, if so why that way? | H |  |  |
| A161.3 | How should the order of treatment be prioritised ie surgery before chemo, or the other way round | P |  |  |
| A167.1 | The choice of whether to go straight to surgery or have chemo and/or RTh first | PSO |  |  |
| B170.8 | When should chemotherapy be given before breast cancer surgery? | P |  |  |
| B118.2 | Yes agree discussion & explanation about when surgery will happen in relation to chemotherapy & why. | P |  |  |
| B144.2 | What is the advantage to having surgery before or after chemotherapy? | P |  |  |
| ***23. Can we use short-course treatment before surgery to predict the long-term outcomes of breast cancer and identify patients at high risk of breast cancer recurrence?*** | | | | |
| A177.4 | In light of POETIC data (and EPHOS-B) should all patients have preop AI /endocrine or antiHER2 therapy before surgery routinely? | H | Can we use short-course treatment before surgery to predict the long term outcomes of breast cancer and identify patients at high risk of breast cancer recurrence? | ***Can we use short-course treatment before surgery to predict the long-term outcomes of breast cancer and identify patients at high risk of breast cancer recurrence?*** |
| ***24. If hormone receptors are not assessed prior to neoadjuvant chemotherapy, does it alter the lifespan of the patient?*** | | | | |
| **B121.1** | If hormone receptors are not assessed prior to neoadjuvant chemo, does it alter the lifespan of the patient? | H |  | ***If hormone receptors are not assessed prior to neoadjuvant chemotherapy, does it alter the lifespan of the patient?*** |
| ***25. What is the optimal duration of treatment with neoadjuvant endocrine therapy and when should surgery be performed in patients with breast cancer?*** | | | | |
| B68.2 | Timing of surgery after neoadjuvant endocrine therapy | H | Duration of NET and timing of surgery | ***What is the optimal duration of treatment with neoadjuvant endocrine therapy and when should surgery be performed in patients with breast cancer?*** |
| B71.2 | Neoadjuvant endocrine therapy is under used and is an area needing greater research - for example what is the time to maximum endocrine response. What genetic signatures determine endocrine response. | H |  |  |
| ***26. Should radiotherapy be given before breast cancer surgery and in which patients?*** | | | | |
| B170.9 | Should radiotherapy be given before breast cancer surgery? | P | Should radiotherapy be given before breast cancer surgery in which patients? | ***Should radiotherapy be given before breast cancer surgery and in which patients?*** |
| **B12.3** | Research into the role of neoadjuvant radiotherapy and whether that can improve local control in advanced cancers. | H |  |  |
| ***27. What is the most effective way to mark and localise breast cancers and axillary nodes in women having neoadjuvant chemotherapy to avoid the need for further localisation prior to breast cancer surgery?*** | | | | |
| A12.2 | Assessment of different ways of marking biopsy sites to find a marker that is able to stay in the patient throughout a course of neoadjuvant therapy, that can be localised at surgery without further procedures such as guidewires, that is appropriate for marking lymph nodes as well as breast lesions | H | What is the most effective way to mark and localise breast cancers and axillary nodes in women having neoadjuvant chemotherapy to avoid the need for further localisation prior to breast cancer surgery? | ***What is the most effective way to mark and localise breast cancers and axillary nodes in women having neoadjuvant chemotherapy to avoid the need for further localisation prior to breast cancer surgery?*** |
| ***28. Can we assess whether a patient with breast cancer has had a complete pathological response to neoadjuvant chemotherapy without performing surgery?*** | | | | |
| A101.3 | Post neoadjuvant non-surgical assessment for complete pathological response. | H | Can we assess whether a patient has had a complete pathological response to neoadjuvant chemotherapy without performing surgery? | ***Can we assess whether a patient with breast cancer has had a complete pathological response to neoadjuvant chemotherapy without performing surgery?*** |
| ***29. Is surgery necessary if a patient has a complete pathological response to neoadjuvant chemotherapy?*** | | | | |
| B46.4 | Redefine the role of surgery for exceptional responders after NACT | H | Is surgery is required if a patient has a complete pathological response to neoadjuvant chemotherapy? | ***Is surgery necessary if a patient has a complete pathological response to neoadjuvant chemotherapy***? |
| B50.1 | Surgery after complete radiological response after neoadjuvant chemotherapy- do we need to remove original footprint ? | H |  |  |
| B67.6 | Excising the footprint after significant response to neoadjuvant chemotherapy. Is it necessary? | H |  |  |
| ***30. How long after finishing neoadjuvant chemotherapy should breast surgery be performed?*** | | | | |
| B70.4 | How long after systemic therapy should we aim to operate on a tumour after neo-adjuvant therapy | H | How long after finishing neoadjuvant chemotherapy should breast surgery be performed? | ***How long after finishing neoadjuvant chemotherapy should breast surgery be performed?*** |
| B81.2 | Monitoring response to neoadjuvant chemotherapy and the timing to breast surgery. | H |  |  |
| B94.3 | When to move to surgery when neoadjuvant chemotherapy is not tolerated. | P |  |  |
| ***31. What is the best management of the axilla in women who have a good response to neoadjuvant chemotherapy?*** | | | | |
| C12.4 | Further work on the management of the axilla in patients undergoing neoadjuvant therapy. | H | What is the best management of the axilla in women who have a good response to neoadjuvant chemotherapy? | ***What is the best management of the axilla in women who have a good response to neoadjuvant chemotherapy?*** |
| C103.9 | De-escalation of axillary surgery in good responders to neoadjuvant therapy - data on risk of loco regional recurrence, risk of radiation-related complications | H |  |  |

***Questions about breast cancer surgery***

| ***Item no*** | ***Raw data*** | ***Resp type*** | ***Indicative question(s)*** | ***Summary question(s)*** |
| --- | --- | --- | --- | --- |
| ***32. How can we ensure that patients are offered a fully informed choice about their breast cancer treatment options and have sufficient time to make their decisions?*** | | | | |
| **A21.1** | Information on the choice of tests and treatments is critical. I missed being able to have an Oncotype DX test as my consultant never mentioned it. | P | How can we ensure that women are offered a fully informed choice about their breast cancer treatment options and have sufficient time to make their decisions? | ***How can we ensure that patients are offered a fully informed choice about their breast cancer treatment options and have sufficient time to make their decisions?*** |
| **B176.2** | Patient choice, is it working in practice? | P |  |  |
| **A31.1** | While patients are usually given respect and freedom of choice of treatment at their initial diagnosis, it is unusual for them to be given the freedom of choice at subsequent stages of treatment. But they should be. This would entail an increased amount of effort. For example, if the sentinel lymph nodes are unexpectedly found to be involved, are the patients allowed a meaningful choice on further surgery, its extent, the sequelae. After surgery and the results, are patients given complete information on the choices of further treatment, whether RT or hormone treatment, together with an assessment of risks and benefits. They should be. Though it is time consuming. | H |  |  |
| **B74.7** | I think that patients should be given information about their surgery decision in relation to the surgery options that are available. At diagnosis, patients can assume that they will automatically have a mastectomy from their knowledge of breast cancer in the media. I think that each surgery option should be explained and why a particular surgery is best for you. I think that patients need to understand better why certain surgery decisions are being made. I think that information about risk also needs to be made clear to patients when discussing surgery decisions. | P |  |  |
| **A135.1** | How do we best communicate choices around breast surgery in a timely way so that people feel that they have made the best choice for them without delaying surgery e..g neoadjuvant chemotherapy or mastectomy vs breast conservation or delayed vs immediate reconstruction | H |  |  |
| **A178.3** | How is information about breast conserving surgery v mastectomy conveyed? | H |  |  |
| **A98.1** | research asking the question - have we really listened to the patient in the choices and timing of what is planned in terms of treatment? | H |  |  |
| **B175.2** | As above - plenty of time to choose option. Impartiality of surgeons. The patient needs the best option for her, not the surgeons favoured procedure. Need good unbiased information. | P |  |  |
| **B141.2** | I asked my surgeon for a mastectomy and was told that it wasn't necessary and I had a wide local excision. I know that from pathology of the tissue removed there wasn't a clear margin. I understand a surgeon will try to preserve as much of the breast tissue as possible but should the patient have more of a say in how much tissue is removed (I would have been happier to have more removed). | P |  |  |
| **B160.2** | Patients views should be foremost in everyone’s minds. Being asked to make life changing decisions at a distressing and frightening time needs support. | P |  |  |
| **A175.1** | Allow time for decision making. Do not bombard patients with everything at once. Listen to the patient. | P |  |  |
| **A143.1** | An understanding of the timelines, how long can we take with our decision making so that we are able to consider all options. | PR |  |  |
| **B152.2** | I had great care and explanations were good. However, the time to make decisions is relatively short. | P |  |  |
| **A128.3** | Should the patient be the one to decide when they are satisfied that all avenues have been explored thoroughly? | H |  |  |
| **B140.10** | timing of making decisions bearing in mind that chemo can have side effects which may be unhelpful in making considered decisions | P |  |  |
| **A138.1** | Options? I was only given an option of CHEMO first then surgery because I’d read elsewhere this was possible. Ensure patients can if want have options? | P |  |  |
| **B106.3** | Better understanding for patients why systemic therapies might be suggested before surgery | H |  |  |
| **B169.2** | Is there enough patient choice. I think that some people feel they have to get on the conveyor belt of treatment and don't question it. They might go through chemo before surgery because that is what they have been told, whereas psychologically they might want surgery first to 'get rid of it'. | P |  |  |
| **B158.2** | Options available. Pro and cons likelihood of recovery any post treatments . | H |  |  |
| **B2.5** | This decision should not be rushed into. What questions are patients asked to determine the best option {choice & timing of breast surgery} for them? | P |  |  |
| **B2.7** | Is information about the pros and cons of each option easily accessed? | P |  |  |
| **B128.4** | Should the patient be allowed to choose when and what is done ? Is there enough information there for them to make truly informed choices without being pressured into making decisions without time to think things through? | H |  |  |
| **A155.1** | I would like to know my choices of surgery and explained in clear, simple and concise conversations. | P |  |  |
| **B158.2** | Options available. Pro and cons likelihood of recovery any post treatments . | H |  |  |
| ***33. What are the short and long-term patient-reported outcomes of simple mastectomy for breast cancer and can these be improved by developing better methods for aesthetic flat closure in women not undergoing breast reconstruction surgery*** | | | | |
| C35.6 | Short term and long term outcomes on social/emotional well-being in relation to all mastectomy outcomes. | P | What are the short and long-term patient reported outcomes of simple mastectomy and how are these related to the appearance of the chest wall after surgery | ***What are the short and long-term patient-reported outcomes of simple mastectomy for breast cancer and can these be improved by developing better methods for aesthetic flat closure in women not undergoing breast reconstruction surgery?*** |
| C100.5 | would be interesting to correlate surgeon assessment of mastectomy scar (they are very variable) with patient reported outcomes for body image / QoL. | H |  |  |
| B49.8 | If a patient expresses a wish to stay flat do you have the knowledge and skills to ensure that the end result is as neat and tidy as possible or should they be referred on the a plastic surgeon post mastectomy as an integrated part of their treatment? | P | Can we develop and evaluate the outcomes of techniques for aesthetic flat closure in women who do not have breast reconstruction following mastectomy? |  |
| A54.2 | More development of aesthetic flat closure for those patients who do not have immediate reconstruction | P |  |  |
| A94.2 | Discussing different scar possibilities if going flat. | P |  |  |
| C162.1 | How will you ensure an aesthetic flat closure for those patients who do not wish to undergo reconstruction? | P |  |  |
| ***34. How can we best support women psychologically after mastectomy for breast cancer?*** | | | | |
| **A113.2** | Emotional support for post mastectomy | P | How can we best support women psychologically after mastectomy? | ***How can we best support women psychologically after mastectomy?*** |
| **A123.5** | Is there a way, or ways, of helping women still feel feminine after a double mastectomy? Do these women benefit from specialist counselling? | P |  |  |
| **C158.5** | recovery psychological support e.g had mastectomy. | H |  |  |
| ***35. What are the outcomes of contralateral risk reducing mastectomy in women with breast cancer but no additional genetic risk and when should it be performed?*** | | | | |
| **A35.2** | Risks/benefits of timing of CPM when requested by patient at same time of single mastectomy (eg at same time or months after a single mastectomy) | P | What are the outcomes of contralateral risk reducing mastectomy in women with breast cancer but no additional genetic risk and when should it be performed? | ***What are the outcomes of contralateral risk reducing mastectomy in women with breast cancer but no additional genetic risk and when should it be performed?*** |
| **B49.9** | Is it reasonable to discuss a double mastectomy if the patient requests that but only one breast has cancer? | P |  |  |
| **B86.4** | Is there an advantage in offering a double mastectomy for patients who have a history of non genetic breast cancer in the family? | P |  |  |
| **B90.14** | Bilateral mastectomy: How many women (with no BRCA gene mutation) Are offered a bilateral mastectomy as a surgical option at diagnosis, alongside all the other options? | P |  |  |
| **B90.15** | b. How many women (with no BRCA gene mutation) ask for a bilateral mastectomy (with or without reconstruction) at diagnosis but are not offered it as an option or are discouraged from it? | P |  |  |
| **B90.16** | How many women (with no BRCA gene mutation) are offered and/or ask to have a bilateral mastectomy and choose to have it? | P |  |  |
| **B90.17** | Of the women who choose to have a bilateral mastectomy, how many are happy with their decision? | P |  |  |
| **B90.18** | Of the women who choose to have a bilateral mastectomy, how many regret their decision (including reasons why)? | P |  |  |
| **A43.2** | Correct surgery to begin eg mastectomy doing a double instead of the effected breast due to further relapse down the line. Why don’t they do double mastectomy’s straight away instead of one breast? | H |  |  |
| **B161.5** | When should mastectomy be considered foe psychological reasons, for example, removing a healthy breast? | P |  |  |
| ***36. How do the oncological (cancer) outcomes (recurrence and survival) of breast conserving surgery and radiotherapy compare with those following mastectomy in patients with breast cancer?*** | | | | |
| A112.4 | Best type of operation to avoid recurrence. | P | What is the risk of recurrence following breast conserving surgery and radiotherapy compared with mastectomy for breast cancer? | ***How do the oncological (cancer) outcomes (recurrence and survival) of breast conserving surgery and radiotherapy compare with those following mastectomy in patients with breast cancer?*** |
| A39.1 | What level of recurrence is there of breast cancer for partial breast removal vs full mastectomy? | P |  |  |
| B86.3 | What is the risk of recurrence following a mastectomy versus a lumpectomy? | P |  |  |
| B85.5 | Is there a survival advantage for breast conserving surgery + RT over mastectomy? | H | Does breast conserving surgery and radiotherapy improve survival when compared with mastectomy for breast cancer |  |
| A101.2 | Does breast conservation surgery improve survival when compared to mastectomy | H |  |  |
| ***37. What are the advantages of disadvantages of breast conserving surgery and radiotherapy vs mastectomy for patients with breast cancer?*** | | | | |
| B79.3 | Pros and cons of lumpectomy and rads vs mastectomy | H | What are the advantages of disadvantages of breast conserving surgery and radiotherapy vs mastectomy | ***What are the advantages of disadvantages of breast conserving surgery and radiotherapy vs mastectomy for patients with breast cancer?*** |
| B170.6 | What are the advantages and disadvantages of different types of operations for breast cancer? | P |  |  |
| ***38. How is decision-making for breast cancer surgery affected if women have had cosmetic breast implants?*** | | | | |
| B18.6 | How do cosmetic breast implants affect surgical decision making | H | How is decision-making for surgery affected in women with breast cancer who have had cosmetic breast implants? | ***How is decision-making for breast cancer surgery affected if women have had cosmetic breast implants?*** |
| ***39. Is minimally invasive, image-guided excision a safe and effective alternative to surgery in women with breast cancer*** | | | | |
| A20.1 | Alternative treatments (less invasive surgery), medications to shrink then less invasive surgery or radiological excision | H | Is radiological excision (vacuum assisted excision) a safe and effective alternative to surgery in women with breast cancer? | ***Is minimally invasive, image-guided excision a safe and effective alternative to surgery in women with breast cancer?*** |
| B20.8 | Use of less invasive surgeries/radiological excision | H |  |  |
| A119.2 | Non-surgical methods of breast cancer treatment: VAE type treatments | H |  |  |
| ***40. Are there some low risk breast cancers or screen detected lesions that we can treat less aggressively or that do not need any treatment at all?*** | | | | |
| **A18.4** | Are there any cancers we can leave alone? | H | Are there some low risk breast cancers or screen detected lesions that do not need any treatment at all? | ***Are there some low risk breast cancers or screen detected lesions that we can treat less aggressively or that do not need any treatment at all?*** |
| **A19.5** | De-escalation treatment of some screen detected lesions | H |  |  |
| **A67.3** | Studies to understand management of risk with in situ and early breast cancer and predictions of longevity. | H |  |  |
| **A85.1** | How do we properly tailor local treatment to level of risk i.e. how can we minimise the surgical overtreatment of low risk disease? | H |  |  |
| **A119.5** | Keep going with LORIS LORDS type trials Psychology of not treating cancer and how to address issues | H |  |  |
| ***41. What is the clinical significance of very small screen detected lesions found on more sensitive imaging (e.g. MRI, tomosynthesis) and do these require surgical treatment?*** | | | | |
| **A130.3** | Understanding the clinical significance of smaller and screen detected lesions found but more sensitive imaging techniques | H | What is the clinical significance of small screen detected lesions found on more sensitive imaging (e.g. MRI, tomosynthesis) and do these require treatment? | ***What is the clinical significance of very small screen detected lesions found on more sensitive imaging (e.g. MRI, tomosynthesis) and do these require surgical treatment?*** |
| **42. What is the best management of ductal carcinoma in situ and how is this influenced by tumour and patient characteristics (e.g. patient age)?** | | | | |
| **B32.4** | Surgical options for DCIS? | H | What is the best management of ductal carcinoma in situ and how is this influenced by tumour and patient characteristics (e.g. patient age)? | ***What is the best management of ductal carcinoma in situ and how is this influenced by tumour and patient characteristics (e.g. patient age)?*** |
| **B100.3** | Management of low and intermediate grade DCIS | H |  |  |
| **B100.4** | management and outcomes of DCIS of any grade in women <50yrs. | H |  |  |
| ***43. Do remote incisions (e.g. incisions around the areola) increase the risk of incomplete excision in women having breast surgery for ductal carcinoma in situ?*** | | | | |
| **B177.8** | Do remote periareolar incisions for DCIS increase reexcision rate and incomplete excision? | H | Do remote incisions (e.g. incisions around the areola) increase the risk of incomplete excision in women having breast surgery for ductal carcinoma in situ? | ***Do remote incisions (e.g. incisions around the areola) increase the risk of incomplete excision in women having breast surgery for ductal carcinoma in situ?*** |
| ***44. How can we better evaluate surgical margins or predict margin involvement at the time of breast conserving surgery to reduce rates of re-excision?*** | | | | |
| B19.7 | Better margin detection so reduction re-operation | H | How can we better evaluate surgical margins at the time of breast conserving surgery to reduce rates of re-excision? | ***How can we better evaluate surgical margins or predict margin involvement at the time of breast conserving surgery to reduce rates of re-excision?*** |
| B41.3 | Can surgical margins be assessed effectively at the time of surgery to avoid re-excisions? | H |  |  |
| B177.6 | How do we ensure surgeons ensure clear margins of cancer excision after breast reconstruction? | H |  |  |
| **C30.7** | Can light techniques help? Raman Spectroscopy? | PO |  |  |
| **B127.3** | Would it be possible to have a model (like NHS Predict) into which you could put your breast cancer details and see the percentages of survival / risk of recurrence/ need for further surgery for different options of surgery, timing in relation to other treatments. e.g. If you are offered chemotherapy first to shrink the lump what are the chances that i would lead to a lumpectomy rather than a mastectomy? | P | Is it possible to predict what patients will have involved margins and require further breast cancer surgery? |  |
| ***45. Can we safely avoid mastectomy in patients with multifocal or multicentric breast cancer?*** | | | | |
| B105.3 | Improving choices for patients with multifocal or mulitcentric cancers to avoid mastectomy? | H | Can we avoid mastectomy in patients with multifocal or multicentric breast cancer? | ***Can we safely avoid mastectomy in patients with multifocal or multicentric breast cancer?*** |
| ***46. What is the best surgical management of rarer breast cancers e.g. metaplastic breast cancer?*** | | | | |
| **A82.1** | More research about how best to surgically approach rare breast cancers such as metaplastic breast cancer. | H | What is the best surgical management of rarer breast cancers e.g. metaplastic breast cancer? | ***What is the best surgical management of rarer breast cancers e.g. metaplastic breast cancer?*** |
| ***47. What are the long-term outcomes for men having breast cancer surgery?*** | | | | |
| C103.11 | Surgery for male breast cancer - long-term outcomes, side effects, support | H | What are the long-term outcomes for men having breast cancer surgery? | ***What are the long-term outcomes for men having breast cancer surgery?*** |
| ***48. How long following diagnosis should surgery for low risk lesions be performed?*** | | | | |
| **B104.3** | identifying the best timing of treatment after diagnosis from the patients perspective for low risk lesions - I believe that 31 day rule is not applicable to all women | H | How long following diagnosis should surgery for low risk lesions be performed? | ***How long following diagnosis should surgery for low risk lesions be performed?*** |
| ***49. Can we avoid axillary clearance in women with node positive breast cancer and what are the oncological and patient-reported outcomes of alternatives to axillary node clearance such as targeted axillary node dissection?*** | | | | |
| C70.6 | Quality of life for TAD vs ANC in N1 disease | H | What are the quality of life benefits for targeted axillary dissection vs axillary clearance for women with node positive breast cancer | ***Can we avoid axillary clearance in women with node positive breast cancer and what are the oncological and patient-reported outcomes of alternatives to axillary node clearance such as targeted axillary node dissection?*** |
| B105.4 | How can we reduce the numbers needing axillary clearance? | H | How can we reduce the numbers of women with node positive breast cancer needing an axillary clearance? |  |
| A119.3 | Nodal surgery treatment benefits in modern era: RDx vs. other therapy | H | What are the benefits of axillary clearance in the modern era? |  |
| ***50. Why has intraoperative radiotherapy (IORT) not been widely adopted in the UK?*** | | | | |
| B22.6 | In patients suitable for partial breast irradiation with intraoperative radiotherapy (TARGIT-IORT) during lumpectomy surgery(which is shown to have comparable breast cancer outcomes and fewer deaths from other causes) what is the reason (conflict of interest and fear of job losses has been suggested as the possible reason) for less than widespread adoption in the UK (whereas it has been widely adopted in other 280 countries including Europe, USA, Middle east, Far East, S Africa, Australia, etc). ? | H | Why has IORT not been widely adopted in the UK? | ***Why has intraoperative radiotherapy (IORT) not been widely adopted in the UK?*** |
| ***51. How soon after diagnosis should surgery for breast cancer be performed and what impact do delays in treatment have on long-term breast cancer outcomes?*** | | | | |
| A170.3 | How soon should treatment/surgery commence? | P | How soon after diagnosis should treatment for breast cancer start? | ***How soon after diagnosis should surgery for breast cancer be performed and what impact do delays in treatment have on long-term breast cancer outcomes?*** |
| B42.1 | How urgent is urgent for lumpectomy for breast cancer. | H |  |  |
| **B27.3** | Are patients explained cancer growth alongside the timeline of treatment? How can we best do this? | H |  |  |
| B89.3 | Assessing impact of delays on prognosis | H | Do delays in breast cancer diagnosis and treatment influence prognosis and if so, how? |  |
| B131.2 | What are the risks in delaying treatment in time bands | P |  |  |
| **A177.1** | Do delays in diagnosis and treatment impact local, distant recurrence and survival . | H |  |  |
| ***52. Are there benefits to performing breast cancer surgery at specific phases in the menstrual cycle for premenopausal women?*** | | | | |
| B65.2 | For women of reproductive age, timing might improve 10 year survival. Fentiman IS: 12. Timing of surgery for breast cancer. Int J Clin Pract. 56:188–190. 2002.PubMed/NCBI (timing of surgery during phases of menstrual cycle) | P | Timing of surgery in relation to phase of menstrual cycle in premenopausal women | ***Are there benefits to performing breast cancer surgery at specific phases in the menstrual cycle for premenopausal women?*** |
| B110.11 | Is there any advantage or impact on outcomes relating timing of surgery or treatment and menstrual cycle? |  |  |  |
| ***53. How long after radiotherapy should further surgery to improve the appearance of the breast be performed?*** | | | | |
| B109.3 | The optimal timing of treatments after radiotherapy in general e.g. lipofilling, revisional surgery. Can we go in early? | H | Timing of further surgery post radiotherapy | ***How long after radiotherapy should further surgery to improve the appearance of the breast be performed?*** |
| ***54. Can molecular tests be used to tailor local therapies for breast cancer?*** | | | | |
| A85.3 | Can we use molecular tests before surgery to tailor local treatment as well as systemic therapy? | H | ??surgery or ??RT not clear – may be OOS | ***Can molecular tests be used to tailor local therapies for breast cancer?*** |

***Questions about oncoplastic and reconstructive surgery***

| **Q no.** | **Raw data from survey** | | | **Resp type** | **Indicative question(s)** | | **Proposed summary question(s)** |
| --- | --- | --- | --- | --- | --- | --- | --- |
| **55. What impact does oncoplastic and reconstructive breast surgery have on breast cancer survival and quality of life?** | | | | | | | |
| B22.1 | We need randomised evidence to show that oncoplastic surgery and reconstructive surgery improves/does not improve length or quality of life of patients | | | H | How does oncoplastic and reconstructive breast surgery impact breast cancer survival and quality of life? | | ***What impact does oncoplastic and reconstructive breast surgery have on breast cancer survival and quality of life?*** |
| B69.2 | Breast reconstruction - evidence and funding for level 1 trials | | | PO |  |  |  |
| B122.4 | Why do we do complex reconstruction? A patient said to me yesterday that her lat dorsi procedure means she can't do yoga, which has had a major impact on her life. She wished that she had had a mastectomy and the money spent on counselling. I sometimes wonder if we encourage reconstruction because it is a challenging operation that tests our skills and is very satisfying when it goes well. Why are we using theatre time on very long operations when we can't manage our surgical waits. | | | H |  |  |  |
| **B22.2** | Do these substantially longer and more invasive procedures [oncoplastic/reconstructive surgery] really improve quality of life of patients | | | H |  |  |  |
| **B22.3** | Does this additional trauma and inflammation around surgery [oncoplastic/reconstructive surgery] for cancer worsen survival? | | | H |  |  |  |
| **56. What are the long-term oncological outcomes (e.g. local recurrence rates) of skin and nipple-sparing mastectomy?** | | | | | | | |
| B103.7 | Long-term oncological outcomes of skin-sparing and nipple-sparing mastectomies (recurrence rates/revisions) | | | H | What are the long-term oncological outcomes of skin and nipple-sparing mastectomy? | | ***What are the long-term oncological outcomes of skin and nipple-sparing mastectomy?*** |
| B91.2 | More research into types of mastectomy and risks / benefits of nipple sparing / skin sparing / simple mastectomies | | | H |  |  |  |
| **B100.2** | up to date figures on local recurrence that relate to the procedures that we now regularly offer - e.g. SSM, x2 WLE in one breast... | | | H |  |  |  |
| **57. What is the best way to identify the mastectomy plane to reduce the risk of local recurrence but maintain blood supply in women having skin/nipple sparing mastectomies for breast cancer?** | | | | | | | |
| B91.3 | How can the thickness of mastectomy skin flaps be best judged for maintain vascularity, whilst removing as much breast tissue was possible | | | H | How can the thickness of mastectomy skin flaps be best judged for maintain vascularity, whilst removing as much breast tissue was possible | | ***What is the best way to identify the mastectomy plane to reduce the risk of local recurrence but maintain blood supply in women having skin/nipple sparing mastectomies for breast cancer?*** |
| C180.3 | Techniques to ID mastectomy plane / completeness of SSMx - ie addressing potential increased recurrence in SSMx | | | H | How can we better identify the mastectomy plane to reduce risk of recurrence | |  |
| **58. Is breast reconstruction safe in women with locally advanced breast cancer?** | | | | | | | |
| B177.5 | In advanced cancers(heavy nodal involvement) is reconstruction safe oncologically? | | | H | Is breast reconstruction safe in women with locally advanced breast cancer? | | ***Is breast reconstruction safe in women with locally advanced breast cancer?*** |
| **59. Does breast reconstruction make it harder to detect breast cancer recurrence?** | | | | | | | |
| **B18.5** | Does reconstruction make it harder to detect recurrence? | | | H | Does breast reconstruction make it harder to detect breast cancer recurrence? | | ***Does breast reconstruction make it harder to detect breast cancer recurrence?*** |
| **60. Is oncoplastic breast conserving surgery oncologically safe in the short and long term and does it influence detection of breast cancer recurrence?** | | | | | | | |
| **B102.1** | extreme oncoplasty for tumours larger than 4 cm local perforator flaps therapeutic mammoplasty outcomes in high BMI patients like 30-35 BMI | | | H | What are the oncological outcomes of oncoplastic breast conservation in patients with large tumours? | | ***Is oncoplastic breast conserving surgery oncologically safe in the short and long term and does it influence detection of breast cancer recurrence?*** |
| **C178.10** | What is the evidence for cancer / tumour excision / clearance rates via breast conserving surgery and therapeutic mammaplasty? | | | H | How do complete tumour excision rates compare in women having oncoplastic vs. standard breast conserving surgery? | |  |
| B22.3 | Does this additional trauma and inflammation around surgery [oncoplastic/reconstructive surgery] for cancer worsen survival? | | | H | Is oncoplastic breast conservation oncologically safe? | |  |
| **B119.10** | With more Oncoplasty, how has recurrence site and appearance changed if at all? | | | H | Do oncoplastic procedures influence the site and appearance of locoregional recurrences? | |  |
| **B97.2** | What statistical evidence there is that 'oncoplastic' procedures actually cause delay in adjuvant therapies. | | | H | Do oncoplastic breast conserving procedures delay the delivery of adjuvant therapies? | |  |
| **B100.2** | up to date figures on local recurrence that relate to the procedures that we now regularly offer - e.g. SSM, x2 WLE in one breast... | | | H | What is the local recurrence rate following multiple lumpectomies (for multifocal/multicentric disease) | |  |
| **61. Is oncoplastic and reconstructive surgery for breast cancer cost-effective** | | | | | | | |
| **B22.4** | Are any of these treatments [oncoplastic/reconstructive surgery] cost-effective? They are not even shown to be oncologically safe, and no benefit has been demonstrated in any RCT yet. | | | H | Is oncoplastic and reconstructive surgery for breast cancer cost-effective? | | ***Is oncoplastic and reconstructive surgery for breast cancer cost-effective?*** |
| **62. Do oncoplastic breast conserving procedures (therapeutic mammaplasty or local perforator flaps) improve the clinical and patient reported outcomes of surgery for women with breast cancer compared with simple wide local excision (lumpectomy) or mastectomy with or without breast reconstruction and in what groups is it particularly beneficial?** | | | | | | | |
| B170.6 | What are the advantages and disadvantages of different types of operations for breast cancer? | | | P | How do the clinical and patient reported outcomes of oncoplastic breast conserving surgery; simple wide local excision and mastectomy with or without immediate breast reconstruction compare? | | ***Do oncoplastic breast conserving procedures (therapeutic mammaplasty or local perforator flaps) improve the clinical and patient reported outcomes of surgery for women with breast cancer compared with simple wide local excision or mastectomy with or without breast reconstruction and in what groups is it particularly beneficial?*** |
| **C70.5** | Quality of life comparing simple wide local vs local perforator flaps vs. therapeutic mammoplasty? | | | H | How does quality of life compare in women undergoing oncoplastic (therapeutic mammaplasty or local perforator flaps) vs standard breast conserving surgery | |  |
| B22.2 | Do these substantially longer and more invasive procedures [oncoplastic/reconstructive surgery] really improve quality of life of patients | | | H | Does oncoplastic breast conserving surgery improve outcomes for women with breast cancer compared with i) standard wide local excision ii) mastectomy | |  |
| B63.2 | Further research would benefit clinical decision making in those patients who present with small cancer in a large ptotic breast. It’s difficult to know whether to offer them standard wide local excision versus therapeutic mammoplasty in order reduce the potential risk of radiotherapy related side effects in this patient group. Fully appreciate that the latter is a higher risk procedure with regards to wound healing risk. It would benefit to gain patient perspectives in this decision making | | | H | Is reduction-type therapeutic mammaplasty a better option than standard wide local excision in women with small cancers in large breasts who will require radiotherapy? | |  |
| **63. What are the clinical and patient-reported outcomes of mastectomy with and without breast reconstruction and how should these be best discussed with patients to help them make an informed decision?** | | | | | | | |
| B49.6 | What are the advantages of reconstruction over staying flat? | | | P | How do the clinical and patient-reported outcomes of mastectomy and breast reconstruction compare? | | ***What are the clinical and patient-reported outcomes of mastectomy with and without breast reconstruction and how should these be best discussed with patients to help them make an informed decision?*** |
| B13.2 | What comparative studies have been published with regard to outcomes and complications of reconstruction or flat closure? | | | P |  |  |  |
| **C13.6** | What are the comparative long-term satisfaction statistics, complication data and quality of life analysis of patients offered aesthetic flat closure and/or contralateral prophylactic mastectomy compared to reconstruction? | | | P |  |  |  |
| **A39.3** | What are the outcomes in terms of satisfaction with treatment for women undergoing reconstruction Vs women choosing to go flat? | | | P |  |  |  |
| **64. Is simple mastectomy presented as an equally valid alternative to breast reconstruction in women with breast cancer and if not, why is this and how can this be improved?** | | | | | | | |
| **B29.3** | Understanding why many surgeons are not happy to allow or do not understand why many women would want to live flat after breast surgery (ie no reconstruction) | | | P | Is simple mastectomy presented as an equally valid alternative to breast reconstruction in women with breast cancer and if not, why is this and how can this be improved? | | ***Is simple mastectomy presented as an equally valid alternative to breast reconstruction in women with breast cancer and if not, why is this and how can this be improved?*** |
| **A39.3** | If staying flat post-mastectomy is presented as a valid choice with equal validity to reconstruction, what effect does this have on the proportion of women choosing to have/forego reconstruction? | | | P |  |  |  |
| **B47.1** | Are you doing the right thing by discouraging/not supporting women who request a bilateral mastectomy for symmetry? Could/should bilateral mastectomy for symmetry (immediate/delayed) be offered as an option to women alongside reconstruction? | | | H |  |  |  |
| **65. What is the psychological impact of not being able to have breast reconstruction in women with breast cancer who would like reconstructive surgery and how can these women be best supported?** | | | | | | | |
| **B82.3** | Research into the psychological effect of those women who cannot have breast reconstruction. | | | H | What is the psychological impact of not being able to have breast reconstruction in women with breast cancer who would like reconstructive surgery? | | ***What is the psychological impact of not being able to have breast reconstruction in women with breast cancer who would like reconstructive surgery and how can these women be best supported?*** |
| **66. What are the clinical and patient-reported outcomes of immediate and delayed breast reconstruction?** | | | | | | | |
| B56.3 | Whether immediate or delayed reconstruction is better in each individual circumstance. | | | PSR | When should immediate vs delayed breast reconstruction be recommended? | | ***What are the clinical and patient-reported outcomes of immediate and delayed breast reconstruction?*** |
| B168.4 | What factors need to be taken into consideration, to give the patient a full understanding of the impact of having a delayed reconstruction versus immediate reconstruction. What are all the surgical options and what are the pros and cons of each? Is surgery required immediately? | | | P |  | |  |
| **B66.3** | Does delayed reconstructive surgery produce a better aesthetic outcome? | | | H | How do the outcomes of immediate and delayed breast reconstruction compare? | |  |
| **67. In what circumstances should immediate or delayed breast reconstruction be offered; what is the psychological impact of not having immediate breast reconstruction and how long after mastectomy should delayed reconstruction be performed?** | | | | | | | |
| B32.3 | When should post mastectomy breast reconstruction be planned? | | | H | When should delayed breast reconstruction be planned? | | ***In what circumstances should immediate or delayed breast reconstruction be offered; what is the psychological impact of not having immediate breast reconstruction and how long after mastectomy should delayed reconstruction be performed?*** |
| B114.2 | Is the choice of delaying reconstruction and the effect on recovery being investigated? | | | P |  |  |  |
| A167.2 | To have an immediate reconstruction seems rash because patients are still coming to terms with the whole issue | | | PSO | Is immediate reconstruction the best option for women having mastectomy for breast cancer? | |  |
| B161.4 | Is the approach around surgery correct? Ie should it be considered that reconstruction is the best option? | | | P |  |  |  |
| **B66.2** | Does the timing of reconstructive surgery influence how well someone deals with the psychological impact of having a mastectomy? | | | H | How does the timing of breast reconstruction influence how patients deal with the psychological impact of having a mastectomy for breast cancer? | |  |
| **68. What are the short and long-term clinical and patient-reported outcomes and cost-effectiveness of different types of breast reconstruction surgery (e.g. implant and tissue-based procedures)?** | | | | | | | |
| B120.4 | What is the success rate of breast reconstruction done at the same time as the breast surgery? | | | P | What are the short-term clinical and patient-reported outcomes of immediate breast reconstruction surgery? | | ***What are the short and long-term clinical and patient-reported outcomes and cost-effectiveness of different types of breast reconstruction surgery (e.g. implant and tissue-based procedures)*** |
| B153.3 | Do people with reconstruction at time of / shortly after mastectomy have better mental health outcomes | | | P |  |  |  |
| B79.4 | Long term issues associated with implant vs autologous reconstruction | | | H | What are the long-term clinical, patient reported and health economic outcomes of implant-based and autologous (tissue-based) breast reconstruction procedures?  (What is the best type of breast reconstruction?) | |  |
| C85.11 | What are the long-term complication rates, quality of life and health economic outcomes of implant-based reconstruction? | | | H |  |  |  |
| B110.10 | I What are the long term complications and impact on general health & quality of life post reconstruction? - | | |  |  |  |  |
| B110.12 | What is the best type of breast reconstruction to have for longevity? | | |  |  |  |  |
| B119.9 | What happened to the women we put implants in? Would they have that operation 12 years later? Are autologous vs. implant women very different in outlook now? Should we put in fewer implants as Autologous really is much better longterm? | | | H |  |  |  |
| B153.2 | Understanding the longer term effects of the surgery options - what will result in longer term success (ie not need replacing again if reconstructed) | | | P |  |  |  |
| B112.7 | Best treatment for reconstruction | | | P |  |  |  |
| A117.2 | Is one reconstruction method preferred over another and if so, why? | | | P |  |  |  |
| C91.4 | Patient reported outcomes of different types of reconstruction | | | H | What are the clinical and patient-reported outcomes of different types of breast reconstruction procedure and how do they compare? | |  |
| **B9.3** | Pros and cons of different surgical and reconstructive options after breast surgery - for cancer patients and BRCA carriers. | | | H |  |  |  |
| **B170.6** | What are the advantages and disadvantages of different types of operations for breast cancer? | | | P |  |  |  |
| **B178.5** | What are the anticipated patient-reported outcomes of implant and flap reconstruction, and are these conveyed systematically to patients? | | | H |  |  |  |
| **69. What are the short and long-term outcomes of implant-based breast reconstruction with and without biological and synthetic mesh and how can these be improved?** | | | | | | | |
| C90.23 | Capsular Contracture: What is the best method of identifying the cause of a patient's capsular contracture? | | | P | How can the long-term complications of implant-based breast reconstruction (e.g. capsular contracture or implant malposition) be reduced? | | ***What are the short and long-term outcomes of implant-based breast reconstruction with and without biological and synthetic mesh and how can these be improved?*** |
| C90.24 | How can the incidences and/or severity of capsular contracture be reduced during and/or post-surgery? | | | P |  |  |  |
| C90.25 | Implant Movement / Rotation : What are the best methods to reduce implant movement and/or rotation? | | | P |  |  |  |
| **B110.13** | What are the long term risks for those who have a breast implant along with reconstruction & would follow up reduce risks | | |  |  |  |  |
| A30.2 | Breast reconstruction - how do synthetic and natural materials compare for those patients wanting breast reconstruction? | | |  | What are the short and long-term outcomes of implant-based breast reconstruction with biological and synthetic mesh? | |  |
| B101.5 | Long term outcomes and safety of use of AMD's/meshes. | | | H |  |  |  |
| B103.6 | Long-term outcomes of implant-based reconstructions (synthetic mesh vs ADM) | | | H |  |  |  |
| **70. Does prehabilitation and exercise improve outcomes for women having breast and reconstructive surgery for breast cancer?** | | | | | | | |
| **C130.10** | Understanding how best to optimise patients for breast and reconstruction surgery (prehab) | | | H | Does prehabilitation and exercise improve outcomes for women having breast and reconstructive surgery for breast cancer? | | ***Does prehabilitation and exercise improve outcomes for women having breast and reconstructive surgery for breast cancer?*** |
| **C170.17** | Can exercise prior to surgery be beneficial? | | | P |  |  |  |
| **71. What are the clinical and patient-reported outcomes of oncoplastic breast conserving procedures and do these differ in women at high risk of complications (smokers, women with high BMI)?** | | | | | | | |
| **B102.1** | extreme oncoplasty for tumours larger than 4 cm local perforator flaps therapeutic mammoplasty outcomes in high BMI patients like 30-35 BMI | | |  | What are the outcomes of oncoplastic breast conservation in women at high risk of complications (e.g. high BMI)? | | ***What are the clinical and patient-reported outcomes of oncoplastic breast conserving procedures and do these differ in women at high risk of complications (smokers, women with high BMI)?*** |
| **C63.3** | Collection of patient reported outcome measures will be very important for patients undergoing oncoplastic procedures (which is constantly evolving) to gain the patient’s perspective. Although recommended it is unclear how many breast units are routinely collecting PROMs data. | | | H | What are the patient reported outcomes of oncoplastic breast conserving procedures? | |  |
| B20.11* | Outcomes physical and psychological of local flaps | | | H |  |  |  |
| **72. How can we best support patients to make informed decisions about oncoplastic and reconstructive surgery including ensuring that they are offered appropriate balanced choices and have realistic expectations of outcomes?** | | | | | | | |
| **C24.5** | | Guidance on reconstruction choices (eg I had an immediate implant reconstruction, and mainly was told about how it would look - not how it would feel). | | P | | How can we best support patients to make informed decisions about oncoplastic and reconstructive surgery including ensuring that they are offered appropriate balanced choices (including no reconstruction) and have realistic expectations of outcomes? | ***How can we best support patients to make informed decisions about oncoplastic and reconstructive surgery including ensuring that they are offered appropriate balanced choices and have realistic expectations of outcomes?*** |
| **A6.1** | | Does having access to surgical photographs in the clinic help manage patient expectations over surgical outcomes or decisions over which surgery to choose? | | PO | |  |  |
| **B178.6** | | If they {PROMS for reconstruction} are {conveyed to patients systematically}, how do these data influence decision-making? | | H | |  |  |
| **C41.6** | | What is the best way of counselling patients about reconstructive options? | | H | |  |  |
| **B15.3** | | How can we prepare patients properly for DIEP reconstruction? I was not aware that my stomach area would be permanently numb. When I thought I had a small tumour on the right I opted for a mammoplasty. I underwent two breast preserving surgeries previous to my completion mastectomy with immediate DIEP. I would have opted for the double mastectomy from the start if I’d found out I had a tumour in each breast following my initial appointment. I attempted to say this when my bilateral diagnosis (IDC over 2cm in left and over 5cm in right) was confirmed by MRI a month after I presented with symptoms. However, my voice got lost as the surgeon and team were focussed on the breast preserving surgery I initially thought I wanted. I regret having to go through the mammoplasty then a re-excision neither of which got clear margins before the decision was made that I should have a mastectomy. | | PSOR | |  |  |
| **B111.2** | | To have time with the surgeon to understand fully all implications of surgery and what is meant by all options truthfully. For lobular that means accepting and explaining its uniqueness and why there may be need for further surgery. Explain reconstruction at a deeper level over a few appointments give people a chance where possible to digest info | | P | |  |  |
| **A118.1** | | Counselling & support re different surgery options including reconstruction. I had reconstructive surgery for breast cancer but following year it was not offered or discussed with my 74year old mum. Important to have discussion | | P | |  |  |
| **B46.3** | | Develop decision making tools to support more informed choices about reconstruction and oncoplastic conservation. At present there is a lack of realism about outcomes | | H | |  |  |
| **B161.6** | | How should the best option for reconstruction be discussed and decided? | | P | |  |  |
| **B90.10** | | Information To help patients make a more informed decision about which surgical procedure to choose, to manage their expectations, and to help make them feel more supported: What is the best way to provide patients with information on the different types of surgical options, what's involved in each, and before and after images of each? | | P | |  |  |
| **B179.2** | | How might my breast(s) look after different types of surgery? | | PO | |  |  |
| **B178.5** | | What are the anticipated patient-reported outcomes of implant and flap reconstruction, and are these conveyed systematically to patients? | | H | |  |  |
| **A178.2** | | How is information about reconstruction (immediate or delayed, implant or flap) conveyed at the time of diagnosis? | | H | |  |  |
| **B90.11** | | Can the feel (not just the look) of different reconstructions, particularly implant reconstructions, be simulated? | | P | |  |  |
| **B169.3** | | Is there enough information given about reconstruction...the fact that it is not a new breast and that it can go horribly wrong. Is it just the fashion. | | P | |  |  |
| **B157.4** | | More information is required before surgery. Share real life images and stories before surgical decisions are made. Fully list all the pros and cons of each option. | | H | |  |  |
| **B78.3** | | *as above - clear photos of what is involved in the surgery and in reality what you can expect your body to look like from reconstruction or no reconstruction | | P | |  |  |
| **C29.5** | | Outcome and side effects of surgery - better preparing people for the possibilities and what the signs and symptoms are | | P | |  |  |
| **C53.8** | | How can clearer information about complications and side effects be given? | | P | |  |  |
| **B106.4** | | Reassurance for patients about BIA ALCL concerns | | H | |  |  |
| **B90.13** | | Does a patient's lifestyle affect the type of surgery and/or reconstruction they choose to have and, if so, what lifestyle aspects in particular? | | P | |  |  |
| **A127.2** | | Would it be possible to develop a computer graphic of what a breast would look like after surgery so that people who are advised they will get a cosmetically poor result from a lumpectomy can make an informed decision on if it is the best option for them? This would also help patients know what to expect. | | P | |  |  |
| **B174.2** | | Understanding why women don't have a reconstruction (particularly immediate) when they are medically fit and it is offered. Is it time delays/availability of specialist services Is it too difficult to make a decision to seek extra surgery when recently diagnosed with cancer | | H | |  |  |
| **C41.5** | | Does the expertise of the breast surgeon make them bias when considering reconstructive or oncoplastic options with patients? | | H | |  |  |
| **B80.3** | | The options need to be given to the patient - all the different techniques available apart from the specific consultants expertise. | | R | |  |  |
| **B143.2** | | Surgeons may have a speciality in terms of reconstruction and could inadvertently recommend their preference or specialism rather than being more objective with the patient about their options. I think consideration and the discussion of the longer term impact of surgery prior to it happening would be useful. | | PR | |  |  |
| **A38.1** | | Why do Breast Units presume reconstruction is the norm? Why is no reconstruction not presented as an option? | | P | |  |  |
| **73. Does 3D scanning improve the outcomes of reconstructive breast surgery?** | | | | | | | |
| **B6.2** | Would 3D surface scanning of breast size/shape prior to surgery help with reconstructing a closer matching breast shape? | | | PO | Does 3D scanning improve the outcomes of reconstructive breast surgery? | | ***Does 3D scanning improve the outcomes of reconstructive breast surgery?*** |
| **74. Are the outcomes of breast reconstruction better if the procedure is performed by a breast or plastic surgeon?** | | | | | | | |
| A110.8 | Which patients receive the best breast reconstruction outcome eg by a general surgeon, breast surgeon or plastic surgeon | | |  | Are the outcomes of breast reconstruction better if the procedure is performed by a breast or plastic surgeon? | | ***Are the outcomes of breast reconstruction better if the procedure is performed by a breast or plastic surgeon?*** |
| **75. How can we reduce length of stay following tissue-based (autologous) breast reconstruction?** | | | | | | | |
| B119.8 | Breast Autologous reconstruction - how to limit time in hospital | | | H | How can we reduce length of stay following tissue-based (autologous) breast reconstruction? | | ***How can we reduce length of stay following tissue-based (autologous) breast reconstruction?*** |
| **76. Are patient-reported outcomes indicative of good quality breast reconstruction surgery?** | | | | | | | |
| B174.3 | Do we have enough PROMS data to ensure good quality reconstruction surgery is taking place - accepting the surgery is only part of a process - adequate workup, adequate counselling, good discussion of available options most likely to benefit the individual patient, skilled surgery and peri-operative management, appropriate aftercare, knowledge of complication rates and efforts to reduce to the lowest possible level. | | | H | Are patient-reported outcomes indicative of good quality breast reconstruction surgery? | | ***Are patient-reported outcomes indicative of good quality breast reconstruction surgery?*** |
| **77. How many units are routinely collecting patient reported outcomes following oncoplastic breast surgery and how should they be assessed?** | | | | | | | |
| **C63.3** | Collection of patient reported outcome measures will be very important for patients undergoing oncoplastic procedures (which is constantly evolving) to gain the patient’s perspective. Although recommended it is unclear how many breast units are routinely collecting PROMs data. | | | H | How many units are routinely collecting patient reported outcomes following oncoplastic breast surgery and how should they be assessed? | | ***How many units are routinely collecting patient reported outcomes following oncoplastic breast surgery and how should they be assessed?*** |
| **78. What are the outcomes of women electing to have a contralateral mastectomy for symmetry after breast cancer surgery and how can we best support women’s decision-making?** | | | | | | | |
| **B34.2** | To be told if it did come to a mastectomy and you can't get reconstruction they will not remove the other large breast but reduce it why ? Once I knew that I needed a mastectomy and the thought of living with one breast nearly destroyed me. | | | P | What are the outcomes of women electing to have a contralateral mastectomy for symmetry after breast cancer surgery and how can we best support women’s decision-making? | | ***What are the outcomes of women electing to have a contralateral mastectomy for symmetry after breast cancer surgery and how can we best support women’s decision-making?*** |
| **A36.3** | Why aren’t ladies offered the chance of flat symmetry at the time of diagnosis. | | | P |  |  |  |
| **B36.4** | Why is symmetrical flatness not talked about in this section? | | | P |  |  |  |
| **C47.2** | What are the outcomes for women who have an immediate bilateral mastectomy for symmetry? | | | H |  |  |  |
| **B47.1** | Are you doing the right thing by discouraging/not supporting women who request a bilateral mastectomy for symmetry? Could/should bilateral mastectomy for symmetry (immediate/delayed) be offered as an option to women alongside reconstruction? | | | H |  |  |  |
| **A48.1** | How can we be better supported to have our decision accepted to want to have a double mastectomy for symmetry? Why is this never an option when having an implant or tissue reconstruction is a longer recovery and comes with more risk and complications? | | | P |  |  |  |
| **79. When is symmetrisation or balancing surgery required and should it be performed at the same time as the breast cancer surgery or at a later date?** | | | | | | | |
| **B104.4** | management of the contralateral breast - surgical timing / staged vs synchronous surgery | | | H | When is symmetrisation or balancing surgery required and should it be performed at the time of the breast cancer surgery or at a later date? | | ***When is symmetrisation or balancing surgery required and should it be performed at the same time as the breast cancer surgery or at a later date?*** |
| **B140.9** | whether any treatment to the other breast might be appropriate for cosmetic reasons | | | P |  |  |  |
| **C42.2** | Core outcome set for breast cancer surgery-When is a breast reduction required? | | | H |  |  |  |
| **80. What impact does radiotherapy have on the outcomes of breast reconstruction and how does this vary by the type of reconstruction surgery performed (e.g. implant vs tissue-based procedures)?** | | | | | | | |
| **B13.3** | | | What impact on successful reconstruction does radiotherapy have? | P | What impact does radiotherapy have on the outcomes of (different types of) breast reconstruction?    ‘What is the best type of breast reconstruction if a patient needs radiotherapy?’ | | ***What impact does radiotherapy have on the outcomes of breast reconstruction and how does this vary by the type of reconstruction surgery performed (e.g. implant vs tissue-based procedures)?*** |
| **C39.4** | | | What are the long-term effects of radiotherapy in terms of scar healing and pain levels? | P |  |  |  |
| **B67.7** | | | Significance of breast fibrosis after different types of breast radiotherapy. | H |  |  |  |
| **B108.5** | | | Contention around reconstruction versus RT - how does this affect patients decisions and what limits their choices? There is still large variation in practice between units and surgeons as to best advice. Has anyone collected data on patient outcomes for those who have RT before after reconstruction. Do we know (research proven not anecdotally) if one type of recon is more affected by RT than another? Could partial breast RT make this worse, or accelerated RT? Types of recon can be heavily influenced by RT and timing can be affected too resulting in some patients been offered very limited surgical options. This introduces a lack of fairness and access to options. | H |  |  |  |
| **B95.3** | | | What is the optimal type of immediate/ delayed reconstruction if the patient needs radiotherapy? | H |  |  |  |
| **B103.4** | | | Impact of radiotherapy on overall outcome of immediate breast reconstruction (pre-pec vs sub-pec) | H | What is the impact of radiotherapy on the outcomes of prepectoral and subpectoral implant-based breast reconstruction | |  |
| **81. What is the best timing of breast reconstruction if a patient needs radiotherapy?** | | | | | | | |
| **B95.2** | | | What is the optimal timing of reconstruction if the patient needs radiotherapy? | H | What is the best timing of breast reconstruction if a patient needs radiotherapy? | | ***What is the best timing of breast reconstruction if a patient needs radiotherapy?*** |
| **82. Does requirement for post-mastectomy radiotherapy impact patients’ choices and decision making for breast reconstruction?** | | | | | | | |
| **B108.5** | | | Contention around reconstruction versus RT - how does this affect patients decisions and what limits their choices? There is still large variation in practice between units and surgeons as to best advice. Has anyone collected data on patient outcomes for those who have RT before after reconstruction. Do we know (research proven not anecdotally) if one type of recon is more affected by RT than another? Could partial breast RT make this worse, or accelerated RT? Types of recon can be heavily influenced by RT and timing can be affected too resulting in some patients been offered very limited surgical options. This introduces a lack of fairness and access to options. | H | Does requirement for PMRT impact patients’ choices and decision making for breast reconstruction? | | ***Does requirement for post-mastectomy radiotherapy impact patients’ choices and decision making for breast reconstruction?*** |
| **83. How do we develop national consensus on reconstruction and radiotherapy?** | | | | | | | |
| **B105.6** | | | How do we develop national consensus on reconstruction and radiotherapy? | H | How do we develop national consensus on reconstruction and radiotherapy? | | ***How do we develop national consensus on reconstruction and radiotherapy?*** |
| **84. Can we identify women at risk of poor outcomes following breast reconstruction and radiotherapy and explore ways these outcomes could be improved (e.g. different ways of giving radiotherapy)?** | | | | | | | |
| **B103.5** | | | Predictive models (genomics) of radiation toxicity on reconstruction / soft tissue / autologous vs implant-based | H | Can develop models to predict the effects of radiotherapy on different types of breast reconstruction? | | ***Can we identify women at risk of poor outcomes following breast reconstruction and radiotherapy and explore ways these outcomes could be improved (e.g. different ways of giving radiotherapy)*** |
| **B130.5** | | | Understanding how best to mitigate/ minimise the negative effects of RT on reconstruction whilst maintaining/ maximising oncological benefit | H | How can we improve the outcomes of breast reconstruction after post-mastectomy radiotherapy? (??different ways of giving radiotherapy) | |  |
| B19.8 | | | Better reconstruction, esp after radiotherapy | H |  |  |  |
| **85. How can we evaluate new techniques and devices for breast cancer surgery safely and effectively?** | | | | | | | |
| B130.6 | | | Evaluation of novel techniques / devices for reconstruction and breast cancer surgery safely | H | How can we evaluate new techniques and devices for breast surgery safely | | ***How can we evaluate new techniques and devices for breast cancer surgery safely and effectively?*** |
| **86. Is it possible to develop new options for breast reconstruction for women not suitable for implant reconstruction with insufficient fat for a tissue-based reconstruction?** | | | | | | | |
| B139.1 | | | For patients (likely in 20's, 30's) who have had mastectomy with delayed reconstruction, but are not suitable for implant based reconstruction and don't have much 'spare' tissue for autologous reconstruction, is there research into alternative suggestions for these patients? e.g. growing tissue in the lab, expanders used elsewhere on body, improved fat grafting, scaffold mesh to model breast shape etc? | P | Is it possible to develop new options for breast reconstruction for women not suitable for implant reconstruction with insufficient fat for a tissue-based reconstruction? | | ***Is it possible to develop new options for breast reconstruction for women not suitable for implant reconstruction with insufficient fat for a tissue-based reconstruction?*** |
| **87. Is a remote incision (i.e. periareolar) necessary or appropriate with reconstruction?** | | | | | | | |
| B177.7 | | | Is a remote incision(ie periareolar) necessary or appropriate with reconstruction. | H | Is a remote incision (ie periareolar) necessary or appropriate with reconstruction. | | ***Is a remote incision (ie periareolar) necessary or appropriate with reconstruction?*** |
| **88. Do we need a core outcome set for breast surgery?** | | | | | | | |
| **C42.2** | | | Core outcome set for breast cancer surgery- | H | Core outcome set for breast cancer surgery- | | ***Do we need a core outcome set for breast cancer surgery?*** |

***Questions about follow up; detection of recurrence and management of long-term complications of breast cancer surgery***

| ***Q no*** | | | | ***Raw data*** | | | ***Resp type*** | ***Indicative question(s)*** | | | | | | | ***Summary questions*** |
| --- | --- | --- | --- | --- | --- | --- | --- | --- | --- | --- | --- | --- | --- | --- | --- |
| **89. How can we best practically support patients around the time of their breast cancer surgery to ensure they are fully informed about the procedure; potential complications and feel confident before the operation and after discharge?** | | | | | | | | | | | | | | | |
| **A80.2** | | | | What follow up is there post appointment at the point of diagnosis. The patient normally takes a lot of information in but does not necessarily understand it all - they then return home and discuss with others who ask them questions which they do not have answers for. A few days after they feel clearer and may have further questions. | | | R | How can we best practically support patients around the time of their breast cancer surgery to ensure they are fully informed about the procedure and potential complications and feel confident before the operation and after discharge? | | | | | | | ***How can we best practically support patients around the time of their breast cancer surgery to ensure they are fully informed about the procedure; potential complications and feel confident before the operation and after discharge?*** |
| **C2.11** | | | | A simple flow chart showing who should be contacted if various issues arise would save time and worry for patients. | | | P |  |  |  |  |  |  |  |  |
| **C10.5** | | | | Patients need some sort of digital interface to help with general questions perioperatively | | | H |  |  |  |  |  |  |  |  |
| **C138.8** | | | | Research welfare of aftercare kits, canvas bags provided to hold draining bags discreetly, armpit cushions (I was donated these and they gave me some dignity) | | | P |  |  |  |  |  |  |  |  |
| **C111.3** | | | | Smaller drain pouches would be good and a breast nurse on call for advice would be helpful. Most people want to go home as soon as possible after so ensuring all is in place for discharge is good. I can’t really talk much about pain because I didn’t have an awful lot ( bilateral mastectomy) so didn’t use many meds. Found codiene helped with sleep in the first days after surgery. | | |  |  |  |  |  |  |  |  |  |
| **C29.4** | | | | Better support to be discharged home after surgery | | | P |  |  |  |  |  |  |  |  |
| **C66.4** | | | | Does being discharged immediately following surgery back to your own home and bed ensure a better psychological adjustment to having had breast cancer than a stay of a few days with support in hospital? | | | H |  |  |  |  |  |  |  |  |
| **C104.6** | | | | early discharge and care in the community - developing better continuity of care for district nurse or increased training for district nurses | | | H |  |  |  |  |  |  |  |  |
| **C119.11** | | | | Do women experience a support hiatus after discharge home? DOes it matter and how do we address it? | | | H |  |  |  |  |  |  |  |  |
| **C165.4** | | | | Having someone to talk to and go though any concerns especially directly after surgery while awaiting results of what comes next | | | P |  |  |  |  |  |  |  |  |
| **C169.4** | | | | Have you considered that the post-op specialist in-patient NURSING care is paramount. Just being chucked out after major surgery by non-specialist nurses is not good. Have you considered having a breast cancer ward or unit. Practicalities seem well taken care of but TLC in-patient care lacking perhaps. | | | P |  |  |  |  |  |  |  |  |
| **C134.1** | | | | Developing a standardised information sheet to be discussed with patients who are having lymph node surgery informing them of the risks of developing lymphoedema, the importance of post op skincare, exercise and maintaining a healthy weight and what to do if lymphoedema symptoms develop. | | | H |  |  |  |  |  |  |  |  |
| **C74.10** | | | | Preparing patients for the potential of further excision for better margins may help patients cope with the prospect of this if that is required. | | | P |  |  |  |  |  |  |  |  |
| **C88.3** | | | | Were patients adequately prepared for the experience and what information do the wish they had before coming to hospital | | | H |  |  |  |  |  |  |  |  |
| **90. What is the best approach to post-operative pain relief for women having breast cancer surgery?** | | | | | | | | | | | | | | | |
| **C94.5** | Management of immediate post operative pain for people with NSAID intolerance. Extent and prevalence of nerve block training for anaesthetic registrars. | | | | | | P | | | | What is the best approach to post-operative pain relief for women having breast cancer surgery? | | | | ***What is the best approach to post-operative pain relief for women having breast cancer surgery?*** |
| **C112.9** | Best form of drain management and pain relief | | | | | | P | | | |  |  |  |  |  |
| **B129.13** | Q. Why is pain relief not discussed at length, in a manner where doctors can holistically hear the symptoms of pain and calculate the correct medication in the earliest of stages, rather than misdiagnosing causing greater more painful side effects. | | | | | | PR | | | |  |  |  |  |  |
| **C140.13** | treatment for pain (and dressing management) | | | | | | P | | | |  |  |  |  |  |
| **C159.4** | As above. Appropriate and fast pain relief on hand - I was in the worst pain of my life, crying for help, after I came round from surgery and no one seemed to be able to help. Knowledge of who/what to ask for - it was only because I'd cared for a relative that I knew the hospital had a pain team and I asked to see them. Agreed pre-op procedures - the anaesthetist for my op didn't see me the night before and therefore I wasn't allowed any pre-op meds, although before I'd been admitted, this was promised. He brushed off my request for a pain block, saying I wouldn't need it. He apologised afterwards, when he read my post op notes - too late then of course. | | | | | | P | | | |  |  |  |  |  |
| **C178.7** | What is the evidence for different pain management approaches? | | | | | | H | | | |  |  |  |  |  |
| **91. How can rates of day case breast cancer surgery be increased?** | | | | | | | | | | | | | | | |
| **C16.6** | How to increase day care rate. | | | | | | H | | | | How can rates of day case breast cancer surgery be increased? | | | | ***How can rates of day case breast cancer surgery be increased?*** |
| **C20.13** | Day-case (use of intubation or not) | | | | | | H | | | |  |  |  |  |  |
| **92. Are dressings needed following breast cancer surgery and if so, which ones should be used and for how long?** | | | | | | | | | | | | | | | |
| **C38.5** | Why do some surgeons use dressings and others don’t? | | | | | | P | | | | Are dressings needed following breast cancer surgery and if so, which ones should be used and for how long? | | | | ***Are dressings needed following breast cancer surgery and if so, which ones should be used and for how long?*** |
| **C157.5** | What are the best dressings and drains. | | | | | | H | | | |  |  |  |  |  |
| **C140.13** | treatment for pain and dressing management | | | | | | P | | | |  |  |  |  |  |
| **C55.4** | When is best for dressings to be removed? | | | | | | P | | | |  |  |  |  |  |
| **C102.2** | role of negative pressure wound therapy | | | | | | H | | | |  |  |  |  |  |
| **C179.3** | What sort of dressings should I use to protect my wound, and will they be provided? | | | | | | PO | | | |  |  |  |  |  |
| **C166.2** | Use of medi-honey dressings to rapidly heal wounds (and keep infection free) | | | | | | PSR | | | |  |  |  |  |  |
| **93. How can we minimise wound complications following oncoplastic and reconstructive surgery?** | | | | | | | | | | | | | | | |
| **C6.4** | Are there ways to prevent/reduce scar stretching during healing after mastectomy? i.e. a less than 1mm incision width directly after surgery might end up as 5-10mm width scar once healed. | | | | | | PO | | | | How can we minimise wound complications following oncoplastic and reconstructive surgery? | | | | ***How can we minimise wound complications following oncoplastic and reconstructive surgery?*** |
| **C12.5** | Post-op wound healing particularly in relation to therapeutic mammoplasty which can delay start of adjuvant treatment. | | | | | | H | | | |  |  |  |  |  |
| **C103.10** | Role of negative pressure dressings in implant-based reconstructions (prevention of complications) - patient selection | | | | | | H | | | |  |  |  |  |  |
| **94. What happens if wounds do not heal following breast cancer surgery?** | | | | | | | | | | | | | | | |
| **C157.7** | What happens if the wound does not heal. | | | | | | H | | | | What happens if wounds do not heal following breast cancer surgery? | | | | ***What happens if wounds do not heal following breast cancer surgery?*** |
| **95. How can we reduce or prevent the development of seromas following breast cancer surgery and if they develop, how should they best be treated?** | | | | | | | | | | | | | | | |
| **C12.6** | Reduction of incidence of post-op seroma which can delay adjuvant radiotherapy | | | | | | H | | | | How can we reduce or prevent the development of seromas following breast cancer surgery? | | | | ***How can we reduce or prevent the development of seromas following breast cancer surgery and if they develop, how should they best be treated?*** |
| **C54.5** | More investigation into seromas and how to prevent them. | | | | | | P | | | |  |  |  |  |  |
| **C84.4** | What are the risk factors for the development of a seroma after breast cancer surgery involving removing lymph nodes; is the amount of drainage liquid straight after operation an indication of likely development and is the development of a seroma linked to the development of lymphoedema? | | | | | | P | | | |  |  |  |  |  |
| **C90.19** | Seroma: What is the best method (during and/or post-surgery) to minimise the amount of seroma? | | | | | | P | | | |  |  |  |  |  |
| **C112.8** | Seromas - what causes them; how to reduce likelihood of occurrence; how best to treat them; when to get extra help. | | | | | | P | | | |  |  |  |  |  |
| **C127.4** | Is it possible to predict who will get seromas or hematomas and have the management for these built into the patients treatment plan rather than patients having to rely on A&E and walk in centres to receive help for complication management and dressings? | | | | | | P | | | |  |  |  |  |  |
| **C157.6** | Should seromas be drained. | | | | | | H | | | | Should seromas be drained, if so when? | | | |  |
| **C170.13** | How soon can/should you get a seroma drained after developing one? | | | | | | P | | | |  |  |  |  |  |
| **96. Do we need to use drains following breast cancer surgery, if so, when, what type and how should they best be managed?** | | | | | | | | | | | | | | | |
| **C38.4** | Why do some hospitals use drains and others not? | | | | | | P | | | | Do we need to use drains following breast cancer surgery? | | | | ***Do we need to use drains following breast cancer surgery, if so, when, what type and how should they best be managed?*** |
| **C53.5** | Why do some health trust use drains and others do not. | | | | | | P | | | |  |  |  |  |  |
| **C41.4** | Is there a justifiable role for wound drains use in breast surgery? | | | | | | H | | | |  |  |  |  |  |
| **C55.3** | Is a drain necessary in all cases? | | | | | | P | | | |  |  |  |  |  |
| **C69.3** | RCT in drain no drain in breast surgery collaborative | | | | | | H | | | |  |  |  |  |  |
| **C72.10** | Drain or no drain? | | | | | | H | | | |  |  |  |  |  |
| **C85.10** | When should we use drains in non-complex, non-reconstructive surgery (e.g. after mastectomy or ANC)? | | | | | | H | | | |  |  |  |  |  |
| **C102.3** | routine use of drain after simple mastectomy | | | | | |  | | | |  |  |  |  |  |
| **C104.5** | drain usage and is it effective | | | | | | H | | | |  |  |  |  |  |
| **C178.8** | What is the evidence for drain use? | | | | | | H | | | |  |  |  |  |  |
| **C56.7** | The advantages vs disadvantages of having drains rather than pressure dressings. | | | | | | PSR | | | |  |  |  |  |  |
| **C109.5** | Better guidance regarding drains and when/if to use them. Are they strictly needed? And how do patients progress post surgically in those units that don't use them post-reconstruction. Best type of drain to use? Redivac or J-Vac? etc. | | | | | | H | | | | When are drains needed and what types of drains should be used? | | | |  |
| **C97.3** | Breast drains and dressing clinics - managed in the community setting or hospital outpatient setting? | | | | | | H | | | | What is the best management of drains and dressings following breast cancer surgery? | | | |  |
| **C83.2** | Is there a better way of draining, instead of carrying a little flowery bag . | | | | | | POR | | | |  |  |  |  |  |
| **C144.3** | How can drains after surgery be more manageable and less painful? | | | | | | P | | | |  |  |  |  |  |
| **C131.3** | Drain management | | | | | | P | | | |  |  |  |  |  |
| **C157.5** | What are the best dressings and drains. | | | | | | H | | | |  |  |  |  |  |
| **C149.3** | You can cope with the drains at home and if you are able early discharge is better as your own surroundings are better. Definitely insist on at home removal of drain (could be OOS pt experience) | | | | | | PSR | | | |  |  |  |  |  |
| **97. What is the best type of physiotherapy to help patients regain shoulder mobility following breast cancer surgery; how soon following surgery should it start and what is the best way of delivering treatment?** | | | | | | | | | | | | | | | |
| **C84.5** | | What type of (physio)therapy is most useful to retain or regain full shoulder movement after breast surgery with removal of lymph nodes and what is the best timing for this? | | | P | | | | What type of physiotherapy is best to regain full shoulder movement after breast cancer surgery | | | | ***What is the best type of physiotherapy to help patients regain shoulder mobility following breast cancer surgery; how soon following surgery should it start and what is the best way of delivering treatment?*** | | |
| **C32.6** | | Any developments in the arm exercise regimen after modified radical.mastectomy? | | | H | | | |  |  |  |  |  |  |  |
| **C113.6** | | Physios right away for shoulder mobility | | | P | | | | How soon following breast cancer surgery should physiotherapy start? | | | |  |  |  |
| **C170.15** | | How soon after surgery can you begin to exercise/commence physiotherapy? | | | P | | | |  |  |  |  |  |  |  |
| **C99.3** | | Does face to face physiotherapy after surgery (single or multiple appointments) improve mobility outcomes compared to videos/leaflets about exercises? | | | P | | | | Does face to face physiotherapy support results in better mobility outcomes than videos/written information in women having breast cancer surgery | | | |  |  |  |
| **98. What is the best way of supporting and proving follow up care for patients after breast cancer surgery and for how long should this be offered?** | | | | | | | | | | | | | | | |
| **C49.10** | | | | How long should a breast cancer patient who has undergone a single or double mastectomy be followed up post surgery? | | P | | | | How long should women be followed up after surgery for breast cancer and what should influence this? | | | | ***What is the best way of supporting and proving follow up care for patients after breast cancer surgery and for how long should this be offered?*** | |
| **A111.3** | | | | After five years you are discharged... why? Cancer rarely goes away fully and for lobular patients the stealth with which it moves around should indicate that it is already distributed to other places via lymph glands at a cellular level. Finally, if no further follow up how do begin to understand this unique subtype? How can we tell when our cancer returns? There is no solid tumour to feel. Countless times I encounter medical professionals who have no idea about lobular and don’t want to listen as I am only the patient. This is what makes me an advocate. I read peer reviewed papers, attend conferences, can critique evidence. Have even put together a lobular charity to help other women understand this diagnosis. Education about lobular needs to be more widely considered. | | P | | | |  |  |  |  |  |  |
| **C110.17** | | | | In those who are diagnosed young there is no support after discharge at 10 year mark. I feel as though I have been left high & dry! Where/what is the support? | |  | | | |  |  |  |  |  |  |
| **C98.4** | | | | How are patients doing on self managed follow up regimes - psychological and physical aspects could be researched. | | H | | | | What are the outcomes of self-managed follow up and how do these compare to women having clinical follow up after breast cancer surgery? | | | |  |  |
| **C93.3** | | | | More follow up appointments needed, rather than being discharged and left to manage on your own. | | P | | | |  |  |  |  |  |  |
| **C161.7** | | | | How should long term follow up be managed? | | P | | | | How should long-term breast cancer follow up be managed? | | | |  |  |
| **C112.10** | | | | Best form of follow up - community or back to hospital | | P | | | |  |  |  |  |  |  |
| **C119.12** | | | | How best to do lean follow-up from patient and doctor perspective | | H | | | |  |  |  |  |  |  |
| **C20.17** | | | | Phone v face to face support and follow up post operatively | | H | | | |  |  |  |  |  |  |
| **A160.1** | | | | For younger women there is often a battle to be scanned/ fears acknowledged so there should be specialist nurses for younger people. Scans/ mammogram results should be at a one stop appointment. Waiting increase anxiety. | |  | | | |  |  |  |  |  |  |
| **C172.3** | | | | how do I know if what my post-op experience is normal? best advice for getting on with my life | | H | | | | What is the best way to support women following breast cancer surgery in the long-term? | | | |  |  |
| **C49.12** | | | | Do you think that your local health board provides adequate emotional support to patients undergoing breast cancer surgery in the long term? | | P | | | |  |  |  |  |  |  |
| **C67.13** | | | | Managing patient's anxiety about relapse (including local recurrence) and having detailed personalised predictive models. | | H | | | |  |  |  |  |  |  |
| **C29.6** | | | | Supporting people who have issues well after treatment | | P | | | |  |  |  |  |  |  |
| **A33.1** | | | | Support for including physical activity as a follow up guide and social prescribing to certain groups | | PO | | | |  |  |  |  |  |  |
| **C121.2** | | | | Best method to forget the fear of breast cancer recurrence | | H | | | |  |  |  |  |  |  |
| **C145.2** | | | | Follow up support after treatment would have benefitted me. At the end of my treatment, I was scared, worried and very anxious. This was because all the staff involved in my treatment had been wonderful; I felt as if I'd been wrapped up in cotton wool and taken care of in the most sensitive and caring way. However, after treatment, there was nothing ; it was a lonely, vulnerable and extremely emotional period for me. This has eased overtime, although the anxiety hasn't, but it was a very difficult transition from in-treatment to out-of-treatment and recovery. Also, in terms of advances of prognosis/survival post treatment, could there be a way of keeping women who've been through treatment informed? I've been given my survival chances in the first ten years following treatment but I don't know if that could change depending on research and just generally more been known about breast cancer treatments and prognosis. I don't know how to go about finding out. Seven years on, I still worry about it a lot. I see things on social media that lead to forum discussions, but it's all aimed at women whose cancers have advanced. I understand only too well that they need a lot of discussion and support and I hope that will never be me, but I don't know that and a support network of cancer survivors, like me, who are just getting on with their lives would be wonderful. | | P | | | |  |  |  |  |  |  |
| **C110.16** | | | | Does long term follow up (for cancer survivors) improve outcomes for patients? | |  | | | | What are the long-term outcomes of women having breast cancer surgery and are they improved by long-term follow up? | | | |  |  |
| **C108.7** | | | | Are we increasing GP burden? Or should we consider follow up regimes as in other countries were by GPs do all follow up? Extended endocrine therapies and new targeted therapies have increased side effects ad burden. More real world research is required to ascertain the extent of long term issues and the patient experience. They are living longer with more and more side effects. Some even report wishing they'd not had treatments and need to balance quality and quantity of life | | H | | | |  |  |  |  |  |  |
| **99. What ways can surgeons use to understand the outcomes of their breast cancer surgery without seeing patients for clinical follow up?** | | | | | | | | | | | | | | | |
| **C119.13** | | | | How do surgeons (in no follow-up culture) understand the consequences of their surgery? Video/photo appraisal of cosmesis vs. face to face, can remote review work? | | H | | | | What ways can surgeons use to understand the outcomes of their breast cancer surgery without seeing patients for clinical follow up? | | | | ***What ways can surgeons use to understand the outcomes of their breast cancer surgery without seeing patients for clinical follow up?*** | |
| **100. What is the best method of surveillance to detect breast cancer recurrence and how does this vary according to patient (e.g. age, breast density) or breast cancer related factors?** | | | | | | | | | | | | | | | |
| **A27.2** | | | | What is the best method of surveillance including imaging for women who have been diagnosed with breast cancer in their 30s? | | H | | | | What is the best way to detect recurrence and how does this vary according to patient or breast cancer factors? | | | | ***What is the best method of surveillance to detect breast cancer recurrence and how does this vary according to patient (e.g age, breast density) or breast cancer related factors?*** | |
| **A103.3** | | | | effectiveness of mammographic surveillance in young patients/dense breast post BCT / detection of recurrence etc. | | H | | | |  |  |  |  |  |  |
| **C84.6** | | | | How can diagnosis of recurrences be improved for large-breasted women where it is unlikely that lumps can be palpated? I had a 35mm cancer lump that even the surgeon could not find. | | P | | | |  |  |  |  |  |  |
| **C101.8** | | | | Evidence of imaging follow up post breast cancer treatment. Is this required. | | H | | | |  |  |  |  |  |  |
| **A90.2** | | | | Does offering supplemental screening (in addition to a mammogram) as standard practice to women with dense breasts reduce the number of late stage diagnoses and/or help earlier detection of a recurrence? | | P | | | | Does supplemental screening in women with dense breasts reduce the number of later stage diagnoses and/or help earlier detection of recurrence | | | |  |  |
| **101. What factors increase the risk of breast cancer recurrence after surgery and is it possible to predict what patients are at higher risk due to patient or breast cancer related factors to help them make a more informed decision about breast cancer surgery?** | | | | | | | | | | | | | | | |
| **B8.3** | | | | Do multiple operations and anaesthetics increase the risk of recurrence and metastasis? | | H | | | | What factors increase the risk of breast cancer recurrence and is it possible to predict what patients are at higher risk due to patient or breast cancer related factors to help them make a more informed decision about breast cancer surgery? | | | | ***What factors increase the risk of breast cancer recurrence after surgery and is it possible to predict what patients are at higher risk due to patient or breast cancer related factors to help them make a more informed decision about breast cancer surgery?*** | |
| **A82.2** | | | | How to detect breast cancers that come back quickly | | H | | | |  |  |  |  |  |  |
| **B130.8** | | | | Understanding if there are somatic cancer genomic predictors of surgical outcomes such as local recurrence and how these should influence surgical treatments | | H | | | |  |  |  |  |  |  |
| **A90.3** | | | | What percentage of women with dense breasts recur after a breast cancer diagnosis and how does this compare to women with non-dense breasts? | | P | | | |  |  |  |  |  |  |
| **A122.1** | | | | Predicting risk of death by cancer type predicting risk of recurrence, and making this data available. I think that we are remiss in not systematically analysing data on women who get second cancers. For me, this would be a key piece of information on treatment decisions, and we are seeing a lot of cancer in older women who have been treated for breast cancer in the past. The success of modern treatment means that this is increasingly likely. I would rather have a mastectomy at 60 than a WLE at 60 and a mastectomy at 80. | | H | | | |  |  |  |  |  |  |
| **B127.3** | | | | Would it be possible to have a model (like NHS Predict) into which you could put your breast cancer details and see the percentages of survival / risk of recurrence/ need for further surgery for different options of surgery, timing in relation to other treatments. e.g. If you are offered chemotherapy first to shrink the lump what are the chances that i would lead to a lumpectomy rather than a mastectomy? | | P | | | |  |  |  |  |  |  |
| **C177.9** | | | | What is impact of High BMI, diabetes on infection rates after surgery, postoperative stay and recovery and longterm oncological outcomes? | | H | | | |  |  |  |  |  |  |
| **C5.2** | | | | The impact of exercise, diet, alcohol, smoking and mental well being on recovery from surgery, long term recurrence rates / survival. Do exercise programmes / prehabilitation programmes increase survival in breast | | H | | | |  |  |  |  |  |  |
| **102. What are the best surgical options for patients who get locoregional recurrence of their breast cancer following surgery?** | | | | | | | | | | | | | | | |
| **B130.9** | | | | Optimal surgery for locoregional recurrence | | H | | | | What are the best surgical options for patients who get locoregional recurrence of their breast cancer following surgery? | | | | ***What are the best surgical options for patients who get locoregional recurrence of their breast cancer following surgery?*** | |
| **103. What is the risk of complications following breast surgery and how is this impacted by obesity and other patient factors?** | | | | | | | | | | | | | | | |
| **C7.5*** | | | What is the risk of complications after breast cancer surgery? | | |  | | | | What is the risk of complications following breast surgery and how is this impacted by obesity and other patient factors? | | ***What is the risk of complications following breast surgery and how is this impacted by obesity and other patient factors?*** | | | |
| **C95.4** | | | How can we better identify patients at risk of complications and side-effects, in order to target support or interventions, or if appropriate, change treatment plan? | | | H | | | |  |  |  |  |  |  |
| **C130.11** | | | How to minimise the impact of obesity and other patient factors on outcomes | | | H | | | |  |  |  |  |  |  |
| **C177.9** | | | What is impact of High BMI, diabetes on infection rates after surgery, postoperative stay and recovery and longterm oncological outconmes? | | | H | | | |  |  |  |  |  |  |
| **104. What causes cording following breast cancer surgery, how many women get it and how can it best be prevented or treated?** | | | | | | | | | | | | | | | |
| **C8.5** | | | How can you prevent cording? | | | H | | | | How can you prevent cording following breast cancer surgery? | | ***What causes cording following breast cancer surgery, how many women get it and how can it best be prevented or treated?*** | | | |
| **C135.3** | | | There is far too little known about axillary webbing / cording and how it should be best managed. Opinions vary from gentle exercise / physio to more vigorous breakdown. There is almost nothing about the aetiology / causative factors or interventions. | | | H | | | | What causes cording following breast cancer surgery and how should this be managed? | |  |  |  |  |
| **C132.5** | | | cording incidence and how this should be managed. | | | H | | | | How many women experiencing cording following breast cancer surgery and how and when should this best be managed? | |  |  |  |  |
| **C9.8** | | | Number of patients experiencing cording after breast surgery/lymph node removal and timeliness of interventions to treat this | | | H | | | |  |  |  |  |  |  |
| **C74.9** | | | Knowing how to manage cording and the role of physiotherapy would be useful. | | |  | | | |  |  |  |  |  |  |
| **C106.5** | | | Painful cording | | | H | | | |  |  |  |  |  |  |
| **105. How many women develop lymphoedema following breast cancer surgery, why do they develop it and how can we prevent or reduce the risk of lymphoedema in women having breast cancer surgery?** | | | | | | | | | | | | | | | |
| **C90.20** | | | Lymphoedema: What percentage of patients get lymphoedema as a result of breast cancer surgery / treatment, by type of surgery, extent of surgery, type of reconstruction, and location of the lymphoedema (arm only; arm and breast / chest; or breast / chest only)? | | | P | | | | How many women get lymphoedema following breast cancer surgery and how is this influenced by the type of surgery performed? | | ***How many women develop lymphoedema following breast cancer surgery, why do they develop it and how can we prevent or reduce the risk of lymphoedema in women having breast cancer surgery?*** | | | |
| **C90.21** | | | Has the number of cases of breast / chest lymphoedema following breast cancer surgery / treatment increased in recent years and if so, what is the cause of this increase? | | | P | | | | Are the numbers of women experiencing lymphoedema after breast cancer surgery increasing and if so why? | |  |  |  |  |
| **A132.1** | | | Can we find out if patients are at high risk of developing lymphoedema? for example could we do ICG scans on patients prior to surgery to identify how their lymph system drains, and then repeat this after their surgery/radiotherpay to see if the lymph is draining out of their arm/breast, and idenfity lymphoedmea in its early stages or those most at risk, who could then maybe wear sleeves. | | | H | | | | Can we identify women at high-risk of lymphoedema? | |  |  |  |  |
| **C32.7** | | | How to prevent post MRM lymphedema of the limb? | | | H | | | | How can we prevent lymphoedema after breast cancer surgery? | |  |  |  |  |
| **B67.8** | | | Surgical techniques to reduce the potential for lymphoedema. | | | H | | | |  |  |  |  |  |  |
| **C90.22** | | | How, surgically, can the risk and/or effect of lymphoedema be reduced, including breast / chest lymphoedema? | | | P | | | |  |  |  |  |  |  |
| **C103.8** | | | Prevention of lymphoedema - role of reverse axillary mapping, ICG | | | H | | | |  |  |  |  |  |  |
| **B105.5** | | | How can we reduce the risk of lymphoedema? | | | H | | | |  |  |  |  |  |  |
| **C30.6** | | | Best ways for breast surgery to be conducted to avoid lymphedema after breast cancer operation. | | | PO | | | |  |  |  |  |  |  |
| **B132.4** | | | Reducing risk of lymphoedema, surgical and non surgical options need to be considered. this is a significant consequence to breast cancer treatment that is overlooked in the research field. this needs to include breast and arm lymphoedema. there is research already showing the impact on QOL, and the morbidity this causes. but there is nothing looking at minimising risk, or proactive interventions like LVA or lipofilling axilla to minimise risk (please consider this). | | | H | | | |  |  |  |  |  |  |
| **106. What is the long-term impact of lymphoedema on women who develop it following breast cancer surgery and how is lymphoedema best managed?** | | | | | | | | | | | | | | | |
| **C78.10** | | | *long term impact of lymphoedema | | | P | | | | What is the long-term impact of lymphoedema? | | ***What is the long-term impact of lymphoedema on women who develop it following breast cancer surgery and how is lymphoedema best managed?*** | | | |
| **C132.7** | | | research into what to expect if lymphoedema develops. | | | H | | | |  |  |  |  |  |  |
| **A180.1** | | | Improved management of treatment side effects: ET side effects Shoulder mobility Lymphoedema | | | H | | | | How should we best manage lymphoedema following breast cancer surgery? | |  |  |  |  |
| **C132.8** | | | how lymphoedema should be managed when a consequence of breast cancer treatment. | | | H | | | |  |  |  |  |  |  |
| **C151.3** | | | I guess what worries me most is the lack of knowledge and treatment for lymphodema | | | P | | | |  |  |  |  |  |  |
| **C107.3** | | | Management of lymphedema | | | H | | | |  |  |  |  |  |  |
| **C104.7** | | | managing breast Lymphoedema following WLE and RT | | | H | | | |  |  |  |  |  |  |
| **C65.3** | | | Better management of nerve damage to the under arm, even after sentinel lymph node biopsy. Why not teach patients how to do lymphodema massage? | | | P | | | | Should all patients be taught lymphoedema massage? | |  |  |  |  |
| **C105.7** | | | How do we develop an integral lymphoedema management plan in the breast cancer clinic? | | | H | | | | What is the best model of care for women who develop lymphoedema after breast cancer surgery? | |  |  |  |  |
| **C99.6** | | | What model of lymphoedema care leads to the best outcomes - emotionally and physically? | | | P | | | |  |  |  |  |  |  |
| **C99.5** | | | Does active monitoring for lymphoedema by clinicians reduce stress (as opposed to patients looking out for, and wondering about, possible signs)? | | | P | | | |  |  |  |  |  |  |
| **C99.4** | | | Does early identification of post-surgical lymphoedema lead to better outcomes? | | | P | | | | Does early identification of lymphoedema results in better outcomes? | |  |  |  |  |
| **C132.6** | | | incidence of cellulitis in arms and breast that is usually is associated with lymphoedema. how this can be best managed/minimised in line with antimicrobial stewardship. | | | H | | | | What proportion of women with lymphoedema develop cellulitis and how this can best be managed | |  |  |  |  |
| **107. What is the role and outcomes of surgery to treat lymphoedema?** | | | | | | | | | | | | | | | |
| **A132.3** | | | Can we look at lipofilling the axilla to reduce incidence axillary vein collapse that causes a dramatic onset of lymphoedema post op sometimes due to venous obstruction in axilla. | | | H | | | | What is the role and outcomes of surgery to treat lymphoedema? | | ***What is the role and outcomes of surgery to treat lymphoedema?*** | | | |
| **A132.2** | | | Also if patients are identified at high risk, can we then research offering them LVA surgery? lymph venous anastomosis (Oxford lymphoedema Practice) lead on this, as either early intervention/minimising development of lymphoedema. | | | H | | | |  |  |  |  |  |  |
| **B101.6** | | | results of surgical interventions for lymphoedema | | | H | | | |  |  |  |  |  |  |
| **108. How can chronic (long-term) pain following breast cancer surgery be prevented?** | | | | | | | | | | | | | | | |
| **C67.10** | | | Best anaesthetic/LA/block practice for preventing postoperative persistent pain. | | | H | | | | How can chronic pain following breast cancer surgery be prevented? | | ***How can chronic pain following breast cancer surgery be prevented?*** | | | |
| **C108.6** | | | Post-operative pain is an issue that is ignored. Lack of follow up leads to a false assumption that chronic pain does not exist in this patient group. It is highly prevalent and causes lots of issues especially relating to QoL and functionality. More research is needed on long term outcomes as well as monitoring of short term outcomes (as we know they are linked). Prevention of or better monitoring of short term pain could prevent persistent pain. Self-management is limited as patients have so little if any follow up and attendance at 'follow-up' programmes is very poor with less than 50% attending these - so how are patients going to effectively self-manage if they do not get this training and information? | | |  | | | |  |  |  |  |  |  |
| **109. What is the best management for long-term (chronic) post-surgical pain or nerve damage in women who have had breast cancer surgery?** | | | | | | | | | | | | | | | |
| **C56.9** | | | Phantom breast pain following mastectomy, why is it not discussed prior to surgery? How common is phantom breast pain? How can it be managed? | | | PSR | | | | What is the best management for long-term (chronic) post-surgical pain in women who have had breast cancer surgery? | | ***What is the best management for long-term (chronic) post-surgical pain or nerve damage in women who have had breast cancer surgery?*** | | | |
| **C9.10** | | | Level of pain/restrictions 12 months post surgery | | | H | | | |  |  |  |  |  |  |
| **C16.7** | | | Long term post treatment pain, both counselling and treatment. | | | H | | | |  |  |  |  |  |  |
| **C18.7** | | | Treatment for breast pain after surgery | | | H | | | |  |  |  |  |  |  |
| **C19.10** | | | Long term pain | | | H | | | |  |  |  |  |  |  |
| **C20.12** | | | Pain short term and longterm | | | H | | | |  |  |  |  |  |  |
| **C96.3** | | | Trial looking at different non- drug methods of treating nerve pain. | | | H | | | |  |  |  |  |  |  |
| **C98.5** | | | How are GP's managing symptoms of pain following breast cancer diagnosis and treatment - do they refer back to original breast cancer team or re-refer to different team - questions around anxiety and continuity would be key to this type of research question. | | | H | | | |  |  |  |  |  |  |
| **C101.7** | | | Management of chronic post breast surgery pains, can we reduce or avoid this developing | | | H | | | |  |  |  |  |  |  |
| **C170.18** | | | Are there any non medicinal ways to manage pain, such as hypnotherapy, binaural beats, reflexology etc? | | | P | | | |  |  |  |  |  |  |
| **C108.6** | | | Post-operative pain is an issue that is ignored. Lack of follow up leads to a false assumption that chronic pain does not exist in this patient group. It is highly prevalent and causes lots of issues especially relating to QoL and functionality. More research is needed on long term outcomes as well as monitoring of short term outcomes (as we know they are linked). Prevention of or better monitoring of short term pain could prevent persistent pain. Self-management is limited as patients have so little if any follow up and attendance at 'follow-up' programmes is very poor with less than 50% attending these - so how are patients going to effectively self-manage if they do not get this training and information? | | |  | | | |  |  |  |  |  |  |
| **C109.4** | | | Post reconstructive pain and understanding it better. | | | H | | | |  |  |  |  |  |  |
| **C128.6** | | | What treatment can be given for long term problems either with pain syndrome or shoulder dysfunction? | | | H | | | |  |  |  |  |  |  |
| **C166.3** | | | Post-op effects of pain/numbness at site circa 12-36 months after a lumpectomy | | | PSR | | | |  |  |  |  |  |  |
| **C99.2** | | | What is the rate of winged scapula after mastectomy, and what are the best ways of preparing people for the possibility, treatment options if any, and support? | | | P | | | | What is the best management of post-operative nerve damage (e.g. loss of function or numbness) after breast cancer surgery | |  |  |  |  |
| **C153.4** | | | Complications and how to pick up and deal with quickly e.g I have permanent nerve damage which means I can’t lift my arm over my head and no one picked this up, it’s only when I noted a few years later to my gp did I have tests to confirm extent of damage | | | P | | | |  |  |  |  |  |  |
| **C161.9** | | | How do you make sure quality of life is preserved post op - dealing with altered sensations and psychological issues and pain? | | | P | | | |  |  |  |  |  |  |
| **110. Could 3D scanning be used to improve prostheses for women having breast cancer surgery?** | | | | | | | | | | | | | | | |
| **B6.3** | | | Would giving patients the option to have 3D breast scanning prior to surgery in order to have that data stored and available for making better matching prostheses help with self confidence after surgery? | | | PO | | | | Could 3D scanning be used to improve prostheses for women having breast cancer surgery? | | ***Could 3D scanning be used to improve prostheses for women having breast cancer surgery?*** | | | |

***Questions about patients at high risk of developing breast cancer***

| ***Q no*** | ***Raw data*** | ***Resp type*** | ***Indicative question(s)*** | ***Summary questions*** | |
| --- | --- | --- | --- | --- | --- |
| **111. Can we develop better ways of picking up women at high risk of developing breast in breast clinics?** | | | | | |
| **A89.1** | Better assessment in clinics at picking up high risk patients | H | Can we develop better ways of picking up women at high risk of developing breast in breast clinics? | ***Can we develop better ways of identifying women at high risk of developing breast in breast clinics?*** | |
| **112. What type of imaging should women at high risk of developing breast cancer have; when should it start and how frequently should it be performed?** | | | | | |
| **A157.2** | For women with a strong family history of breast cancer at what age should screening start. | H | When should screening for women at high risk of developing breast cancer start? | ***What type of imaging should women at high risk of developing breast cancer have; when should it start and how frequently should it be performed?*** | |
| **A32.2** | What should be the age at which screening should be started for the high risk group? | H |  |  |  |
| **A123.2** | Should high-risk people start screening sooner? | P |  |  |  |
| **A18.1** | What is the best interval for mammograms for people with a family history/ breast cancer/atypia? | H | How frequently should women at high risk of breast cancer have imaging? |  |  |
| **A32.1** | What should be the frequency of screening mammograms/ ultrasound/ MR mammogram for women who have a high risk of developing breast cancer? | H |  |  |  |
| **C40.6** | Also scanned [RRM patients] as breast tissue can grow back? | R | What is the best type of surveillance for women at high genetic risk of developing breast cancer? |  |  |
| **A70.1** | How best to surveillance the breast in those at high risk? | H |  |  |  |
| **113. How can we help women at risk of breast cancer make an informed choice about breast cancer screening options?** | | | | | |
| **A15.2** | How can we ensure that patients understand risks and benefits of each screening option to enable them to make an informed choice and not have the choice made for them. | PSOR | How can we help women at risk of breast cancer make an informed choice about breast cancer screening options? | ***How can we help women at risk of breast cancer make an informed choice about breast cancer screening options?*** | |
| **114. Are there alternatives to imaging in high risk women not having imaging due to pregnancy/breast feeding?** | | | | | |
| **A40.1** | In spite of monitoring many young woman who have the gene are missed annually through pregnancy and then breastfeeding. Is there another method of checking so these woman are not repeatably missed. | R | Are there alternatives to imaging in high risk women not having imaging due to pregnancy/breast feeding? | ***Are there alternatives to imaging in high risk women not having imaging due to pregnancy/breast feeding?*** | |
| **115. How can breast density and risk of breast cancer best be discussed with patients?** | | | | | |
| **A15.1** | Why are people not educated about breast density and the dual issues of raised risk and masking effect on mammogram? | PSOR | How can breast density and risk best be discussed with patients? | | ***How can breast density and risk of breast cancer best be discussed with patients?*** |
| **A157.3** | Should breast density be discussed with women after a mammogram explaining how it is linked with risk. | H |  |  |  |
| **A11.1** | People at high risk of breast cancer should be told in the results of their mammograms, of their breast density if applicable, as this is now a big feature in prevention of breast cancer, and this information is Vital to the patient, as is the right to know as soon as it is discovered in the breast. I was never informed of my own density, and was diagnosed with breast cancer after undergoing a risk reducing bilateral mastectomies | PSR |  |  |  |
| **116. Does supplemental screening in women with dense breasts reduce the number of later stage diagnoses and/or help earlier detection of recurrence?** | | | | | |
| **A90.2** | Does offering supplemental screening (in addition to a mammogram) as standard practice to women with dense breasts reduce the number of late stage diagnoses and/or help earlier detection of a recurrence? | P | Does supplemental screening in women with dense breasts reduce the number of later stage diagnoses and/or help earlier detection of recurrence | | ***Does supplemental screening in women with dense breasts reduce the number of later stage diagnoses and/or help earlier detection of recurrence?*** |
| **117. Would risk stratification using breast density and family history allow breast cancers to be detected at an earlier stage and would this be cost-effective?** | | | | | |
| **A90.4** | Risk stratification: How many breast cancers would be diagnosed at an earlier stage if women were risk stratified based on their family history (non-BRCA) and breast density? | P | Would risk stratification using breast density and family history allow breast cancers to be detected at an earlier stage? | | ***Would risk stratification using breast density and family history allow breast cancers to be detected at an earlier stage and would this be cost-effective?*** |
| **A90.6** | Imaging and biopsy: What effect would considering an individual's family history and/or breast density have on the category assigned to an individual’s mammogram and the recommended action to take? | P |  |  |  |
| **A90.5** | What is the cost-benefit (monetary and patient quality of life) of such risk stratification vs later stage diagnosis and subsequent more extensive treatment? | P | What is the cost-benefit of risk-stratification vs later diagnosis | |  |
| **118. What are the options for managing women at high risk of developing breast cancer?** | | | | | |
| **A168.3** | Options for people at high risk, for managing that risk? | P | What are the options for managing women at high risk of developing breast cancer? | | ***What are the options for managing women at high risk of developing breast cancer?*** |
| **A19.1** | Prevention for high risk people | H |  |  |  |
| **A30.3** | Is mastectomy for younger women the best/only way to treat patients with a history of breast cancer in their family? | PO |  |  |  |
| **C85.9** | What are the long-term quality of life consequences for these {non-BRCA risk reducing} patients? | H |  |  |  |
| **119. When should endocrine therapy be used to reduce breast cancer risk?** | | | | | |
| **A20.3** | Use of medications to lower risk | H | When should endocrine therapy be used to reduce breast cancer risk? | | ***When should endocrine therapy be used to reduce breast cancer risk?*** |
| **A108.4** | Uptake rates for preventative endocrine therapy in FH patients is very poor - why is this and what can be done to improve it? | H |  |  |  |
| **A4.3** | Supportive programmes to help women engaging with breast cancer prevention medications | H |  |  |  |
| **A180.2** | Role of ET in low risk disease eg atypia / LGDCIS | H |  |  |  |
| **120. What are the risks and benefits of using endocrine therapy to reduce breast cancer risk and how should this be communicated to patients?** | | | | | |
| **A81.1** | Further understanding of what are the indications for chemoprophylaxis for breast cancer and quantification of the risk and benefits of this approach | H | What are the risks and benefits of using endocrine therapy to reduce breast cancer risk and how should this be communicated to patients? | | ***What are the risks and benefits of using endocrine therapy to reduce breast cancer risk and how should this be communicated to patients?*** |
| **A75.5** | What is uptake of chemo-prevention in the moderate risk group and how is the best way to communicate this in affair and reasoned manner? | H |  |  |  |
| **121. How can we increase the use of endocrine therapy to reduce the risk of breast cancer in appropriate groups?** | | | | | |
| **A68.1** | As a National champion for the tamoxifen uptake study I would be keen to look at ways tamoxifen can be promoted to reduce the risk of breast cancer in intermediate and high risk women. This would also include research into how breast density on mammography can be utilised to identify these probands. | H | How can we increase the use of endocrine therapy to reduce the risk of breast cancer in appropriate groups? | | ***How can we increase the use of endocrine therapy to reduce the risk of breast cancer in appropriate groups?*** |
| **122. What is the best way of supporting women at high-risk of breast cancer to make decisions about surgery and then after surgery has been performed?** | | | | | |
| **A103.1** | Counselling methods and long-term psychological impact of risk-reducing surgery on high risk patients | H | What is the best way of supporting women at high-risk of breast cancer to make decisions about surgery and how should they be followed up after surgery has been performed? | | ***What is the best way of supporting women at high-risk of breast cancer to make decisions about surgery and then after surgery has been performed?*** |
| **A108.2** | The variation in counselling for high risk FH patients around the UK - do people actually receive any counselling?? | H |  |  |  |
| **A108.3** | Does this variation {in support/counselling} affect the rates of those who go on to have surgery or not? | H |  |  |  |
| **A109.1** | Best models of managing the pre-operative pathway in BRCA carriers? i.e. Key issues for clinical psychologists to discuss. | H |  |  |  |
| **A70.3** | Should risk reducing patients have confidential discussion with another pt who has had the same risk reducing surgery before committing to a definitive type of reconstruction? |  |  |  |  |
| **C40.4** | Once preventative surgery is completed some sort of follow up with counselling should be completed. I have 3 close family members and they have been left to suffer the consequences of mishandled preconselling and 2 from bad bed side manner from their surgeon. Also giving them a point of contact post op would be nice instead of being dropped like a hot potato. | R | How should women at high risk of breast cancer be followed up after risk-reducing surgery? | |  |
| **123. What are the risks and benefits of mastectomy with or without breast reconstruction in women at high breast cancer risk and when should surgery be performed?** | | | | | |
| **B9.3** | Pros and cons of different surgical and reconstructive options after breast surgery - for cancer patients and BRCA carriers. | H | What are the pros and cons of different surgical and reconstructive options for women carrying the BRCA gene? | | ***What are the risks and benefits of mastectomy with or without breast reconstruction in women at high breast cancer risk and when should surgery be performed?*** |
| **A103.2** | Timeliness of risk-reducing surgery in gene mutation carriers including less prevalent variants/mutations (with and without previous history of breast cancer) | H | When should risk-reducing surgery be performed? | |  |
| **A91.1** | When is the best timing of surgery and what should the delay be between BRCA diagnosis and treatment | H |  |  |  |
| **A41.1** | Should there be an appropriate age from which someone with gene mutation can reasonably consider having surgery. | H |  |  |  |
| **A70.2** | How long should risk reducing patients consider the surgical options before committing to a final plan? | H |  |  |  |
| **124. Which women at high risk but who do not carry the BRCA gene would benefit from risk reducing surgery?** | | | | | |
| **C85.8** | Which non-BRCA patients benefit from risk reducing surgery? | H | Which non BRCA patients would benefit from risk reducing surgery? | | ***Which women at high risk but who do not carry the BRCA gene would benefit from risk reducing surgery?*** |
| **125. Does risk reducing surgery improve survival in women at risk of developing breast cancer?** | | | | | |
| **A101.1** | Does risk reducing mastectomy improve survival | H | Does risk reducing surgery improve survival? | | ***Does risk reducing surgery improve survival in women at risk of developing breast cancer?*** |
| **126. Can weight management and lifestyle advice for patients at high-risk reduce breast cancer risk?** | | | | | |
| **A4.1** | Introducing services to help high risk people identify & manage their lifestyle risk factors | H | Can weight management and lifestyle advice for patients at high-risk reduce breast cancer risk? | | ***Can weight management and lifestyle advice for patients at high-risk reduce breast cancer risk?*** |
| **A4.2** | Weight management for high risk women | H |  |  |  |
| **A75.1** | Does lifestyle advice provided in Family history clinics encourage people to adopt a healthier lifestyle to reduce their risk? | H |  |  |  |
| **127. How can we improve engagement of ethnic and lower socioeconomic groups with high-risk clinics?** | | | | | |
| **A4.4** | Engaging different ethnic groups and lower socio economic groups with high risk clinics | H | How can we improve engagement of ethnic and lower socioeconomic groups with high risk clinics? | | ***How can we improve engagement of ethnic and lower socioeconomic groups with high-risk clinics?*** |
| **128. What is the best way of communicating future breast cancer risk in patients with genetic risk factors or a previous breast cancer?** | | | | | |
| **A75.4** | What is the best way to communicate future breast cancer risk to the unaffected individual? | H | What is the best way of communicating future breast cancer risk in patients with genetic risk factors or a previous breast cancer? | | ***What is the best way of communicating future breast cancer risk in patients with genetic risk factors or a previous breast cancer?*** |
| **A178.1** | What information on the risk of developing breast cancer as a primary cancer, or developing contralateral cancer, in the general population and in those with genetic susceptibility, is available, and what is conveyed? | H |  |  |  |
| **A109.2** | The development of a national information pack for moderate risk carriers as opposed to just regional guidelines. | H |  |  |  |
