# Supplementary material for: Identifying research priorities in breast cancer surgery: a UK priority setting partnership with the James Lind Alliance
Source: Breast Cancer Res Treat. 2022 Nov 1;197(1):39–49. doi: 10.1007/s10549-022-06756-4 (PMC9628302; doi:10.1007/s10549-022-06756-4)
Supplement: Supplementary file 3 — Supplementary file3 (DOCX 140 KB) [file 10549_2022_6756_MOESM3_ESM.docx]

**Appendix 3: Results of Evidence Check for Breast Cancer Surgery JLA PSP**

| **Number** | **Summary question** | **Details of currently available high-quality evidence**  Published in the last 5 years (2016 onwards), in English, systematic review/metanalysis  Databases searched using a targeted title/abstract search and variety of search terms to reflect the concepts or specifics of each individual indicative question (e.g. “systematic review”[Title/Abstract] OR “meta-analysis” ”[Title/Abstract] AND “breast reconstruction”[Title/Abstract] AND “oncolo*”[Title/Abstract]’)  Worldwide:   - The Cochrane Library - PubMed - Early Breast Cancer Clinical Trialists’ Collaborative Group (EBCTCG)   UK guidelines:   - National Institute for Health and Care Excellence (NICE) guidelines - Association of Breast Surgery (ABS) guidelines - British Association of Plastic, Reconstructive and Aesthetic Surgeons (BAPRAS) guidelines | **Outcome** |
| --- | --- | --- | --- |
|  |  |  | **Not research question = not carried forward to prioritisation** |
|  |  |  | **Answered = Red**  **Not carried forward for prioritisation** |
|  |  |  | **Partially answered = Amber**  **Carry forward for prioritisation** |
|  |  |  | **Unanswered = Green**  **Carry forward for prioritisation** |
|  |  |  | **Merged with another question = blue** |
| ***A Questions about diagnosis; improving the diagnostic pathway and information and support for patients diagnosed with breast cancer*** | | | |
| **1** | How well do different breast symptoms (e.g. breast pain) predict for an eventual breast cancer diagnosis and what is the best way of investigating different symptoms to allow reassurance of the patient? | Martín-Díaz M, Maes-Carballo M, Khan KS, Bueno-Cavanillas A. To image or not in noncyclic breast pain? A systematic review. Curr Opin Obstet Gynecol. 2017 Dec;29(6):404-412. doi: 10.1097/GCO.0000000000000407. PMID: 28961632. | There is a low prevalence of breast cancer in patients with painful breast with negative physical examination, and very little research to inform about the effect of performing or avoiding initial imaging test on outcomes of interest. With such limited evidence, only a weak recommendation to reinforce shared decision making about what should be done in the primary care setting can be made, with the backup of a specialized breast unit.  **This is an evidence uncertainty** |
| **2** | What is the impact of a breast cancer diagnosis on patient’s wellbeing, how much information and psychological support do patients want around the time of diagnosis, and what are the best methods to provide and individualise information and support/counselling in the short and longer term? (including merged Q 5,6,7 | At diagnosis:  Brown T, Cruickshank S, Noblet M. Specialist breast care nurses for support of women with breast cancer. Cochrane Database of Systematic Reviews 2021, Issue 2. Art. No.: CD005634. DOI: 10.1002/14651858.CD005634.pub3. Accessed 22 December 2021.  McCaughan E, Parahoo K, Hueter I, Northouse L, Bradbury I. Online support groups for women with breast cancer. Cochrane Database of Systematic Reviews 2017, Issue 3. Art. No.: CD011652. DOI: 10.1002/14651858.CD011652.pub2. Accessed 22 December 2021.  Cramer H, Lauche R, Klose P, Lange S, Langhorst J, Dobos GJ. Yoga for improving health‐related quality of life, mental health and cancer‐related symptoms in women diagnosed with breast cancer. Cochrane Database of Systematic Reviews 2017, Issue 1. Art. No.: CD010802. DOI: 10.1002/14651858.CD010802.pub2. Accessed 22 December 2021.  Ream E, Hughes AE, Cox A, Skarparis K, Richardson A, Pedersen VH, Wiseman T, Forbes A, Bryant A. Telephone interventions for symptom management in adults with cancer. Cochrane Database of Systematic Reviews 2020, Issue 6. Art. No.: CD007568. DOI: 10.1002/14651858.CD007568.pub2. Accessed 22 December 2021.  Schouten B, Avau B, Bekkering GTRUDYE, Vankrunkelsven P, Mebis J, Hellings J, Van Hecke A. Systematic screening and assessment of psychosocial well‐being and care needs of people with cancer. Cochrane Database of Systematic Reviews 2019, Issue 3. Art. No.: CD012387. DOI: 10.1002/14651858.CD012387.pub2. Accessed 22 December 2021.  ***Patients’ experience of a breast cancer diagnosis:***  Brandão T, Schulz MS, Matos PM. Psychological adjustment after breast cancer: a systematic review of longitudinal studies. Psychooncology. 2017 Jul;26(7):917-926. doi: 10.1002/pon.4230. Epub 2016 Aug 12. PMID: 27440317.  Nathoo D, Willis S, Tran WT. Distress Among Locally Advanced Breast Cancer Patients from Diagnosis to Follow-Up: A Critical Review of Literature. J Med Imaging Radiat Sci. 2018 Sep;49(3):325-336. doi: 10.1016/j.jmir.2018.04.034. Epub 2018 Jun 18. PMID: 32074060.  Campbell-Enns HJ, Woodgate RL. The psychosocial experiences of women with breast cancer across the lifespan: a systematic review. Psychooncology. 2017 Nov;26(11):1711-1721. doi: 10.1002/pon.4281. Epub 2016 Oct 4. PMID: 27648726.  Angarita FA, Zhang Y, Elmi M, Look Hong NJ. Older women's experience with breast cancer treatment: A systematic review of qualitative literature. Breast. 2020 Dec;54:293-302. doi: 10.1016/j.breast.2020.11.009. Epub 2020 Nov 16. PMID: 33242756; PMCID: PMC7695983. | Psychosocial interventions delivered by breast care nurse may improve or are at least as effective as standard care and other supportive interventions, during diagnosis, treatment and survivorship.  No evidence that online support is beneficial  Moderate‐quality evidence supports the recommendation of yoga as a supportive intervention for improving health‐related quality of life and reducing fatigue and sleep disturbances when compared with no therapy, as well as for reducing depression, anxiety and fatigue, when compared with psychosocial/educational interventions. Very low‐quality evidence suggests that yoga might be as effective as other exercise interventions and might be used as an alternative to other exercise programmes.  Conclusions related to symptoms most amenable to management by telephone‐delivered interventions are tentative.  Low‐certainty evidence that does not support the effectiveness of screening of psychosocial well‐being and care needs in people with cancer. Need more methodologically robust studies  **This is an evidence uncertainty** |
| **3** | How can we improve the efficiency and timeliness of gathering the information needed to decide optimum treatments (size, receptor and grade status results) for breast cancer? |  | **Access to care/service provision question not a research uncertainty**  **EXCLUDE** |
| **4** | Are there some patients with breast cancer who don’t require investigations (e.g. imaging) or surgery (e.g. sentinel node biopsy) to their axilla (armpit) to determine if their breast cancer has spread? | Question is about targeted axillary investigation/staging  Current RCR guidelines endorsed by ABS state:  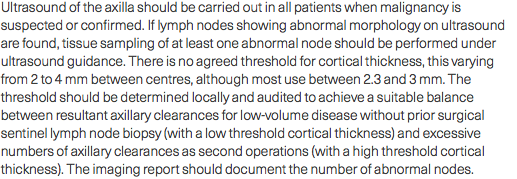  <https://associationofbreastsurgery.org.uk/media/251901/guidance-on-screening-and-symptomatic-breast-imaging-4th-edition.pdf>  Brackstone M, Baldassarre FG, Perera FE, Cil T, Chavez Mac Gregor M, Dayes IS, Engel J, Horton JK, King TA, Kornecki A, George R, SenGupta SK, Spears PA, Eisen AF. Management of the Axilla in Early-Stage Breast Cancer: Ontario Health (Cancer Care Ontario) and ASCO Guideline. J Clin Oncol. 2021 Sep 20;39(27):3056-3082. doi: 10.1200/JCO.21.00934. Epub 2021 Jul 19. PMID: 34279999. | ABS endorsed guidance buts not referenced so not possible to assess quality of evidence  ASCO Guidance (USA)  Gives evidence as to when SLNB can be avoided but not imaging |
| **5** | What are patients’ experiences of a breast cancer diagnosis; what impact does it have on their well-being and how could this be improved? |  | Merged with Q2: What is the impact of a breast cancer diagnosis on patient’s wellbeing, how much information and psychological support do patients want around the time of diagnosis, and what are the best methods to provide and individualise information and support/counselling in the short and longer term? |
| **6** | What information and psychological support do patients want when they are diagnosed with breast cancer and what is the best way to provide and individualise this to aid decision-making? |  | Merged with Q2: What is the impact of a breast cancer diagnosis on patient’s wellbeing, how much information and psychological support do patients want around the time of diagnosis, and what are the best methods to provide and individualise information and support/counselling in the short and longer term? |
| **7** | What are the best methods for counselling patients diagnosed with breast cancer; when should counselling be offered and does it improve long-term outcomes for patients with breast cancer? |  | Merged with Q2: What is the impact of a breast cancer diagnosis on patient’s wellbeing, how much information and psychological support do patients want around the time of diagnosis, and what are the best methods to provide and individualise information and support/counselling in the short and longer term? |
| **8** | How can we improve men’s experience of a breast cancer diagnosis; support them during and after treatment and improve their short and long-term outcomes? | Lin AP, Huang TW, Tam KW. Treatment of male breast cancer: meta-analysis of real-world evidence. Br J Surg. 2021 Sep 27;108(9):1034-1042. doi: 10.1093/bjs/znab279. PMID: 34476472.  Sauder CAM, Bateni SB, Davidson AJ, Nishijima DK. Breast Conserving Surgery Compared With Mastectomy in Male Breast Cancer: A Brief Systematic Review. Clin Breast Cancer. 2020 Jun;20(3):e309-e314. doi: 10.1016/j.clbc.2019.12.004. Epub 2019 Dec 19. PMID: 32171701.  No Cochrane/NICE/ABS guidance | Identification and false-negative rates for SLNB were comparable to those in female breast cancer. Breast-conserving surgery can be effective and safe; postmastectomy radiation to the chest wall and 5-year tamoxifen treatment improves survival.  No differences in DFS, DSS, or OS for BCS and mastectomy. BCS is a reasonable treatment approach for MBC because it was associated with oncologic outcomes similar to those with mastectomy. However, the low rates of radiotherapy compliance among male patients who underwent BCS is concerning |
| ***B Questions on neoadjuvant therapy and treatment sequencing*** | | | |
| **9** | What is the best order of breast cancer treatments (surgery, chemotherapy, radiotherapy and endocrine therapy) and how can we tailor treatment decisions for individual patients (e.g. those having immediate breast reconstruction)? | NICE 2018 evidence review – no difference in overall survival (high quality evidence) for neoadjuvant chemotherapy vs adjuvant. Low quality evidence that patients receiving NACT are more likely to undergo breast conserving surgery.  NICE guidance recommends offering neoadjuvant chemotherapy or ER- or HER2+ breast cancer (in line with other guidelines e.g. St Gallen, which recommend neoadjuvant systemic therapy for HER2+ or TNBC) (Burstein et al Ann Oncol 2021)  EBCTCG meta-analysis (EBCTCG 2018) suggests no significant difference for distant recurrence, breast cancer mortality or death from any cause for neoadjuvant versus adjuvant chemotherapy.  Cochrane review (2013, Hickey et al) suggests that sequencing of chemotherapy and radiotherapy in the adjuvant setting does not influence outcomes. | The first question is therefore not an evidence uncertainty  The question around tailoring treatment at an individual level has not been answered other than at a tumour level (i.e. TNBC or HER2+).  **PROPOSED REVISED QUESTION:**  **How can we tailor decisions about treatment sequencing (surgery; chemotherapy and/or radiotherapy) for individual patients?** |
| **10** | Can we predict which patients with hormone sensitive breast cancer should have chemotherapy and which should have hormone (endocrine) therapy prior to surgery? | NICE 2018 evidence review – comparison of neoadjuvant endocrine therapy (NET) vs neoadjuvant chemotherapy: no evidence for disease-free survival; low quality evidence that there is no clinically meaningful difference in clinical or radiological response of overall survival.  Cochrane review (2014) compares NET with surgery only | This is an evidence uncertainty |
| **11** | If patients with hormone sensitive breast cancer are having treatment before surgery, how can we know who should have chemotherapy and who should have hormone (endocrine) treatment? |  | Merge with question 9  **PROPOSED REVISED QUESTION:**  **How can we tailor decisions about treatment sequencing (surgery; chemotherapy and/or radiotherapy) for individual patients** |
| **12** | Can we predict which patients with breast cancer who need a mastectomy at diagnosis, will be able to have breast conserving surgery if they have chemotherapy before surgery? | NICE guidance (2018) recommends offering neoadjuvant chemotherapy to people with ER- or HER2+ breast cancer as an option to reduce tumour size. Neoadjuvant chemotherapy for ER+ disease to reduce tumour size is recommended if chemotherapy is indicated.  Individual trial data suggests that receptor status and achieving a pathological complete response are predictors of surgical downstaging but there are no recommendations in NICE, nor any meta-analyses on this topic. | This is an evidence uncertainty |
| **13** | Can we predict survival rates, benefits and risk of recurrence associated with different treatment types (for example different types of surgery, or drug treatments given before and/or after surgery)? | Meta-analysis data (Spring Clin Cancer Research 2020) estimates the event free and overall survival benefits resulting from a complete pathological response to treatment according to subtype. EBCTCG quantifies survival and recurrence risk benefits for neoadjuvant versus adjuvant chemotherapy  A NICE evidence review (2018) found no evidence in respect of disease free survival, breast conservation rates or changes in tumour size when comparing neoadjuvant endocrine therapy with no NET. This is the subject of an ongoing clinical trial (EndoNET).  For different types of surgery – there is RCT data to support the equivalence of conservation surgery with mastectomy (Fisher NEJM 2002, Veronesi NEJM 2002). There is more recent large series data to suggest that there may be a survival benefit for BCS + radiotherapy versus mastectomy. However, there is no current NICE guidance, nor Cochrane or EBCTCG data to answer this question. | Partially answered for chemotherapy  Very old data for surgery (BCS+RT vs mastectomy) |
| **14** | How can we best support patients with breast cancer having neoadjuvant chemotherapy? | No recommendations in NICE guidelines; no meta-analyses in Cochrane or EBCTCG. | This is an evidence uncertainty  **Merged with Q2 on support** What is the impact of a breast cancer diagnosis on patient’s wellbeing, how much information and psychological support do patients want around the time of diagnosis, and what are the best methods to provide and individualise information and support/counselling in the short and longer term |
| **15** | What is the best order of breast cancer treatments (surgery, chemotherapy and radiotherapy) in women having immediate breast reconstruction to get the best outcomes and reduce the risk of complications? |  | Merge with question 9  **PROPOSED REVISED QUESTION:**  **How can we tailor decisions about treatment sequencing (surgery; chemotherapy and/or radiotherapy) for individual patients** |
| **16** | What are the benefits of using treatments before surgery (neoadjuvant), for example drugs that target cancer receptors such as oestrogen and HER2 (e.g. does it improve survival or quality of life)? | EBCTCG meta-analysis confirms no survival difference for neoadjuvant versus adjuvant treatment but a slight increase in local recurrence rates and an increase in breast conservation rates.  Other meta-analyses (Spring 2020, Cortazar 2014) confirm that at an individual level achieving a pathological complete response following neoadjuvant chemotherapy or anti-HER2 therapy is highly prognostic for patients. | The clinical/oncological benefits are an answered question  There is no data on quality of life outcomes for neoadjuvant versus adjuvant therapy  **PROPOSED REVISED QUESTION**  **What impact does using treatments before surgery (neoadjuvant) for example drugs that target cancer receptors such as oestrogen and HER2 have on patients’ quality of life and well-being?** |
| **17** | Can the use of short-course neoadjuvant treatment (before surgery) predict the long-term outcomes of breast cancer and identify patients at high risk of breast cancer recurrence? | No recommendations in NICE guidelines. No EBCTCG or Cochrane data.  Robertson et al (2018, Lancet Oncology) confirms that short-course neoadjuvant treatment can predict outcome in high-risk ER+ patients. Bundred et al (Clin Cancer Research, in press) suggests that this is also the case in HER2 positive disease. | **This is an evidence uncertainty** |
| **18** | Are there benefits to commencing patients of treatments that target cancer receptors such as oestrogen and HER2 before surgery? |  | Merged with Q16  **PROPOSED REVISED QUESTION**  **What impact does using treatments before surgery (neoadjuvant) for example drugs that target cancer receptors such as oestrogen and HER2 have on patients’ quality of life and well-being?** |
| **19** | If breast cancer hormone receptors are not assessed prior to neoadjuvant chemotherapy, does it alter patient lifespan? | NICE (and other) guidelines recommend neoadjuvant chemotherapy for triple negative breast cancer (TNBC) or HER2+ disease – therefore it can be inferred that not assessing hormone receptors may impact outcomes such as lifespan. | **This is an answered question** |
| **20** | What is the optimal duration of treatment with neoadjuvant (before surgery) endocrine therapy and when should breast cancer surgery be performed for these patients? | No recommendations in NICE guidelines; no data in EBCTCG or Cochrane  Some data at individual trial level and the subject of ongoing investigation | **This is (currently) an unanswered question** |
| **21** | Can we predict which patients will respond best to endocrine therapy? |  | Merge with question 9  **PROPOSED REVISED QUESTION:**  **How can we tailor decisions about treatment sequencing (surgery; chemotherapy and/or radiotherapy) for individual patients** |
| **22** | Should radiotherapy be given before breast cancer surgery and in which patients? |  | Merge with question 9  **PROPOSED REVISED QUESTION:**  **How can we tailor decisions about treatment sequencing (surgery; chemotherapy and/or radiotherapy) for individual patients** |
| **23** | What is the most effective way to mark and localise breast cancers and axillary lymph nodes in women having neoadjuvant (before surgery) chemotherapy, so that patients don't need another localisation procedure prior to breast cancer surgery? | No recommendations in NICE guidelines; no data in EBCTCG or Cochrane | **This is an evidence uncertainty** |
| **24** | Can we tell whether a patient’s breast cancer has completely responded to neoadjuvant chemotherapy without performing surgery and how long, if at all, after finishing neoadjuvant chemotherapy should breast surgery be performed? | No recommendations in NICE guidelines; no data in EBCTCG or Cochrane  Currently the subject of multiple ongoing trials | **This is (currently) an evidence uncertainty** |
| **25** | Is surgery needed if a patient’s cancer has responded completely to neoadjuvant chemotherapy? |  | **Merged to Q24 (**re-worded)  In patients having breast chemotherapy before surgery, what is the best way of monitoring the cancer and is it possible to tell whether the cancer has completely responded to treatment without performing an operation? How long, if at all, after finishing chemotherapy should an operation be performed |
| **26** | How long after finishing neoadjuvant chemotherapy should breast surgery be performed? |  | **Merged to Q24** (re-worded)  In patients having breast chemotherapy before surgery, what is the best way of monitoring the cancer and is it possible to tell whether the cancer has completely responded to treatment without performing an operation? How long, if at all, after finishing chemotherapy should an operation be performed |
| **27** | What is the optimum way of monitoring breast cancer during neoadjuvant chemotherapy? | No recommendations in NICE guidelines; no data in EBCTCG or Cochrane | **This is an evidence uncertainty**  **Merge with Q24 – reworded to include monitoring:**  In patients having breast chemotherapy before surgery, what is the best way of monitoring the cancer and is it possible to tell whether the cancer has completely responded to treatment without performing an operation? How long, if at all, after finishing chemotherapy should an operation be performed? |
| **28** | What is the best way to treat the axillary lymph nodes in women with breast cancer, who have been treated with chemotherapy before surgery? | No recommendations in NICE guidelines; no data in EBCTCG or Cochrane  Swarnkar PK, Tayeh S, Michell MJ, Mokbel K. The Evolving Role of Marked Lymph Node Biopsy (MLNB) and Targeted Axillary Dissection (TAD) after Neoadjuvant Chemotherapy (NACT) for Node-Positive Breast Cancer: Systematic Review and Pooled Analysis. Cancers (Basel). 2021 Mar 26;13(7):1539. doi: 10.3390/cancers13071539. PMID: 33810544; PMCID: PMC8037051. | **This is an evidence uncertainty**  Further research to confirm the oncological safety of this de-escalation approach of axillary surgery is required. MLNB alone and TAD are associated with acceptably low FNRs and represent valid alternatives to cALND in patients with node-positive breast cancer after excellent response to NACT. |
| ***C Questions about breast cancer surgery*** | | | |
| **29** | How can we ensure that patients can make a fully informed choice about their breast cancer surgery options, feel involved in the process, and have sufficient time to make their decisions? | Stacey D, Légaré F, Lewis K, Barry MJ, Bennett CL, Eden KB, Holmes‐Rovner M, Llewellyn‐Thomas H, Lyddiatt A, Thomson R, Trevena L. Decision aids for people facing health treatment or screening decisions. Cochrane Database of Systematic Reviews 2017, Issue 4. Art. No.: CD001431. DOI: 10.1002/14651858.CD001431.pub5. Accessed 22 December 2021  Si J, Guo R, Lu X, Han C, Xue L, Xing D, Chen C. Decision aids on breast conserving surgery for early stage breast cancer patients: a systematic review. BMC Med Inform Decis Mak. 2020 Oct 22;20(1):275. doi: 10.1186/s12911-020-01295-8. PMID: 33092602; PMCID: PMC7583180.  Gu J, Groot G, Boden C, Busch A, Holtslander L, Lim H. Review of Factors Influencing Women's Choice of Mastectomy Versus Breast Conserving Therapy in Early Stage Breast Cancer: A Systematic Review. Clin Breast Cancer. 2018 Aug;18(4):e539-e554. doi: 10.1016/j.clbc.2017.12.013. Epub 2018 Jan 3. PMID: 29396079. | Growing evidence that decision aids may improve values‐congruent choices. Further research is needed on the effects on adherence with the chosen option, cost‐effectiveness, and use with lower literacy populations.  Decision aids on breast conserving surgery made it easier for patient involvement in surgical decision making and improved decision-related outcomes in most early stage breast cancer patients. With more attention, improving procedures, and better interdisciplinary cooperation, more research is necessary for the improvement of decision aids. And we believe decision aids with agreed objective information are needed.  **Currently evidence uncertainty** |
| **30** | What are the short and long-term patient-reported outcomes of simple mastectomy for breast cancer and can these be improved by better methods for aesthetic flat closure in women not undergoing breast reconstruction? | Ng ET, Ang RZ, Tran BX, Ho CS, Zhang Z, Tan W, Bai Y, Zhang M, Tam WW, Ho RC. Comparing Quality of Life in Breast Cancer Patients Who Underwent Mastectomy Versus Breast-Conserving Surgery: A Meta-Analysis. Int J Environ Res Public Health. 2019 Dec 6;16(24):4970. doi: 10.3390/ijerph16244970. PMID: 31817811; PMCID: PMC6950729. | Only 6 studies included: Our meta-analysis suggests that breast-conserving surgery was preferred over mastectomy because breast-conserving surgery leads to better outcomes in body image, future perspectives and less systemic side effects. Larger prospective multicentre studies that assess QoL at multiple time intervals postoperatively and based on individualised treatment will be useful to determine the impact on QoL in the long run. This will enable breast cancer patients to be better informed when deciding treatment options  **This is an evidence uncertainty** |
| **31** | How can we best support patients psychologically after mastectomy for breast cancer? |  | Merged with Q2 (re-worded): What is the impact of a breast cancer diagnosis on patient’s wellbeing, how much information and psychological support do patients want around the time of diagnosis, and what are the best methods to provide and individualise information and support/counselling in the short and longer term? |
| **32** | What are the outcomes of contralateral mastectomy for symmetry in women with breast cancer but with no additional genetic risk, when should this be performed and how can we best support women’s decision-making?  (question re-worded) | Srethbhakdi A, Brennan ME, Hamid G, Flitcroft K. Contralateral prophylactic mastectomy for unilateral breast cancer in women at average risk: Systematic review of patient reported outcomes. Psychooncology. 2020 Jun;29(6):960-973. doi: 10.1002/pon.5379. Epub 2020 Mar 31. PMID: 32201988. | This study focuses on CPM (not specifically for symmetry) - 19 included articles including 6088 women; significant heterogeneity and low quality of included studies.  Unable to identify anything specifically related to surgery for symmetry  **This is an evidence uncertainty** |
| **33** | How do the cancer outcomes (recurrence and survival) of breast conserving surgery and radiotherapy compare with those following mastectomy alone in patients with breast cancer? | Gui Y, Liu X, Chen X, Yang X, Li S, Pan Q, Luo X, Chen L. A Network Meta-Analysis of Surgical Treatment in Patients With Early Breast Cancer. J Natl Cancer Inst. 2019 Sep 1;111(9):903-915. doi: 10.1093/jnci/djz105. Erratum in: J Natl Cancer Inst. 2020 Jan 1;112(1):117. PMID: 31187142; PMCID: PMC6748720.  RCT data to support the equivalence of conservation surgery with mastectomy (Fisher NEJM 2002, Veronesi NEJM 2002) but this is old and unlikely to reflect current practice. More recent large series data to suggest that there may be a survival benefit for BCS + radiotherapy versus mastectomy. However, there is no current NICE guidance, nor Cochrane or EBCTCG data to answer this question. | Included management of the axilla and radiotherapy so results difficult to interpret  **This is an evidence uncertainty** |
| **34** | How is decision-making for breast cancer surgery affected if women have had cosmetic breast implants? |  | **Merged with question 29:** How can we ensure that patients can make a fully informed choice about their breast cancer surgery options, feel involved in the process, and have sufficient time to make their decisions? |
| **35** | Is minimally invasive, image-guided excision a safe and effective alternative to surgery in patients with breast cancer? | Peek MCL, Ahmed M, Napoli A, Usiskin S, Baker R, Douek M. Minimally invasive ablative techniques in the treatment of breast cancer: a systematic review and meta-analysis. Int J Hyperthermia. 2017 Mar;33(2):191-202. doi: 10.1080/02656736.2016.1230232. Epub 2016 Oct 2. PMID: 27575566.  Xia LY, Hu QL, Xu WY. Efficacy and Safety of Radiofrequency Ablation for Breast Cancer Smaller Than 2 cm: A Systematic Review and Meta-Analysis. Front Oncol. 2021 May 3;11:651646. doi: 10.3389/fonc.2021.651646. PMID: 34012918; PMCID: PMC8126716.  van de Voort EMF, Struik GM, Birnie E, Moelker A, Verhoef C, Klem TMAL. Thermal Ablation as an Alternative for Surgical Resection of Small (≤ 2 cm) Breast Cancers: A Meta-Analysis. Clin Breast Cancer. 2021 Dec;21(6):e715-e730. doi: 10.1016/j.clbc.2021.03.004. Epub 2021 Mar 17. PMID: 33840627. | Minimally invasive ablative techniques are able to successfully induce coagulative necrosis in breast cancer with a low side effect profile. Adequately powered and prospectively conducted cohort trials are required to confirm complete pathological ablation in all patients.  The results show that RFA for breast cancer smaller than 2 cm is safe and effective. However, prospective studies are needed to validate this conclusion.  Thermal ablation techniques treating early-stage breast cancer (≤ 2 cm) are safe and effective based on complete ablation rate and short-term local recurrence rates. Especially, RFA, microwave ablation, and cryoablation are promising techniques as an alternative to surgical resection without jeopardizing current treatment effectiveness or safety. Owing to great heterogeneity in the included studies, a formal recommendation on the best technique is not possible. These findings warrant the design of large randomized controlled trials comparing thermal ablation and breast-conserving surgery in the treatment of T1 breast cancer.  **Currently an evidence uncertainty** (SMALL ongoing) |
| **36** | Are there some low risk breast cancers or screen detected lesions that do not need any treatment at all? | Forester ND, Lowes S, Mitchell E, Twiddy M. High risk (B3) breast lesions: What is the incidence of malignancy for individual lesion subtypes? A systematic review and meta-analysis. Eur J Surg Oncol. 2019 Apr;45(4):519-527. doi: 10.1016/j.ejso.2018.12.008. Epub 2018 Dec 11. PMID: 30579653. | This comprehensive, inclusive assessment of all published literature, provides an accurate estimate of malignancy risk in subgroups of B3 lesions, to guide tailored management strategies. Some lesions have a high risk of malignancy, while others have a much lower risk, and could be safely managed with surveillance strategies rather than surgery.  **Further work is needed – this is an evidence uncertainty** |
| **37** | What is the best management of ductal carcinoma in situ and how is this influenced by tumour and patient characteristics (e.g. patient age)? | Surgery  NICE 2018: Research recommendation - Surgery to the breast:- What is the optimum tumour-free margin width after breast-conserving surgery for women with ductal carcinoma in situ (DCIS) and invasive breast cancer?  Radiotherapy  Goodwin A, Parker S, Ghersi D, Wilcken N. Post-operative radiotherapy for ductal carcinoma in situ of the breast. Cochrane Database of Systematic Reviews 2013, Issue 11. Art. No.: CD000563. DOI: 10.1002/14651858.CD000563.pub7  Tamoxifen  Staley H, McCallum I, Bruce J. Postoperative tamoxifen for ductal carcinoma in situ. Cochrane Database of Systematic Reviews 2012, Issue 10. Art. No.: CD007847. DOI: 10.1002/14651858.CD007847.pub2  Overall  Bouskill K, Hempel S, Richardson A, Ganz PA, Baxi S, Zutshi R, Larkin J, Motala A, Miles JNV, Crandall CJ. Evidence map of ductal carcinoma in situ management options  Menopause . 2019 Nov;26(11):1250-1258. doi: 10.1097/GME.0000000000001397. | The is uncertainty regarding surgical margins (and need for surgical management for LG DCIS)  This review confirms the benefit of adding radiotherapy to breast conserving surgery for the treatment of all women diagnosed with DCIS. No long-term toxicity from use of radiotherapy was identified.  While tamoxifen after local excision for DCIS (with or without adjuvant radiotherapy) reduced the risk of recurrent DCIS (in the ipsi- and contralateral breast), it did not reduce the risk of overall mortality.  **Included 40 systematic reviews demonstrated several areas of uncertainty** |
| **38** | How can we better predict margin involvement at the time of breast conserving surgery to reduce rates of re-excision? | Gray RJ, Pockaj BA, Garvey E, Blair S. Intraoperative Margin Management in Breast-Conserving Surgery: A Systematic Review of the Literature. Ann Surg Oncol. 2018 Jan;25(1):18-27. doi: 10.1245/s10434-016-5756-4. Epub 2017 Jan 5. PMID: 28058560.  St John ER, Al-Khudairi R, Ashrafian H, Athanasiou T, Takats Z, Hadjiminas DJ, Darzi A, Leff DR. Diagnostic Accuracy of Intraoperative Techniques for Margin Assessment in Breast Cancer Surgery: A Meta-analysis. Ann Surg. 2017 Feb;265(2):300-310. doi: 10.1097/SLA.0000000000001897. PMID: 27429028. | A systematic review of the literature showed evidence that several intraoperative techniques and actions can lower the rates of positive margins.  Pooled data suggest that frozen section and cytology have the greatest diagnostic accuracy. However, these methods are resource intensive and turnaround times for results have prevented widespread international adoption. Emerging technologies need to compete with the diagnostic accuracy of existing techniques while offering advantages in terms of speed, cost, and reliability.  **Partially answered** in that there are techniques that reduce rates of margin positivity but more efficient methods are required and need to be evaluated |
| **39** | Can we safely avoid mastectomy in patients with multifocal or multicentric breast cancer? | Winters ZE, Horsnell J, Elvers KT, Maxwell AJ, Jones LJ, Shaaban AM, Schmid P, Williams NR, Beswick A, Greenwood R, Ingram JC, Saunders C, Vaidya JS, Esserman L, Jatoi I, Brunt AM. Systematic review of the impact of breast-conserving surgery on cancer outcomes of multiple ipsilateral breast cancers. BJS Open. 2018 May 22;2(4):162-174. doi: 10.1002/bjs5.53. PMID: 30079385; PMCID: PMC6069349. | The available studies were mainly of moderate quality, historical and underpowered, with limited follow-up and biased case selection favouring BCS rather than mastectomy for low-risk patients. The evidence was inconclusive, weakening support for the St Gallen consensus and supporting a future randomized trial.  **This is an uncertainty** |
| **40** | What is the best surgical management of rarer types of breast cancer? | No evidence identified | **This is an uncertainty** |
| **41** | Can we avoid axillary clearance in women with node positive breast cancer and what are the outcomes or alternatives to axillary node clearance such as targeted axillary node dissection? | Sangha MS, Baker R, Ahmed M. Axillary dissection (ALND) versus axillary observation for low risk, clinically node-negative invasive breast cancer: a systematic review and meta-analysis. Breast Cancer. 2021 Nov;28(6):1212-1224. doi: 10.1007/s12282-021-01273-6. Epub 2021 Jul 9. PMID: 34241800; PMCID: PMC8514376.  Unable to find any evidence regarding TAD in patients hot having NACT | 9 studies identified: Meta-analyses found no significant difference in OS at 5, 10 and 25-years follow-up (5-year ln HR = 0.08, 95% CI - 0.09, 0.25, 10-year ln HR = 0.33, 95% CI - 0.07, 0.72, 25-year ln HR = 0.00, 95% CI - 0.18, 0.19).  ALND does not cause a significant improvement in OS in women with clinically node-negative breast cancer. ALND may improve DFS in the short term by tailoring a proportion of patients towards chemotherapy. Our evidence suggests that when the administration of systemic therapy is balanced between the two arms, axillary de-escalation studies will likely find no difference in OS or DFS.  **This is an uncertainty** |
| **42** | Why has intraoperative radiotherapy (IORT) not been widely adopted in the UK? |  | **This is not a research question – this is a question about implementation of evidence**  **EXCLUDE** |
| **43** | How soon after diagnosis should surgery for breast cancer be performed and what impact do delays in surgical treatment have on long-term breast cancer outcomes? | Su Y, Zheng X, Ouyang Z. The Relationship between Time to Surgery (TTS) and Survival in Breast Cancer: A Systematic Review and Meta-Analysis. Iran J Public Health. 2021 Sep;50(9):1773-1782. doi: 10.18502/ijph.v50i9.7048. PMID: 34722372; PMCID: PMC8542807.  Hanna TP, King WD, Thibodeau S, Jalink M, Paulin GA, Harvey-Jones E, O'Sullivan DE, Booth CM, Sullivan R, Aggarwal A. Mortality due to cancer treatment delay: systematic review and meta-analysis. BMJ. 2020 Nov 4;371:m4087. doi: 10.1136/bmj.m4087. PMID: 33148535; PMCID: PMC7610021. | This meta-analysis showed a significant adverse association between more prolonged time to surgery (TTS) and lower overall survival in patients with breast cancer. It is reasonable to minimize that interval between diagnosis and curative surgery.  Surgery findings were consistent, with a mortality risk for each four week delay of 1.06-1.08 (eg, colectomy 1.06, 95% confidence interval 1.01 to 1.12; breast surgery 1.08, 1.03 to 1.13). |
| **44** | Are there benefits to performing breast cancer surgery at specific phases in the menstrual cycle? | Samuel M, Khin LW, Brennan VK, Yong WS. Timing of breast surgery in premenopausal breast cancer patients. Cochrane Database of Systematic Reviews 2011, Issue 5. Art. No.: CD003720. DOI: 10.1002/14651858.CD003720.pub2. | In the absence of RCTs, this review provides evidence from large prospective observational studies that timing of surgery does not show a significant effect on survival. |
| **45** | How soon after radiotherapy should further surgery to improve the appearance of the breast be performed? | No evidence identified | **This is an uncertainty** |
| **46** | Can molecular tests be used to tailor local therapies for breast cancer? | No evidence identified | **This is an uncertainty**  Merge with question 9 about tailoring treatment  **PROPOSED REVISED QUESTION:**  **How can we tailor decisions about treatment sequencing (surgery; chemotherapy and/or radiotherapy) for individual patients** |
| ***D Questions about oncoplastic and reconstructive surgery*** | | | |
| **47** | What impact does mastectomy with and without different types of breast reconstruction (e.g. implant and tissue-based procedures and immediate and delayed procedures) have on short and long-term oncological, clinical, patient-reported and cost-effectiveness outcomes, and how should these be best discussed with patients to help them make an informed decision with realistic expectations of outcomes?  (re-worded question) | ***Mastectomy +/- breast reconstruction***  Shen Z, Sun J, Yu Y, Chiu C, Zhang Z, Zhang Y, Xu J. Oncological safety and complication risks of mastectomy with or without breast reconstruction: A Bayesian analysis. J Plast Reconstr Aesthet Surg. 2021 Feb;74(2):290-299. doi: 10.1016/j.bjps.2020.08.121. Epub 2020 Sep 20. PMID: 33093010.  Zhang P, Li CZ, Wu CT, Jiao GM, Yan F, Zhu HC, Zhang XP. Comparison of immediate breast reconstruction after mastectomy and mastectomy alone for breast cancer: A meta-analysis. Eur J Surg Oncol. 2017 Feb;43(2):285-293. doi: 10.1016/j.ejso.2016.07.006. Epub 2016 Jul 27. PMID: 27503441.  Zehra S, Doyle F, Barry M, Walsh S, Kell MR. Health-related quality of life following breast reconstruction compared to total mastectomy and breast-conserving surgery among breast cancer survivors: a systematic review and meta-analysis. Breast Cancer. 2020 Jul;27(4):534-566. doi: 10.1007/s12282-020-01076-1. Epub 2020 Mar 12. PMID: 32162181.  Cordova LZ, Hunter-Smith DJ, Rozen WM. Patient reported outcome measures (PROMs) following mastectomy with breast reconstruction or without reconstruction: a systematic review. Gland Surg. 2019 Aug;8(4):441-451. doi: 10.21037/gs.2019.07.02. PMID: 31538070; PMCID: PMC6723012.  Padmalatha S, Tsai YT, Ku HC, Wu YL, Yu T, Fang SY, Ko NY. Higher Risk of Depression After Total Mastectomy Versus Breast Reconstruction Among Adult Women With Breast Cancer: A Systematic Review and Metaregression. Clin Breast Cancer. 2021 Oct;21(5):e526-e538. doi: 10.1016/j.clbc.2021.01.003. Epub 2021 Jan 7. PMID: 33541834.  ***Comparing outcomes of different types of breast reconstruction***  Khajuria A, Prokopenko M, Greenfield M, Smith O, Pusic AL, Mosahebi A. A Meta-analysis of Clinical, Patient-Reported Outcomes and Cost of DIEP versus Implant-based Breast Reconstruction. Plast Reconstr Surg Glob Open. 2019 Oct 28;7(10):e2486. doi: 10.1097/GOX.0000000000002486. PMID: 31772906; PMCID: PMC6846300.  Toyserkani NM, Jørgensen MG, Tabatabaeifar S, Damsgaard T, Sørensen JA. Autologous versus implant-based breast reconstruction: A systematic review and meta-analysis of Breast-Q patient-reported outcomes. J Plast Reconstr Aesthet Surg. 2020 Feb;73(2):278-285. doi: 10.1016/j.bjps.2019.09.040. Epub 2019 Oct 2. PMID: 31711862.  Eltahir Y, Krabbe-Timmerman IS, Sadok N, Werker PMN, de Bock GH. Outcome of Quality of Life for Women Undergoing Autologous versus Alloplastic Breast Reconstruction following Mastectomy: A Systematic Review and Meta-Analysis. Plast Reconstr Surg. 2020 May;145(5):1109-1123. doi: 10.1097/PRS.0000000000006720. PMID: 32332522.  Char S, Bloom JA, Erlichman Z, Jonczyk MM, Chatterjee A. A comprehensive literature review of patient-reported outcome measures (PROMs) among common breast reconstruction options: What types of breast reconstruction score well? Breast J. 2021 Apr;27(4):322-329. doi: 10.1111/tbj.14186. Epub 2021 Feb 9. PMID: 33565192.  ***Decisional regret and decision-making***  Flitcroft K, Brennan M, Spillane A. Decisional regret and choice of breast reconstruction following mastectomy for breast cancer: A systematic review. Psychooncology. 2018 Apr;27(4):1110-1120. doi: 10.1002/pon.4585. Epub 2017 Dec 7. PMID: 29143481.  Flitcroft K, Brennan M, Spillane A. Making decisions about breast reconstruction: A systematic review of patient-reported factors influencing choice. Qual Life Res. 2017 Sep;26(9):2287-2319. doi: 10.1007/s11136-017-1555-z. Epub 2017 Apr 10. PMID: 28397191  Sousa H, Castro S, Abreu J, Pereira MG. A systematic review of factors affecting quality of life after postmastectomy breast reconstruction in women with breast cancer. Psychooncology. 2019 Nov;28(11):2107-2118. doi: 10.1002/pon.5206. Epub 2019 Sep 1. PMID: 31418500.  Paraskeva N, Guest E, Lewis-Smith H, Harcourt D. Assessing the effectiveness of interventions to support patient decision making about breast reconstruction: A systematic review. Breast. 2018 Aug;40:97-105. doi: 10.1016/j.breast.2018.04.020. Epub 2018 May 3. PMID: 29730304.  Berlin NL, Tandon VJ, Hawley ST, Hamill JB, MacEachern MP, Lee CN, Wilkins EG. Feasibility and Efficacy of Decision Aids to Improve Decision Making for Postmastectomy Breast Reconstruction: A Systematic Review and Meta-analysis. Med Decis Making. 2019 Jan;39(1):5-20. doi: 10.1177/0272989X18803879. PMID: 30799692.  ***Cost-effectiveness/cost comparisons***  Bouhadana G, Safran T, Al-Halabi B, Davison PG. Use of Decision Analysis and Economic Evaluation in Breast Reconstruction: A Systematic Review. Plast Reconstr Surg Glob Open. 2020 Apr 27;8(4):e2786. doi: 10.1097/GOX.0000000000002786. PMID: 32440446; PMCID: PMC7209866.  Khajuria A, Prokopenko M, Greenfield M, Smith O, Pusic AL, Mosahebi A. A Meta-analysis of Clinical, Patient-Reported Outcomes and Cost of DIEP versus Implant-based Breast Reconstruction. Plast Reconstr Surg Glob Open. 2019 Oct 28;7(10):e2486. doi: 10.1097/GOX.0000000000002486. PMID: 31772906; PMCID: PMC6846300  Sheckter CC, Matros E, Momeni A. Assessing value in breast reconstruction: A systematic review of cost-effectiveness studies. J Plast Reconstr Aesthet Surg. 2018 Mar;71(3):353-365. doi: 10.1016/j.bjps.2017.09.010. Epub 2017 Oct 9. PMID: 29196176. | Oncological safety is addressed  **Uncertainty re: clinical, patient reported and cost-effectiveness outcomes and the best ways to support patients to make a decision** |
| **48** | What are the long-term oncological outcomes of skin and nipple-sparing mastectomy with respect to cancer recurrence, spread and mortality? | Mota BS, Riera R, Ricci MDesidério, Barrett J, de Castria TB, Atallah ÁN, Bevilacqua JB. Nipple- and areola-sparing mastectomy for the treatment of breast cancer. Cochrane Database of Systematic Reviews 2016, Issue 11. Art. No.: CD008932. DOI: 10.1002/14651858.CD008932.pub3  "systematic review"[Title/Abstract] AND "nipple-sparing*"[Title/Abstract] AND "oncolo**"[Title/Abstract]  Agha RA, Al Omran Y, Wellstead G, Sagoo H, Barai I, Rajmohan S, Borrelli MR, Vella-Baldacchino M, Orgill DP, Rusby JE. Systematic review of therapeutic nipple-sparing versus skin-sparing mastectomy. BJS Open. 2018 Dec 19;3(2):135-145. doi: 10.1002/bjs5.50119. PMID: 30957059; PMCID: PMC6433323.  De La Cruz L, Moody AM, Tappy EE, Blankenship SA, Hecht EM. Overall Survival, Disease-Free Survival, Local Recurrence, and Nipple-Areolar Recurrence in the Setting of Nipple-Sparing Mastectomy: A Meta-Analysis and Systematic Review. Ann Surg Oncol. 2015 Oct;22(10):3241-9. doi: 10.1245/s10434-015-4739-1. Epub 2015 Aug 5. PMID: 26242363. | The findings from these observational studies of very low‐quality evidence were inconclusive for all outcomes due to the high risk of selection bias.  **This is an uncertainty** |
| **49** | What is the best way to correctly identify the mastectomy plane to ensure that all breast tissue is removed while maintaining the skin’s blood supply in women having a skin/nipple sparing mastectomy for breast cancer? | Preserving skin blood supply to reduce complications  Pruimboom T, Schols RM, Van Kuijk SMJ, Van der Hulst RRWJ, Qiu SS. Indocyanine green angiography for preventing postoperative mastectomy skin flap necrosis in immediate breast reconstruction. Cochrane Database of Systematic Reviews 2020, Issue 4. Art. No.: CD013280. DOI: 10.1002/14651858.CD013280.pub2 | High-quality randomised controlled studies that compare the use of ICGA to assess MSFN compared to clinical evaluation are needed.  **This is an uncertainty** |
| **50** | Is breast reconstruction safe with respect to cancer outcomes in women with locally advanced breast cancer? | No SR/MA identified | **Merged with Q47** What impact does mastectomy with and without different types of breast reconstruction (e.g. implant and tissue-based procedures and immediate and delayed procedures) have on short and long-term oncological, clinical, patient-reported and cost-effectiveness outcomes, and how should these be best discussed with patients to help them make an informed decision? |
| **52** | How do the oncological, clinical and patient reported outcomes of oncoplastic breast conserving procedures (volume displacement with therapeutic mammaplasty; volume replacement with local flaps) compare with those of wide local excision, mastectomy and mastectomy with immediate or delayed breast reconstruction, how do they differ across patient groups and how can we best support women to make informed decisions about their surgical options? (re-worded) | Nanda A, Hu J, Hodgkinson S, Ali S, Rainsbury R, Roy PG. Oncoplastic breast-conserving surgery for women with primary breast cancer. Cochrane Database of Systematic Reviews 2021, Issue 10. Art. No.: CD013658. DOI: 10.1002/14651858.CD013658.pub2.  Kosasih S, Tayeh S, Mokbel K, Kasem A. Is oncoplastic breast conserving surgery oncologically safe? A meta-analysis of 18,103 patients. Am J Surg. 2020 Aug;220(2):385-392. doi: 10.1016/j.amjsurg.2019.12.019. Epub 2020 Jan 2. PMID: 31926592.  Char S, Bloom JA, Erlichman Z, Jonczyk MM, Chatterjee A. A comprehensive literature review of patient-reported outcome measures (PROMs) among common breast reconstruction options: What types of breast reconstruction score well? Breast J. 2021 Apr;27(4):322-329. doi: 10.1111/tbj.14186. Epub 2021 Feb 9. PMID: 33565192.  Lee A, Kwasnicki RM, Khan H, Grant Y, Chan A, Fanshawe AEE, Leff DR. Outcome reporting in therapeutic mammaplasty: a systematic review. BJS Open. 2021 Nov 9;5(6):zrab126. Doi: 10.1093/bjsopen/zrab126. PMID: 34894122; PMCID: PMC8665419.  Pujji OJS, Blackhall V, Romics L, Vidya R. Systematic review of partial breast reconstruction with pedicled perforator artery flaps: Clinical, oncological and cosmetic outcomes. Eur J Surg Oncol. 2021 Aug;47(8):1883-1890. doi: 10.1016/j.ejso.2021.03.249. Epub 2021 Apr 13. PMID: 33895022.  Rocco N, Catanuto G, Cinquini M, Audretsch W, Benson J, Criscitiello C, Di Micco R, Kovacs T, Kuerer H, Lozza L, Montagna G, Moschetti I, Nafissi N, O'Connell RL, Oliveri S, Pau L, Scaperrotta G, Thoma A, Winters Z, Nava MB. Should oncoplastic breast conserving surgery be used for the treatment of early stage breast cancer? Using the GRADE approach for development of clinical recommendations. Breast. 2021 Jun;57:25-35. doi: 10.1016/j.breast.2021.02.013. Epub 2021 Feb 26. PMID: 33711697; PMCID: PMC7970134. | The evidence is very uncertain regarding oncological outcomes following O-BCS compared to S-BCS, though O-BCS has not been shown to be inferior. O-BCS may result in less need for a second re-excision surgery but may result in more complications and a greater recall rate than S-BCS. It seems that O-BCS may give better patient satisfaction and surgeon rating for the look of the breast, but the evidence for this is of poor quality, and due to lack of numerical data, it was not possible to pool the results of different studies. It seems O-BCS results in fewer complications compared with surgeries involving mastectomy. Based on this review, no certain conclusions can be made to help inform policymakers. The surgical decision for what operation to proceed with should be made jointly between clinician and patient after an appropriate discussion about the risks and benefits of O-BCS personalised to the patient, taking into account clinicopathological factors. This review highlighted the deficiency of well-conducted studies to evaluate efficacy, safety and patient-reported outcomes following O-BCS.  All based on very heterogenous studies; more research is needed  **This is an uncertainty** |
| **53** | What are the clinical and patient-reported outcomes of mastectomy with and without breast reconstruction, and how should these be best discussed with patients to help them make an informed decision |  | **Merged with re-worded Q47**  What impact does mastectomy with and without different types of breast reconstruction (e.g. implant and tissue-based procedures and immediate and delayed procedures) have on short and long-term oncological, clinical, patient-reported and cost-effectiveness outcomes, and how should these be best discussed with patients to help them make an informed decision? |
| **54** | Is simple mastectomy presented as an equally valid alternative to breast reconstruction in women with breast cancer and if not, why is this and how can this information provision be improved? | NICE Guideline NG101: Early and locally advanced breast cancer: diagnosis and management - 1.5.2 Be aware that some women may prefer not to have breast reconstruction surgery. | **Merged with re-worded Q47** What impact does mastectomy with and without different types of breast reconstruction (e.g. implant and tissue-based procedures and immediate and delayed procedures) have on short and long-term oncological, clinical, patient-reported and cost-effectiveness outcomes, and how should these be best discussed with patients to help them make an informed decision? |
| **55** | What is the psychological impact of not being able to have breast reconstruction in women with breast cancer who would like reconstructive surgery and how can these women be best supported? | Padmalatha S, Tsai YT, Ku HC, Wu YL, Yu T, Fang SY, Ko NY. Higher Risk of Depression After Total Mastectomy Versus Breast Reconstruction Among Adult Women With Breast Cancer: A Systematic Review and Metaregression. Clin Breast Cancer. 2021 Oct;21(5):e526-e538. doi: 10.1016/j.clbc.2021.01.003. Epub 2021 Jan 7. PMID: 33541834. | **Merged with re-worded Q47** What impact does mastectomy with and without different types of breast reconstruction (e.g. implant and tissue-based procedures and immediate and delayed procedures) have on short and long-term oncological, clinical, patient-reported and cost-effectiveness outcomes, and how should these be best discussed with patients to help them make an informed decision? |
| **56** | What the clinical and patient-reported outcomes of immediate and delayed breast reconstruction, and how should these outcomes be presented to patients? |  | **Merged with re-worded Q47** What impact does mastectomy with and without different types of breast reconstruction (e.g. implant and tissue-based procedures and immediate and delayed procedures) have on short and long-term oncological, clinical, patient-reported and cost-effectiveness outcomes, and how should these be best discussed with patients to help them make an informed decision? |
| **57** | What are the short and long-term clinical and patient-reported outcomes and cost-effectiveness of different types of breast reconstruction surgery (e.g. implant and tissue-based procedures)? |  | **Merged with re-worded Q47** What impact does mastectomy with and without different types of breast reconstruction (e.g. implant and tissue-based procedures and immediate and delayed procedures) have on short and long-term oncological, clinical, patient-reported and cost-effectiveness outcomes, and how should these be best discussed with patients to help them make an informed decision? |
| **58** | What are the short and long-term outcomes of implant-based breast reconstruction with and without biological and synthetic mesh and how can these be improved? | Whisker et al. Biological and synthetic mesh assisted breast reconstruction procedures: Joint guidelines from the Association of Breast Surgery and the British Association of Plastic, Reconstructive and Aesthetic Surgeons. European Journal of Surgical Oncology  Volume 47, Issue 11, November 2021, Pages 2807-2813  Hallberg H, Rafnsdottir S, Selvaggi G, Strandell A, Samuelsson O, Stadig I, Svanberg T, Hansson E, Lewin R. Benefits and risks with acellular dermal matrix (ADM) and mesh support in immediate breast reconstruction: a systematic review and meta-analysis. J Plast Surg Hand Surg. 2018 Jun;52(3):130-147. doi: 10.1080/2000656X.2017.1419141. Epub 2018 Jan 10. PMID: 29320921.  Chicco M, Huang TC, Cheng HT. Negative-Pressure Wound Therapy in the Prevention and Management of Complications From Prosthetic Breast Reconstruction: A Systematic Review and Meta-analysis. Ann Plast Surg. 2021 Oct 1;87(4):478-483. doi: 10.1097/SAP.0000000000002722. PMID: 34060773. | All reviews and ABS/BAPRAS guidelines highlight need for high-quality evidence to inform practice  **This is an uncertainty** |
| **59** | Does prehabilitation and preoperative exercise and enhanced revery programmes reduce length of stay and improve outcomes for women having breast and reconstructive surgery for breast cancer? | Treanor C, Kyaw T, Donnelly M. An international review and meta-analysis of prehabilitation compared to usual care for cancer patients. J Cancer Surviv. 2018 Feb;12(1):64-73. doi: 10.1007/s11764-017-0645-9. Epub 2017 Sep 12. PMID: 28900822.  Soteropulos CE, Tang SYQ, Poore SO. Enhanced Recovery after Surgery in Breast Reconstruction: A Systematic Review. J Reconstr Microsurg. 2019 Nov;35(9):695-704. doi: 10.1055/s-0039-1693699. Epub 2019 Aug 1. PMID: 31370092.  Temple-Oberle C, Shea-Budgell MA, Tan M, Semple JL, Schrag C, Barreto M, Blondeel P, Hamming J, Dayan J, Ljungqvist O; ERAS Society. Consensus Review of Optimal Perioperative Care in Breast Reconstruction: Enhanced Recovery after Surgery (ERAS) Society Recommendations. Plast Reconstr Surg. 2017 May;139(5):1056e-1071e. doi: 10.1097/PRS.0000000000003242. PMID: 28445352. | **This is an uncertainty** |
| **60** | Do the clinical and patient-reported outcomes of oncoplastic breast conserving differ in women at high risk of complications (e.g. smokers, women with high BMI)? |  | **Merge with Q52:** How do the oncological, clinical and patient reported outcomes of oncoplastic breast conserving procedures (volume displacement with therapeutic mammaplasty; volume replacement with local flaps) compare with those of wide local excision, mastectomy and mastectomy with immediate or delayed breast reconstruction, how do they differ across patient groups and how can we best support women to make informed decisions about their surgical options? |
| **61** | How can we best support patients to make informed decisions about oncoplastic and reconstructive surgery including ensuring that they are offered robust information about all appropriate options and that they have realistic expectations of outcomes? |  | **Merged with Q47 and Q52 (see above)** What impact does mastectomy with and without different types of breast reconstruction (e.g. implant and tissue-based procedures and immediate and delayed procedures) have on short and long-term oncological, clinical, patient-reported and cost-effectiveness outcomes, and how should these be best discussed with patients to help them make an informed decision with realistic expectations of outcomes? |
| **62** | Does 3D scanning improve the outcomes of reconstructive breast surgery? | No evidence | **This is an uncertainty** |
| **63** | Are the outcomes of breast reconstruction different if the procedure is performed by a breast or plastic surgeon? | Not a research question | **Not a research question; related to service provision/delivery of care**  **EXCLUDE** |
| **64** | How can we reduce length of stay following tissue-based (autologous) breast reconstruction? | systematic review"[Title/Abstract] AND "preoper*"[Title/Abstract] AND "breast reconstruction"[Title/Abstract]  Soteropulos CE, Tang SYQ, Poore SO. Enhanced Recovery after Surgery in Breast Reconstruction: A Systematic Review. J Reconstr Microsurg. 2019 Nov;35(9):695-704. doi: 10.1055/s-0039-1693699. Epub 2019 Aug 1. PMID: 31370092.  Temple-Oberle C, Shea-Budgell MA, Tan M, Semple JL, Schrag C, Barreto M, Blondeel P, Hamming J, Dayan J, Ljungqvist O; ERAS Society. Consensus Review of Optimal Perioperative Care in Breast Reconstruction: Enhanced Recovery after Surgery (ERAS) Society Recommendations. Plast Reconstr Surg. 2017 May;139(5):1056e-1071e. doi: 10.1097/PRS.0000000000003242. PMID: 28445352. | **Merge with Q59** about prehabilitation  Does prehabilitation and preoperative exercise and enhanced revery programmes reduce length of stay and improve outcomes for women having breast and reconstructive surgery for breast cancer? |
| **65** | Does measuring patient reported outcomes contribute to the delivery of good quality breast reconstruction surgery; how many units are collecting data and how should this be used? |  | **This is not a research question – service evaluation**  **EXCLUDE** |
| **66** | What are the outcomes of women electing to have a contralateral mastectomy for symmetry after breast cancer surgery and how can we best support women’s decision-making? |  | **Merged to Q32 (re-worded)**  What are the outcomes of contralateral mastectomy for symmetry in women with breast cancer but with no additional genetic risk, when should this be performed and how can we best support women’s decision-making? |
| **67** | When is symmetrisation or balancing surgery required and should it be performed at the same time as the breast cancer surgery or at a later date? | No evidence identified | **This is an uncertainty** |
| **68** | What impact does radiotherapy have on the outcomes of breast reconstruction and how does this vary by the type of reconstruction surgery performed (e.g. implant vs tissue-based procedures)? (question reworded) | NICE Guideline NG101: Early and locally advanced breast cancer: diagnosis and management - Research recommendation: What are the long‑term outcomes for breast reconstruction in women having radiotherapy to the chest wall?  "systematic review"[Title/Abstract] AND "breast reconstruction"[Title/Abstract] AND "radiotherapy*"[Title/Abstract]  Khajuria A, Charles WN, Prokopenko M, Beswick A, Pusic AL, Mosahebi A, Dodwell DJ, Winters ZE. Immediate and delayed autologous abdominal microvascular flap breast reconstruction in patients receiving adjuvant, neoadjuvant or no radiotherapy: a meta-analysis of clinical and quality-of-life outcomes. BJS Open. 2020 Apr;4(2):182-196. doi: 10.1002/bjs5.50245. Epub 2019 Dec 29. PMID: 32207573; PMCID: PMC7093792.  Heiman AJ, Gabbireddy SR, Kotamarti VS, Ricci JA. A Meta-Analysis of Autologous Microsurgical Breast Reconstruction and Timing of Adjuvant Radiation Therapy. J Reconstr Microsurg. 2021 May;37(4):336-345. doi: 10.1055/s-0040-1716846. Epub 2020 Sep 21. PMID: 32957153.  Liew B, Southall C, Kanapathy M, Nikkhah D. Does post-mastectomy radiation therapy worsen outcomes in immediate autologous breast flap reconstruction? A systematic review and meta-analysis. J Plast Reconstr Aesthet Surg. 2021 Dec;74(12):3260-3280. doi: 10.1016/j.bjps.2021.08.005. Epub 2021 Aug 28. PMID: 34565703.  Zugasti A, Hontanilla B. The Impact of Adjuvant Radiotherapy on Immediate Implant-based Breast Reconstruction Surgical and Satisfaction Outcomes: A Systematic Review and Meta-analysis. Plast Reconstr Surg Glob Open. 2021 Nov 5;9(11):e3910. doi: 10.1097/GOX.0000000000003910. PMID: 34765389; PMCID: PMC8575424.  O'Donnell JPM, Murphy D, Ryan ÉJ, Gasior SA, Sugrue R, O'Neill BL, Boland MR, Lowery AJ, Kerin MJ, McInerney NM. Optimal reconstructive strategies in the setting of post-mastectomy radiotherapy - A systematic review and network meta-analysis. Eur J Surg Oncol. 2021 Nov;47(11):2797-2806. doi: 10.1016/j.ejso.2021.07.001. Epub 2021 Jul 9. PMID: 34301444. | Lack of high quality evidence identified in all reviews  Merge all radiotherapy and reconstruction questions:  **PROPOSED REWORDED QUESTION What impact does radiotherapy have on the outcomes of different types of breast reconstruction; what is the best timing of reconstruction if radiotherapy is needed and how can we support women to make informed decisions about surgery and minimise poor outcomes in this group?** |
| **69** | What is the best timing of breast reconstruction if a patient needs radiotherapy, does the need for radiotherapy impact patients’ choices and decision-making for breast reconstruction? | NICE Guideline NG101: Early and locally advanced breast cancer: diagnosis and management - ‘1.5.1 Offer both breast reconstruction options to women (immediate reconstruction and delayed reconstruction), whether or not they are available locally’  ‘1.5.3 Offer immediate breast reconstruction to women who have been advised to have a mastectomy, including those who may need radiotherapy, unless they have significant comorbidities that rule out reconstructive surgery’  Hershenhouse KS, Bick K, Shauly O, Kondra K, Ye J, Gould DJ, Patel KM. "Systematic review and meta-analysis of immediate versus delayed autologous breast reconstruction in the setting of post-mastectomy adjuvant radiation therapy". J Plast Reconstr Aesthet Surg. 2021 May;74(5):931-944. doi: 10.1016/j.bjps.2020.11.027. Epub 2020 Dec 5. PMID: 33423976.  O'Donnell JPM, Murphy D, Ryan ÉJ, Gasior SA, Sugrue R, O'Neill BL, Boland MR, Lowery AJ, Kerin MJ, McInerney NM. Optimal reconstructive strategies in the setting of post-mastectomy radiotherapy - A systematic review and network meta-analysis. Eur J Surg Oncol. 2021 Nov;47(11):2797-2806. doi: 10.1016/j.ejso.2021.07.001. Epub 2021 Jul 9. PMID: 34301444. | Merge all radiotherapy and reconstruction questions into 1 **question Q68 reworded**  **PROPOSED REWORDED QUESTION What impact does radiotherapy have on the outcomes of different types of breast reconstruction; what is the best timing of reconstruction if radiotherapy is needed and how can we support women to make informed decisions about surgery and minimise poor outcomes in this group?** |
| **70** | How do we develop national consensus on reconstruction and radiotherapy? | Combine all RT questions into 1 | Merge all radiotherapy and reconstruction questions into 1 **question Q68 reworded**  **PROPOSED REWORDED QUESTION What impact does radiotherapy have on the outcomes of different types of breast reconstruction; what is the best timing of reconstruction if radiotherapy is needed and how can we support women to make informed decisions about surgery and minimise poor outcomes in this group?** |
| **71** | Can we identify women at risk of poor outcomes following breast reconstruction and radiotherapy and explore ways these outcomes could be improved (e.g. different ways of giving radiotherapy)? | Combine all RT questions into 1 | Merge all radiotherapy and reconstruction questions into 1 **question Q68 reworded**  **PROPOSED REWORDED QUESTION What impact does radiotherapy have on the outcomes of different types of breast reconstruction; what is the best timing of reconstruction if radiotherapy is needed and how can we support women to make informed decisions about surgery and minimise poor outcomes in this group?** |
| **72** | How can we evaluate new techniques and devices for breast cancer surgery safely and effectively? |  | Answered question  EXCLUDE |
| **73** | Is it possible to develop new options for breast reconstruction for women not suitable for implant or flap based reconstruction? |  | **This is an uncertainty** |
| **74** | Is a remote incision (i.e. periareolar) necessary or appropriate with reconstruction? | Not research question | Not a research question  EXCLUDE |
| **75** | Do we need a core outcome set (a minimum group of outcomes that all studies need to report) for breast surgery? | Not a research question – not for inclusion in consensus process | Already answered  Yes |
| ***E Questions about short-term post-operative care (pain relief; wound care; physiotherapy)*** | | | |
| **76** | How can we best practically and psychologically support patients around the time of their breast cancer surgery to ensure they are fully informed about the procedure; potential complications and feel confident before the operation and after discharge? | Merge all support questions | **Merged with Q2:** What is the impact of a breast cancer diagnosis on patient’s wellbeing, how much information and psychological support do patients want around the time of diagnosis, and what are the best methods to provide and individualise information and support/counselling in the short and longer term? |
| **77** | How can we best prepare women for pain following breast surgery; manage this pain and prevent chronic pain and best manage this if it develops?  (reworded to include all pain questions) | McCowat M, Fleming L, Vibholm J, Dixon D. The Psychological Predictors of Acute and Chronic Pain in Women Following Breast Cancer Surgery: A Systematic Review. Clin J Pain. 2019 Mar;35(3):261-271. doi: 10.1097/AJP.0000000000000672. PMID: 30531400.  Rai AS, Khan JS, Dhaliwal J, Busse JW, Choi S, Devereaux PJ, Clarke H. Preoperative pregabalin or gabapentin for acute and chronic postoperative pain among patients undergoing breast cancer surgery: A systematic review and meta-analysis of randomized controlled trials. J Plast Reconstr Aesthet Surg. 2017 Oct;70(10):1317-1328. doi: 10.1016/j.bjps.2017.05.054. Epub 2017 Jun 9. PMID: 28751024.  Klifto KM, Elhelali A, Payne RM, Cooney CM, Manahan MA, Rosson GD. Perioperative systemic nonsteroidal anti‐inflammatory drugs (NSAIDs) in women undergoing breast surgery. Cochrane Database of Systematic Reviews 2021, Issue 11. Art. No.: CD013290. DOI: 10.1002/14651858.CD013290.pub2. Accessed 22 December 2021.  Chhabra A, Roy Chowdhury A, Prabhakar H, Subramaniam R, Arora MKumar, Srivastava A, Kalaivani M. Paravertebral anaesthesia with or without sedation versus general anaesthesia for women undergoing breast cancer surgery. Cochrane Database of Systematic Reviews 2021, Issue 2. Art. No.: CD012968. DOI: 10.1002/14651858.CD012968.pub2. Accessed 22 December 2021.  Moloney NA, Pocovi NC, Dylke ES, Graham PL, De Groef A. Psychological Factors Are Associated with Pain at All Time Frames After Breast Cancer Surgery: A Systematic Review with Meta-Analyses. Pain Med. 2021 Apr 20;22(4):915-947. Doi: 10.1093/pm/pnaa363. PMID: 33547465.  Wang L, Cohen JC, Devasenapathy N, Hong BY, Kheyson S, Lu D, Oparin Y, Kennedy SA, Romerosa B, Arora N, Kwon HY, Jackson K, Prasad M, Jayasekera D, Li A, Guarna G, Natalwalla S, Couban RJ, Reid S, Khan JS, McGillion M, Busse JW. Prevalence and intensity of persistent post-surgical pain following breast cancer surgery: a systematic review and meta-analysis of observational studies. Br J Anaesth. 2020 Sep;125(3):346-357. Doi: 10.1016/j.bja.2020.04.088. Epub 2020 Jun 28. PMID: 32611524.  McCowat M, Fleming L, Vibholm J, Dixon D. The Psychological Predictors of Acute and Chronic Pain in Women Following Breast Cancer Surgery: A Systematic Review. Clin J Pain. 2019 Mar;35(3):261-271. Doi: 10.1097/AJP.0000000000000672. PMID: 30531400.  Leysen L, Beckwée D, Nijs J, Pas R, Bilterys T, Vermeir S, Adriaenssens N. Risk factors of pain in breast cancer survivors: a systematic review and meta-analysis. Support Care Cancer. 2017 Dec;25(12):3607-3643. Doi: 10.1007/s00520-017-3824-3. Epub 2017 Aug 10. PMID: 28799015.  Rai AS, Khan JS, Dhaliwal J, Busse JW, Choi S, Devereaux PJ, Clarke H. Preoperative pregabalin or gabapentin for acute and chronic postoperative pain among patients undergoing breast cancer surgery: A systematic review and meta-analysis of randomized controlled trials. J Plast Reconstr Aesthet Surg. 2017 Oct;70(10):1317-1328. Doi: 10.1016/j.bjps.2017.05.054. Epub 2017 Jun 9. PMID: 28751024.  Johannsen M, Frederiksen Y, Jensen AB, Zachariae R. Psychosocial predictors of posttreatment pain after nonmetastatic breast cancer treatment: a systematic review and meta-analysis of prospective studies. J Pain Res. 2017 Dec 21;11:23-36. doi: 10.2147/JPR.S124665. PMID: 29317846; PMCID: PMC5743183.  Pinheiro da Silva F, Moreira GM, Zomkowski K, Amaral de Noronha M, Flores Sperandio F. Manual Therapy as Treatment for Chronic Musculoskeletal Pain in Female Breast Cancer Survivors: A Systematic Review and Meta-Analysis. J Manipulative Physiol Ther. 2019 Sep;42(7):503-513. Doi: 10.1016/j.jmpt.2018.12.007. PMID: 31864435. | Low‐certainty evidence suggests that NSAIDs may reduce postoperative pain, nausea and vomiting, and postoperative opioid use. NEED RCTs  Moderate‐certainty evidence shows that paravertebral anaesthesia probably reduces PONV, hospital stay, postoperative pain (at 2 hours), and time to ambulation and results in greater patient satisfaction on the first postoperative day compared to general anaesthesia. May also reduce postoperative analgesic use and postoperative pain at 6 and 24 hours at rest and on movement based on low‐certainty evidence. RCTs needed to confirm these results. Adverse events observed with paravertebral anaesthesia are rare.  **This is an uncertainty** |
| **78** | How can rates of day case breast cancer surgery be increased? | No literature found  Perhaps this is more about service provision than research | **Service delivery – not a research question**  **EXCLUDE** |
| **79** | Are dressings needed following breast cancer surgery and if so, which ones should be used and for how long? | Chicco M, Huang TC, Cheng HT. Negative-Pressure Wound Therapy in the Prevention and Management of Complications From Prosthetic Breast Reconstruction: A Systematic Review and Meta-analysis. Ann Plast Surg. 2021 Oct 1;87(4):478-483. doi: 10.1097/SAP.0000000000002722. PMID: 34060773. | More evidence needed  **This is an uncertainty** |
| **80** | How can we minimise short and long-term complications and scarring following breast cancer surgery? (reworded) | Gallagher M, Jones DJ, Bell‐Syer SV. Prophylactic antibiotics to prevent surgical site infection after breast cancer surgery. Cochrane Database of Systematic Reviews 2019, Issue 9. Art. No.: CD005360. DOI: 10.1002/14651858.CD005360.pub5. Accessed 22 December 2021. | Probably reduce SSI but not enough evidence  **Merge with questions 89 and 90** on cording and other complication question |
| **81** | What are the risk factors for post-operative seroma; how can we prevent them from developing and how should they best be managed? | De Rooij L, Bosmans JWAM, van Kuijk SMJ, Vissers YLJ, Beets GL, van Bastelaar J. A systematic review of seroma formation following drain-free mastectomy. Eur J Surg Oncol. 2021 Apr;47(4):757-763. doi: 10.1016/j.ejso.2020.10.010. Epub 2020 Oct 10. PMID: 33051116.  Velotti N, Limite G, Vitiello A, Berardi G, Musella M. Flap fixation in preventing seroma formation after mastectomy: an updated meta-analysis. Updates Surg. 2021 Aug;73(4):1307-1314. doi: 10.1007/s13304-021-01049-9. Epub 2021 Apr 22. PMID: 33886107; PMCID: PMC8397649. | More evidence needed – merge drain and seroma question  **Re-worded Q:**  **What are the risk factors for post-operative seroma; how can we prevent them from developing? Are drains needed to manage them and if so, when?** |
| **82** | Do we need to use drains following breast cancer surgery, if so, when, what type and how should they best be managed? | De Rooij L, Bosmans JWAM, van Kuijk SMJ, Vissers YLJ, Beets GL, van Bastelaar J. A systematic review of seroma formation following drain-free mastectomy. Eur J Surg Oncol. 2021 Apr;47(4):757-763. doi: 10.1016/j.ejso.2020.10.010. Epub 2020 Oct 10. PMID: 33051116.  Shima H, Kutomi G, Sato K, Kuga Y, Wada A, Satomi F, Uno S, Nisikawa N, Kameshima H, Ohmura T, Mizuguchi T, Takemasa I. An Optimal Timing for Removing a Drain After Breast Surgery: A Systematic Review and Meta-Analysis. J Surg Res. 2021 Nov;267:267-273. Doi: 10.1016/j.jss.2021.05.031. Epub 2021 Jun 23. PMID: 34171562.  Scomacao I, Cummins A, Roan E, Duraes EFR, Djohan R. The use of surgical site drains in breast reconstruction: A systematic review. J Plast Reconstr Aesthet Surg. 2020 Apr;73(4):651-662. doi: 10.1016/j.bjps.2019.11.019. Epub 2019 Dec 4. PMID: 31926896.  Cochrane review is 2013  Thomson DR, Sadideen H, Furniss D. Wound drainage after axillary dissection for carcinoma of the breast. Cochrane Database of Systematic Reviews 2013, Issue 10. Art. No.: CD006823. DOI: 10.1002/14651858.CD006823.pub2. | **Merge with Q68 reworded**  **What are the risk factors for post-operative seroma; how can we prevent them from developing? Are drains needed to manage them and if so, when?**  data heterogeneous with seroma being poorly and inconsistently defined. Concluded that despite the data difficulties, drainage after mastectomy and axillary surgery could be safely omitted, but that the importance of flap fixation was not yet well studied.  Seroma formation was significantly higher in patients who had early drain removal. Conversely, SSI incidence was low, and early removal did not increase SSI incidence. In conclusion, early drain removal has no proved clinical benefit in these settings besides reduction of hospital stays.  There is sparse literature available with which to make evidence-based guidelines. A standardized guideline for reporting drain use is crucial to providing a better understanding of complications in breast reconstruction related to surgical drains.  There is limited quality evidence that insertion of a drain following axillary lymphadenectomy reduced the odds of developing a seroma and reduced the number of post‐operative seroma aspirations. These benefits should be balanced against an increased length of hospital stay in the drained population. |
| **83** | What is the best type of physiotherapy to help patients regain shoulder mobility following breast cancer surgery; how soon following surgery should it start and what is the best way of delivering treatment? | Khan KA, Mazuquin B, Canaway A, Petrou S, Bruce J. Systematic review of economic evaluations of exercise and physiotherapy for patients treated for breast cancer. Breast Cancer Res Treat. 2019 Jul;176(1):37-52. doi: 10.1007/s10549-019-05235-7. Epub 2019 Apr 17. PMID: 30997624; PMCID: PMC6548756. | **This is an uncertainty** |
| ***F Follow up, detection of recurrence and management of long-term surgical complications*** | | |  |
| **84** | What is the best way of providing practical and psychological support and follow up care for patients after breast cancer surgery; for how long should this be offered and should follow up be stratified according to patient or cancer-related factors? | Matthews H, Grunfeld EA, Turner A. The efficacy of interventions to improve psychosocial outcomes following surgical treatment for breast cancer: a systematic review and meta-analysis. Psychooncology. 2017 May;26(5):593-607. doi: 10.1002/pon.4199. Epub 2016 Aug 2. PMID: 27333194.  Joshi A, Larkins S, Evans R, Moodley N, Brown A, Sabesan S. Use and impact of breast cancer survivorship care plans: a systematic review. Breast Cancer. 2021 Nov;28(6):1292-1317. doi: 10.1007/s12282-021-01267-4. Epub 2021 Jun 19. PMID: 34146242.  Kapoor A, Nambisan P, Baker E. Mobile applications for breast cancer survivorship and self-management: A systematic review. Health Informatics J. 2020 Dec;26(4):2892-2905. Doi: 10.1177/1460458220950853. Epub 2020 Aug 25. PMID: 32842830.  Kyriazoglou A, Zagouri F, Fotiou D, Dimitrakakis C, Marinopoulos S, Zakopoulou R, Kaparelou M, Zygogianni A, Dimopoulos MA. Discrepancies of current recommendations in breast cancer follow-up: a systematic review. Breast Cancer. 2019 Sep;26(5):681-686. Doi: 10.1007/s12282-019-00963-6. Epub 2019 Mar 18. PMID: 30887287.  Cifu G, Power MC, Shomstein S, Arem H. Mindfulness-based interventions and cognitive function among breast cancer survivors: a systematic review. BMC Cancer. 2018 Nov 26;18(1):1163. doi: 10.1186/s12885-018-5065-3. PMID: 30477450; PMCID: PMC6260900.  Lafranconi A, Pylkkänen L, Deandrea S, Bramesfeld A, Lerda D, Neamțiu L, Saz-Parkinson Z, Posso M, Rigau D, Sola I, Alonso-Coello P, Martinez-Zapata MJ. Intensive follow-up for women with breast cancer: review of clinical, economic and patient's preference domains through evidence to decision framework. Health Qual Life Outcomes. 2017 Oct 19;15(1):206. doi: 10.1186/s12955-017-0779-5. PMID: 29052503; PMCID: PMC5649085.  Browall M, Forsberg C, Wengström Y. Assessing patient outcomes and cost-effectiveness of nurse-led follow-up for women with breast cancer - have relevant and sensitive evaluation measures been used? J Clin Nurs. 2017 Jul;26(13-14):1770-1786. doi: 10.1111/jocn.13496. Epub 2017 Mar 20. PMID: 27487478.  Roberts KE, Rickett K, Feng S, Vagenas D, Woodward NE. Exercise therapies for preventing or treating aromatase inhibitor‐induced musculoskeletal symptoms in early breast cancer. Cochrane Database of Systematic Reviews 2020, Issue 1. Art. No.: CD012988. DOI: 10.1002/14651858.CD012988.pub2. Accessed 22 December 2021.  Turner RR, Steed L, Quirk H, Greasley RU, Saxton JM, Taylor SJC, Rosario DJ, Thaha MA, Bourke L. Interventions for promoting habitual exercise in people living with and beyond cancer. Cochrane Database of Systematic Reviews 2018, Issue 9. Art. No.: CD010192. DOI: 10.1002/14651858.CD010192.pub3. Accessed 22 December 2021.  Li J, Liu Y, Jiang J, Peng X, Hu X. Effect of telehealth interventions on quality of life in cancer survivors: A systematic review and meta-analysis of randomized controlled trials. Int J Nurs Stud. 2021 Oct;122:103970. doi: 10.1016/j.ijnurstu.2021.103970. Epub 2021 May 4. PMID: 34303269.  Joshi A, Larkins S, Evans R, Moodley N, Brown A, Sabesan S. Use and impact of breast cancer survivorship care plans: a systematic review. Breast Cancer. 2021 Nov;28(6):1292-1317. doi: 10.1007/s12282-021-01267-4. Epub 2021 Jun 19. PMID: 34146242. | **Merge with Q2:** What is the impact of a breast cancer diagnosis on patient’s wellbeing, how much information and psychological support do patients want around the time of diagnosis, and what are the best methods to provide and individualise information and support/counselling in the short and longer term?  MBSR may improve quality of life slightly at the end of the intervention but may result in little to no difference later on.  No clear evidence of benefit, but likely underpowered findings. Need further understanding of the mechanism of AIMSS, a single clear definition of the condition, and phase III RCTs adequately powered to test targeted exercise interventions on the key clinical outcomes in this condition.  Limited high quality evidence or long term follow up  Short-term telehealth intervention was the most effective, but more large, well-designed RCTs are needed to confirm the effects of telehealth interventions on quality of life in cancer survivors.  Existing research provides positive impact of SCPs on more proximal outcomes of patient experience and care delivery but mixed results for health outcomes in breast cancer survivors. Future research should focus on better defining SCP content and ensuring follow-up recommendations are acted upon, and provider feedback is included and use of novel tools to empower stakeholders. |
| **85** | What are the benefits and disadvantages of remote or self-supported follow up vs face-to-face appointments for patients with breast cancer? | Cheng Kkin Fong, Lim Y, Koh Z, Tam W. Home-based multidimensional survivorship programmes for breast cancer survivors. Cochrane Database of Systematic Reviews 2017, Issue 8. Art. No.: CD011152. DOI: 10.1002/14651858.CD011152.pub2  Brown T, Cruickshank S, Noblet M. Specialist breast care nurses for support of women with breast cancer. Cochrane Database of Systematic Reviews 2021, Issue 2. Art. No.: CD005634. DOI: 10.1002/14651858.CD005634.pub3. Accessed 22 December 2021 | **Merge with Q2 above as this is about the best way to provide support**  HBMS programmes appear to have a short‐term beneficial effect of improving breast cancer‐specific quality of life and global quality of life as measured by FACT‐B and EORTC‐C30, respectively. HBMS programmes are also associated with a reduction in anxiety, fatigue and insomnia immediately after the intervention.  SBCN‐led telephone follow‐up interventions were equally as effective as standard care, for women with primary breast cancer. |
| **86** | What is the best method of surveillance to detect breast cancer recurrence and how does this vary according to patient (e.g. age, breast density) or breast cancer related factors? | Haas CB, Nekhlyudov L, Lee JM, Javid SH, Bush M, Johnson D, Gleason T, Kaufman C, Specht J, Stitham S, Wernli KJ. Surveillance for second breast cancer events in women with a personal history of breast cancer using breast MRI: a systematic review and meta-analysis. Breast Cancer Res Treat. 2020  Swinnen J, Keupers M, Soens J, Lavens M, Postema S, Van Ongeval C. Breast imaging surveillance after curative treatment for primary non-metastasised breast cancer in non-high-risk women: a systematic review. Insights Imaging. 2018 Dec;9(6):961-970. Doi: 10.1007/s13244-018-0667-5. Epub 2018 Nov 8. PMID: 30411278; PMCID: PMC6269345.  Muradali D, Kennedy EB, Eisen A, Holloway CMB, Smith CR, Chiarelli AM. Breast screening for survivors of breast cancer: A systematic review. Prev Med. 2017 Oct;103:70-75. Doi: 10.1016/j.ypmed.2017.07.026. Epub 2017 Jul 29. PMID: 28765083. | Annual mammography is currently the 'gold standard' for breast imaging surveillance. The role of digital breast tomosynthesis (DBT) remains to be further investigated. Most guidelines do not recommend routine breast ultrasound or MRI surveillance, unless indicated by additional risk factors.  **This is an evidence uncertainty** |
| **87** | What factors increase the risk of breast cancer recurrence after surgery and is it possible to predict what patients are at higher risk to help them make a more informed decision about breast cancer surgery? | Infection and post-operative complications  Savioli F, Edwards J, McMillan D, Stallard S, Doughty J, Romics L. The effect of postoperative complications on survival and recurrence after surgery for breast cancer: A systematic review and meta-analysis. Crit Rev Oncol Hematol. 2020 Nov;155:103075. Doi: 10.1016/j.critrevonc.2020.103075. Epub 2020 Aug 18. PMID: 32987333.  O’Connor RÍ, Kiely PA, Dunne CP. The relationship between post-surgery infection and breast cancer recurrence. J Hosp Infect. 2020 Nov;106(3):522-535. Doi: 10.1016/j.jhin.2020.08.004. Epub 2020 Aug 13. PMID: 32800825.  Atakpa EC, Thorat MA, Cuzick J, Brentnall AR. Mammographic density, endocrine therapy and breast cancer risk: a prognostic and predictive biomarker review. Cochrane Database Syst Rev. 2021 Oct 26;10(10):CD013091. doi: 10.1002/14651858.CD013091.pub2. PMID: 34697802; PMCID: PMC8545623. | There is low‐/very low‐certainty evidence to support the hypothesis that breast density change following endocrine therapy is a prognostic biomarker for treatment or prevention and even less evidence that it is a predictive marker.  Further research is warranted to assess mammographic density as a biomarker for all classes of endocrine therapy and review endpoints.  **This is an evidence uncertainty** |
| **88** | What are the best surgical options for patients who get locoregional recurrence of their breast cancer following surgery? | Walstra CJEF, Schipper RJ, Poodt IGM, van Riet YE, Voogd AC, van der Sangen MJC, Nieuwenhuijzen GAP. Repeat breast-conserving therapy for ipsilateral breast cancer recurrence: A systematic review. Eur J Surg Oncol. 2019 Aug;45(8):1317-1327. Doi: 10.1016/j.ejso.2019.02.008. Epub 2019 Feb 10. PMID: 30795956.  Mo C, Ruan W, Lin J, Chen H, Chen X. Repeat Breast-Conserving Surgery Versus Salvage Mastectomy for Ipsilateral Breast Tumour Recurrence After Breast-Conserving Surgery in Breast Cancer Patients: A Meta-Analysis. Front Oncol. 2021 Nov 23;11:734719. Doi: 10.3389/fonc.2021.734719. PMID: 34888233; PMCID: PMC8650120. | More evidence needed  **This is an evidence uncertainty** |
| **89** | What is the risk of long term complications following breast surgery and how is this impacted by obesity and other patient factors? |  | **Merge with Q80** on complications  How can we minimise short and long-term complications and scarring following breast cancer surgery? |
| **90** | What causes cording following breast cancer surgery, how many women get it and how can it best be prevented or treated? | No literature identified | **Merge with Q80** on complications  How can we minimise short and long-term complications and scarring following breast cancer surgery? |
| **91** | How many patients develop lymphoedema following breast cancer surgery, what impact does it have and how should it best be managed, including the indications for and outcomes of surgery to treat lymphoedema? (re-worded to include Q91 on surgery) | Management  ***Conservative management including manual lymphatic drainage and exercise therapy***  Stuiver MM, ten Tusscher MR, Agasi-Idenburg CS, Lucas C, Aaronson NK, Bossuyt PMM. Conservative interventions for preventing clinically detectable upper-limb lymphoedema in patients who are at risk of developing lymphoedema after breast cancer therapy. Cochrane Database of Systematic Reviews 2015, Issue 2. Art. No.: CD009765. DOI: 10.1002/14651858.CD009765.pub2  Ezzo J, Manheimer E, McNeely ML, Howell DM, Weiss R, Johansson KI, Bao T, Bily L, Tuppo CM, Williams AF, Karadibak D. Manual lymphatic drainage for lymphedema following breast cancer treatment. Cochrane Database Syst Rev. 2015 May 21;(5):CD003475. Doi: 10.1002/14651858.CD003475.pub2. PMID: 25994425; PMCID: PMC4966288.  Liang M, Chen Q, Peng K, Deng L, He L, Hou Y, Zhang Y, Guo J, Mei Z, Li L. Manual lymphatic drainage for lymphedema in patients after breast cancer surgery: A systematic review and meta-analysis of randomized controlled trials. Medicine (Baltimore). 2020 Dec 4;99(49):e23192. Doi: 10.1097/MD.0000000000023192. PMID: 33285693; PMCID: PMC7717855. (no effect of MLD)  Thompson B, Gaitatzis K, Janse de Jonge X, Blackwell R, Koelmeyer LA. Manual lymphatic drainage treatment for lymphedema: a systematic review of the literature. J Cancer Surviv. 2021 Apr;15(2):244-258. Doi: 10.1007/s11764-020-00928-1. Epub 2020 Aug 15. PMID: 32803533 (more research needed)  Rangon FB, da Silva J, Dibai-Filho AV, Guirro RRJ, Guirro ECO. Effects of Complex Physical Therapy and Multimodal Approaches on Lymphedema Secondary to Breast Cancer: A Systematic Review and Meta-analysis of Randomized Controlled Trials. Arch Phys Med Rehabil. 2021 Aug 15:S0003-9993(21)01358-7. Doi: 10.1016/j.apmr.2021.06.027. Epub ahead of print. PMID: 34407446.  Hasenoehrl T, Palma S, Ramazanova D, Kölbl H, Dorner TE, Keilani M, Crevenna R. Resistance exercise and breast cancer-related lymphedema-a systematic review update and meta-analysis. Support Care Cancer. 2020 Aug;28(8):3593-3603. Doi: 10.1007/s00520-020-05521-x. Epub 2020 May 15. PMID: 32415386; PMCID: PMC7316683.  Hörmann J, Vach W, Jakob M, Seghers S, Saxer F. Kinesiotaping for postoperative oedema – what is the evidence? A systematic review. BMC Sports Sci Med Rehabil. 2020 Mar 2;12:14. Doi: 10.1186/s13102-020-00162-3. PMID: 32158546; PMCID: PMC7052984.  Saraswathi V, Latha S, Niraimathi K, Vidhubala E. Managing Lymphedema, Increasing Range of Motion, and Quality of Life through Yoga Therapy among Breast Cancer Survivors: A Systematic Review. Int J Yoga. 2021 Jan-Apr;14(1):3-17. Doi: 10.4103/ijoy.IJOY_73_19. Epub 2021 Feb 5. PMID: 33840972; PMCID: PMC8023442.  Shah C, Zambelli-Weiner A, Delgado N, Sier A, Bauserman R, Nelms J. The impact of monitoring techniques on progression to chronic breast cancer-related lymphedema: a meta-analysis comparing bioimpedance spectroscopy versus circumferential measurements. Breast Cancer Res Treat. 2021 Feb;185(3):709-740. Doi: 10.1007/s10549-020-05988-6. Epub 2020 Nov 27. PMID: 33245458; PMCID: PMC7921068.  ***Alternative and complementary therapies***  Gao Y, Ma T, Han M, Yu M, Wang X, Lv Y, Wang X. Effects of Acupuncture and Moxibustion on Breast Cancer-Related Lymphedema: A Systematic Review and Meta-Analysis of Randomized Controlled Trials. Integr Cancer Ther. 2021 Jan-Dec;20:15347354211044107. Doi: 10.1177/15347354211044107. PMID: 34521235; PMCID: PMC8447094.  Jin H, Xiang Y, Feng Y, Zhang Y, Liu S, Ruan S, Zhou H. Effectiveness and Safety of Acupuncture Moxibustion Therapy Used in Breast Cancer-Related Lymphedema: A Systematic Review and Meta-Analysis. Evid Based Complement Alternat Med. 2020 May 11;2020:3237451. Doi: 10.1155/2020/3237451. PMID: 32454855; PMCID: PMC7240793  Yu S, Zhu L, Xie P, Jiang S, Yang Z, He J, Ren Y. Effects of acupuncture on breast cancer-related lymphoedema: A systematic review and meta-analysis. Explore (NY). 2020 Mar-Apr;16(2):97-102. doi: 10.1016/j.explore.2019.06.002. Epub 2019 Jun 26. PMID: 31303328. | Lots of reviews but more high quality research is needed  **This is an evidence uncertainty**  Merge all lymphoedema questions (91, 92)  AC may be an effective method for improving the condition of breast cancer-related lymphoedema. However, due to the high risk of bias and the low quality of the available studies, further high-quality RCTs are needed to confirm the efficacy of AC for breast cancer-related lymphoedema patients. |
| **92** | What is the role and outcomes of surgery to treat lymphoedema? | ***Surgical interventions***  Markkula SP, Leung N, Allen VB, Furniss D. Surgical interventions for the prevention or treatment of lymphoedema after breast cancer treatment. Cochrane Database of Systematic Reviews 2019, Issue 2. Art. No.: CD011433. DOI: 10.1002/14651858.CD011433.pub2  Wang Y, Ge Y, Xing W, Liu J, Wu J, Lin H, Lu Y. The effectiveness and safety of low-level laser therapy on breast cancer-related lymphedema: An overview and update of systematic reviews. Lasers Med Sci. 2021 Nov 15. Doi: 10.1007/s10103-021-03446-3. Epub ahead of print. PMID: 34779937.  Sekigami Y, Char S, Mullen C, Huber K, Cao Y, Buchsbaum R, Graham R, Nardello S, Singhal D, Chatterjee A. Cost-Effectiveness Analysis: Lymph Node Transfer vs Lymphovenous Bypass for Breast Cancer-Related Lymphedema. J Am Coll Surg. 2021 Jun;232(6):837-845. Doi: 10.1016/j.jamcollsurg.2021.02.013. Epub 2021 Mar 5. PMID: 33684564.  ABS Guidance: 2017  Comments: ABS recommends that the technique of liposuction for lymphoedema should be performed within a research programme or as part of an audited clinical practice. Practitioners should be experienced at liposuction and be able to obtain comprehensive informed consent from patients | **Merge with Q91 (reworded)** How many patients develop lymphoedema following breast cancer surgery, what impact does it have and how should it best be managed, including the indications for and outcomes of surgery to treat lymphoedema? |
| **93** | What factors predict for chronic (long-term) pain following breast cancer surgery and how can this be prevented or best managed if it does develop? | Moloney NA, Pocovi NC, Dylke ES, Graham PL, De Groef A. Psychological Factors Are Associated with Pain at All Time Frames After Breast Cancer Surgery: A Systematic Review with Meta-Analyses. Pain Med. 2021 Apr 20;22(4):915-947. Doi: 10.1093/pm/pnaa363. PMID: 33547465.  Wang L, Cohen JC, Devasenapathy N, Hong BY, Kheyson S, Lu D, Oparin Y, Kennedy SA, Romerosa B, Arora N, Kwon HY, Jackson K, Prasad M, Jayasekera D, Li A, Guarna G, Natalwalla S, Couban RJ, Reid S, Khan JS, McGillion M, Busse JW. Prevalence and intensity of persistent post-surgical pain following breast cancer surgery: a systematic review and meta-analysis of observational studies. Br J Anaesth. 2020 Sep;125(3):346-357. Doi: 10.1016/j.bja.2020.04.088. Epub 2020 Jun 28. PMID: 32611524.  McCowat M, Fleming L, Vibholm J, Dixon D. The Psychological Predictors of Acute and Chronic Pain in Women Following Breast Cancer Surgery: A Systematic Review. Clin J Pain. 2019 Mar;35(3):261-271. Doi: 10.1097/AJP.0000000000000672. PMID: 30531400.  Leysen L, Beckwée D, Nijs J, Pas R, Bilterys T, Vermeir S, Adriaenssens N. Risk factors of pain in breast cancer survivors: a systematic review and meta-analysis. Support Care Cancer. 2017 Dec;25(12):3607-3643. Doi: 10.1007/s00520-017-3824-3. Epub 2017 Aug 10. PMID: 28799015.  Rai AS, Khan JS, Dhaliwal J, Busse JW, Choi S, Devereaux PJ, Clarke H. Preoperative pregabalin or gabapentin for acute and chronic postoperative pain among patients undergoing breast cancer surgery: A systematic review and meta-analysis of randomized controlled trials. J Plast Reconstr Aesthet Surg. 2017 Oct;70(10):1317-1328. Doi: 10.1016/j.bjps.2017.05.054. Epub 2017 Jun 9. PMID: 28751024.  Johannsen M, Frederiksen Y, Jensen AB, Zachariae R. Psychosocial predictors of posttreatment pain after nonmetastatic breast cancer treatment: a systematic review and meta-analysis of prospective studies. J Pain Res. 2017 Dec 21;11:23-36. doi: 10.2147/JPR.S124665. PMID: 29317846; PMCID: PMC5743183.  Pinheiro da Silva F, Moreira GM, Zomkowski K, Amaral de Noronha M, Flores Sperandio F. Manual Therapy as Treatment for Chronic Musculoskeletal Pain in Female Breast Cancer Survivors: A Systematic Review and Meta-Analysis. J Manipulative Physiol Ther. 2019 Sep;42(7):503-513. Doi: 10.1016/j.jmpt.2018.12.007. PMID: 31864435. | **Merge with Q77** about acute pain to have one pain-related question (reworded)  How can we best prepare women for pain following breast surgery; manage this pain and prevent chronic pain and best manage this if it develops? |
| **94** | Could 3D scanning be used to improve prostheses for women having breast cancer surgery? | No literature found | **This is an evidence uncertainty** |
| ***G Questions about patients at high-risk of developing breast cancer*** | | | |
| **95** | Can we develop better ways of identifying in clinic, women who are at high risk of developing breast cancer? | NICE guidelines (CG164) Familial breast cancer: classification, care and managing breast cancer and related risks in people with a family history of breast cancer  First published 2013, surveillance review of evidence 2018, minor update 20/11/19  NICE guidance (updated 2019)  Hilgart JS, Coles B, Iredale R. Cancer genetic risk assessment for individuals at risk of familial breast cancer. Cochrane Database of Systematic Reviews 2012, Issue 2. Art. No.: CD003721. DOI: 10.1002/14651858.CD003721.pub3. Accessed 14 January 2022.  No EBCTCG or PROSPERO reviews identified. | Recommends use of family history plus carrier probability calculation tools such as BOADICEA or the Manchester scoring system. Further research is recommended into developing and validating models for calculating carrier probability (NICE research recommendations 2019)  2012 BUT Recommended that further research is needed assessing the best means of delivering cancer risk assessment. |
| **96** | What type of imaging should women at high risk of developing breast cancer have; when should it start and how frequently should it be performed? | NICE guidelines (CG164) Familial breast cancer: classification, care and managing breast cancer and related risks in people with a family history of breast cancer  First published 2013, surveillance review of evidence 2018, minor update 20/11/19  FOR WOMEN HIGH RISK BUT NO PERSONAL HX  - Diagnostic outcomes 1x SR (2008) (moderate quality)  - Clinical outcomes 1x SR (2010) on how low dose radiation affects breast cancer risk  FOR WOMEN HIGH RISK AND PERSONAL HX  - 1x SR (2011) on surveillance imaging – moderate evidence on diagnostic outcomes  - Nil on clinical outcomes inc QoL  Forbes C, Fayter D, de Kock S, Quek RG. A systematic review of international guidelines and recommendations for the genetic screening, diagnosis, genetic counseling, and treatment of *BRCA*-mutated breast cancer. Cancer Manag Res. 2019 Mar 22;11:2321-2337. doi: 10.2147/CMAR.S189627. PMID: 30962720; PMCID: PMC6434912. | NICE guidelines, but evidence is old and incomplete further research needed  NB Regional and organizational guidelines differ for genetic screening, counselling, and treatment of patients with *BRCA*-mutated BC. Guideline harmonization would optimize identification and management of these patients. |
| **97** | How can we help women at risk of breast cancer make an informed choice about the risks and benefits of breast cancer screening options? | NICE guidelines (CG164) Familial breast cancer: classification, care and managing breast cancer and related risks in people with a family history of breast cancer  First published 2013, surveillance review of evidence 2018, minor update 20/11/19  NICE guidelines (2019) recommend offering support (risk counselling, psychological counselling and risk management advice). | Recommend offering support (risk counselling, psychological counselling and risk management advice) but based on limited evidence and a literature review of studies which have assessed the process of risk communication for familial cancer has concluded that there is no clear evidence on how to effectively communicate cancer risk information and to ensure that risk estimates are understood. |
| **98** | Are there alternatives to imaging in high risk women not having imaging due to pregnancy or breast feeding? | No evidence in NICE guidelines. No data in Cochrane reviews or EBCTCG, | **This is an unanswered question** |
| **99** | How can information on breast density and its implications for breast cancer risk and diagnosis best be made available and discussed with patients? | No evidence in NICE guidelines. No data in Cochrane or EBCTCG.  Huang S, Houssami N, Brennan M, Nickel B. The impact of mandatory mammographic breast density notification on supplemental screening practice in the United States: a systematic review. Breast Cancer Res Treat. 2021 May;187(1):11-30. Doi: 10.1007/s10549-021-06203-w. Epub 2021 Mar 28. PMID: 33774734. | BDN leads to increased use of supplemental screening. This has implications for women and the health system. These findings can help inform current and future screening programs, where breast density notification is currently implemented or being considered.  **This is an unanswered question** |
| **100** | Would risk stratification of screening using breast density and family history, and/or the use of additional screening tests, allow new and recurrent breast cancers to be detected at an earlier stage and would this be cost-effective? | No recommendations on stratified breast screening in NICE guidelines. No evidence in Cochrane/EBCTCG.  This is the subject of a number of ongoing trials. | **This is (currently) an unanswered question.** |
| **101** | Are there additional screening tests that can be offered to women with dense breasts to reduce the number of later stage diagnoses and/or help earlier detection of recurrence? |  | **Merged with Q100** |
| **102** | What are the options for risk reduction and management of women at high risk of developing breast cancer and what is the impact on quality of life? | **CHEMO-PREVENTION**  Mocellin S, Goodwin A, Pasquali S. Risk‐reducing medications for primary breast cancer: a network meta‐analysis. Cochrane Database of Systematic Reviews 2019, Issue 4. Art. No.: CD012191. DOI: 10.1002/14651858.CD012191.pub2. Accessed 22 December 2021.  **BSO**  Eleje GU, Eke AC, Ezebialu IU, Ikechebelu JI, Ugwu EO, Okonkwo OO. Risk‐reducing bilateral salpingo‐oophorectomy in women with BRCA1 or BRCA2 mutations. Cochrane Database of Systematic Reviews 2018, Issue 8. Art. No.: CD012464. DOI: 10.1002/14651858.CD012464.pub2. Accessed 22 December 2021.  **RRM**  Honold F, Camus M. Prophylactic mastectomy versus surveillance for the prevention of breast cancer in women's BRCA carriers. Medwave. 2018 Jul 9;18(4):e7161. Spanish, English. doi: 10.5867/medwave.2018.04.7160. PMID: 30052622.  Li X, You R, Wang X et al. (2016) Effectiveness of Prophylactic Surgeries in BRCA1 or BRCA2 Mutation Carriers: A Meta-analysis and Systematic Review. Clinical cancer research : an official journal of the American Association for Cancer Research 22:3971-3981.  Razdan SN, Patel V, Jewell S et al. (2016) Quality of life among patients after bilateral prophylactic mastectomy: a systematic review of patient-reported outcomes. Quality of life research : an international journal of quality of life aspects of treatment, care and rehabilitation 25:1409-1421.  Carbine NE, Lostumbo L, Wallace J, Ko H. Risk‐reducing mastectomy for the prevention of primary breast cancer. Cochrane Database of Systematic Reviews 2018, Issue 4. Art. No.: CD002748. DOI: 10.1002/14651858.CD002748.pub4. Accessed 23 December 2021.  **ALL**  NICE guidelines (CG164) Familial breast cancer: classification, care and managing breast cancer and related risks in people with a family history of breast cancer  First published 2013, surveillance review of evidence 2018, minor update 20/11/19 | **Options for Risk reduction answered**  **Impact on quality of life not answered**  AIs appear to be more effective than SERMs (tamoxifen) in reducing the risk of developing breast cancer. AIs are not associated with an increased risk of endometrial cancer and thromboembolic events. However, long‐term data on toxicities from tamoxifen are available while the follow‐up toxicity data on unaffected women taking AIs is relatively short. Additional data from direct comparisons are needed to fully address the issues of breast cancer prevention by risk‐reducing medications, with special regards to acceptability (i.e. the benefit/harm ratio).  There is very low‐certainty evidence that RRSO may increase overall survival and lower HGSC and breast cancer mortality for BRCA1 and BRCA2 carriers. Need further research  Strong evidence that reduces incidence of breast cancer and mortality,  Moderate evidence of association with adverse physical outcomes  Low quality evidence regarding impact of QoL  N=15 studies. bilateral prophylactic mastectomy (BPM) associated with decreased breast cancer risk in BRCA mutation carriers. Contralateral prophylactic mastectomy (CPM) was found to significantly decrease contralateral breast cancer incidence in BRCA mutation carriers. All-cause mortality was found to be significantly lower for patients who underwent CPM. The association between all-cause mortality and BPM was not significant.  N=15 studies. post-BPM, patients were satisfied with the outcomes and report high psychosocial wellbeing and positive body image. The authors report that sexual well-being and somatosensory function are the most negatively affected.  All observational studies – suggest does reduce incidence and mortality but lots of confounding variables and should only be offered to high risk women  Most evidence is pre 2013, with some areas updated to 2017 |
| **103** | In who and when should risk-reducing medication such as anti-oestrogen treatment (e.g. Tamoxifen) be used to reduce breast cancer risk, and what is the best way to communicate and support effective use? | NICE guidelines (CG164) Familial breast cancer: classification, care and managing breast cancer and related risks in people with a family history of breast cancer  First published 2013, surveillance review of evidence 2018, minor update 20/11/19  Mocellin S, Goodwin A, Pasquali S. Risk-reducing medications for primary breast cancer: a network meta-analysis. Cochrane Database Syst Rev. 2019 Apr 29;4(4):CD012191. doi: 10.1002/14651858.CD012191.pub2. PMID: 31032883; PMCID: PMC6487387.  Nelson HD, Fu R, Zakher B, Pappas M, McDonagh M. Medication Use for the Risk Reduction of Primary Breast Cancer in Women: Updated Evidence Report and Systematic Review for the US Preventive Services Task Force. JAMA. 2019 Sep 3;322(9):868-886. Doi: 10.1001/jama.2019.5780. PMID: 31479143. | Has clear guidance on recommendations for chemoprevention and has produced patient decision aids for use in this setting. However, it’s not clear what the ***best*** way to communicate and support effective use is  However NICE also has a research recommendation: “What is the clinical and cost-effectiveness of aromatase inhibitors compared with tamoxifen and raloxifene for reducing the incidence of breast cancer in women with a family history of breast or ovarian cancer” and an RCT is recommended.  For women with an above‐average risk of developing breast cancer, CPAs can reduce the incidence of this disease. AIs appear to be more effective than SERMs (tamoxifen) in reducing the risk of developing breast cancer. AIs are not associated with an increased risk of endometrial cancer and thromboembolic events. However, long‐term data on toxicities from tamoxifen are available while the follow‐up toxicity data on unaffected women taking AIs is relatively short. Additional data from direct comparisons are needed to fully address the issues of breast cancer prevention by risk‐reducing medications, with special regards to acceptability (i.e. the benefit/harm ratio).  Limitations. First, potential publication bias as well as biases of the literature review process, such as including only English-language articles. Second, studies of risk assessment methods varied by size, study populations, reference groups, and methods. Third, RCTs were limited by clinical heterogeneity related to different eligibility criteria, exposure durations and follow-up, adherence, and ascertainment of outcomes. The trials were not designed for subgroup analysis and may have been underpowered to demonstrate treatment effects. Furthermore, no trials directly compared the effects of timing and duration of medication use. Fourth, research is lacking for optimal doses, duration of use, persistence of effects after treatment for most medications, and outcomes in women who are nonwhite, premenopausal, have comorbidities, or are taking additional medications for other indications.  Conclusions  Tamoxifen, raloxifene, and aromatase inhibitors were associated with lower risk of primary invasive breast cancer in women but also were associated with adverse effects that differed between medications. Risk stratification methods to identify patients with increased breast cancer risk demonstrated low accuracy. |
| **104** | What are the risks and benefits risk-reducing medication such as anti-oestrogen therapy (e.g. Tamoxifen) to reduce breast cancer risk and how should this be communicated to patients? |  | **Merge with reworded Q103**  In who and when should risk-reducing medication such as anti-oestrogen treatment (e.g. Tamoxifen) be used to reduce breast cancer risk, and what is the best way to communicate and support effective use |
| **105** | How can we best identify and target the use of endocrine therapy to reduce the risk of breast cancer in appropriate groups? |  | **Merge with reworded Q103**  In who and when should risk-reducing medication such as anti-oestrogen treatment (e.g. Tamoxifen) be used to reduce breast cancer risk, and what is the best way to communicate and support effective use |
| **106** | What is the best clinical pathway and mechanism for support of women at high-risk of breast cancer to make decisions about surgery and then after surgery has been performed? | NICE guidelines (CG164) Familial breast cancer: classification, care and managing breast cancer and related risks in people with a family history of breast cancer  First published 2013, surveillance review of evidence 2018, minor update 20/11/19  Carbine NE, Lostumbo L, Wallace J, Ko H. Risk‐reducing mastectomy for the prevention of primary breast cancer. Cochrane Database of Systematic Reviews 2018, Issue 4. Art. No.: CD002748. DOI: 10.1002/14651858.CD002748.pub4. Accessed 23 December 2021.  Ongoing review in PROSPERO on interventions assessing psychological adjustment in BRCA1/2 mutation carriers following genetic testing.  No EBCTCG data.  Jeffers L, Reid J, Fitzsimons D, Morrison PJ, Dempster M. Interventions to improve psychosocial well-being in female BRCA-mutation carriers following risk-reducing surgery. Cochrane Database of Systematic Reviews 2019, Issue 10. Art. No.: CD012894. DOI: 10.1002/14651858.CD012894.pub2 | NICE FH guidelines describe counselling and support pathways for women at high-risk although details of ***best*** pathway are unclear  NICE research recommendation – “further research is recommended to compare psychosocial and clinical outcomes in women who choose and women who do not choose to have risk-reducing surgery  bilateral RRM resulted in diminished satisfaction with body image and sexual feelings but no discussion of post-operation support. All observational studies  The effect of psychosocial interventions on quality of life and emotional well‐being in female BRCA carriers who undergo risk‐reducing surgery is uncertain given the very low methodological quality in the two studies included in the review.  **This is an unanswered question** |
| **107** | What are the risks and benefits of mastectomy with or without breast reconstruction in women at high risk of breast cancer, and when and/or at what age should surgery be performed? | **RRM**  Honold F, Camus M. Prophylactic mastectomy versus surveillance for the prevention of breast cancer in women's BRCA carriers. Medwave. 2018 Jul 9;18(4):e7161. Spanish, English. doi: 10.5867/medwave.2018.04.7160. PMID: 30052622.  Li X, You R, Wang X et al. (2016) Effectiveness of Prophylactic Surgeries in BRCA1 or BRCA2 Mutation Carriers: A Meta-analysis and Systematic Review. Clinical cancer research : an official journal of the American Association for Cancer Research 22:3971-3981.  Razdan SN, Patel V, Jewell S et al. (2016) Quality of life among patients after bilateral prophylactic mastectomy: a systematic review of patient-reported outcomes. Quality of life research : an international journal of quality of life aspects of treatment, care and rehabilitation 25:1409-1421.  Carbine NE, Lostumbo L, Wallace J, Ko H. Risk‐reducing mastectomy for the prevention of primary breast cancer. Cochrane Database of Systematic Reviews 2018, Issue 4. Art. No.: CD002748. DOI: 10.1002/14651858.CD002748.pub4. Accessed 23 December 2021.  NICE guidelines (CG164) Familial breast cancer: classification, care and managing breast cancer and related risks in people with a family history of breast cancer  First published 2013, surveillance review of evidence 2018, minor update 20/11/19  No data in NICE/Cochrane/EBCTCG/PROSPERO on optimal age for risk-reducing breast surgery  NICE evidence review also states that there is no clear evidence on the optimal surgical technique for risk reducing mastectomy. | **Good evidence about incidence/mortality but less evidence of QoL or age of surgery**  Strong evidence that reduces incidence of breast cancer and mortality,  Moderate evidence of association with adverse physical outcomes  Low quality evidence regarding impact of QoL  N=15 studies. bilateral prophylactic mastectomy (BPM) associated with decreased breast cancer risk in BRCA mutation carriers. Contralateral prophylactic mastectomy (CPM) was found to significantly decrease contralateral breast cancer incidence in BRCA mutation carriers. All-cause mortality was found to be significantly lower for patients who underwent CPM. The association between all-cause mortality and BPM was not significant.  N=15 studies. post-BPM, patients were satisfied with the outcomes and report high psychosocial wellbeing and positive body image. The authors report that sexual well-being and somatosensory function are the most negatively affected.  All observational studies – suggest does reduce incidence and mortality but lots of confounding variables and should only be offered to high risk women. Also reports re-operation rates and notes that RRM resulted in diminished satisfaction with body image and sexual feelings.  Most evidence is pre 2013, with some areas updated to 2017 NICE research recommendation regarding psychosocial and clinical outcomes following RRM. |
| **108** | Which women who do not carry the BRCA gene, are at high risk of breast cancer and would benefit from risk reducing surgery? | NICE guidelines (CG164) Familial breast cancer: classification, care and managing breast cancer and related risks in people with a family history of breast cancer  First published 2013, surveillance review of evidence 2018, minor update 20/11/19  Carbine NE, Lostumbo L, Wallace J, Ko H. Risk‐reducing mastectomy for the prevention of primary breast cancer. Cochrane Database of Systematic Reviews 2018, Issue 4. Art. No.: CD002748. DOI: 10.1002/14651858.CD002748.pub4. Accessed 23 December 2021.  ABS guidance (2017)  No EBCTCG or PROSPERO reviews. | NICE guidelines recommend discussing risks/benefits of RRM with BRCA1/2 and TP53 mutation carriers only.  Suggests that RRM should only be considered in these at high-risk of disease (i.e. BRCA1/2 or equivalent risk).  Consideration of contralateral prophylactic mastectomy only for unilateral breast cancer and does not address this question.  **This is an unanswered question** |
| **109** | Does risk reducing surgery improve survival in women at risk of developing breast cancer? | See Cochrane review (Carbine 2018) and NICE guidelines confirming that RRM reduces the risk of breast cancer in women with a FH or BRCA1/2 mutation carriers. Both confirm that RRM reduces breast cancer mortality. | Answered question  See evidence for 87 & 90 on RRM |
| **110** | How can we provide services to support preventative measures such as weight management, lifestyle advice, and new research outputs and demonstrate that they reduce breast cancer risk? | NICE guidelines (CG164) Familial breast cancer: classification, care and managing breast cancer and related risks in people with a family history of breast cancer  First published 2013, surveillance review of evidence 2018, minor update 20/11/19 | NICE guidance makes recommendations that alcohol, weight and physical activity may affect breast cancer risk. However no evidence was identified to support how to provide services to modify these risk factors nor to demonstrate a resulting reduction in breast cancer risk.  **This is an unanswered question** |
| **111** | How should breast cancer services and high-risk clinics ensure equity of access and engagement with all ethnic and socioeconomic groups? |  | Not research question – service provision and equity of access  EXCLUDE |
| **112** | What is the best way of estimating and communicating future breast cancer risk in patients with genetic risk factors or a previous breast cancer? | NICE guidelines (CG164) Familial breast cancer: classification, care and managing breast cancer and related risks in people with a family history of breast cancer  First published 2013, surveillance review of evidence 2018, minor update 20/11/19  Hilgart JS, Coles B, Iredale R. Cancer genetic risk assessment for individuals at risk of familial breast cancer. Cochrane Database of Systematic Reviews 2012, Issue 2. Art. No.: CD003721. DOI: 10.1002/14651858.CD003721.pub3. Accessed 14 January 2022.  The 2012 Cochrane review (Hilgart et al) recommended that further research is needed assessing the best means of delivering cancer risk assessment  Louro J, Posso M, Hilton Boon M, Román M, Domingo L, Castells X, Sala M. A systematic review and quality assessment of individualised breast cancer risk prediction models. Br J Cancer. 2019 Jul;121(1):76-85. doi: 10.1038/s41416-019-0476-8. Epub 2019 May 22. PMID: 31114019; PMCID: PMC6738106. | NICE evidence review states that evidence related to breast cancer risk communication in women with a family history of breast cancer is limited and relates to mainly qualitative research. The guidance cites a literature review from 1998 stating that there was no clear evidence about how to sensitively and effectively communicate cancer risk information to individuals and families at risk of familial cancer.  2012 BUT recommended that further research is needed assessing the best means of delivering cancer risk assessment  Individualised risk prediction models are promising tools for implementing risk-based screening policies. However, it is a challenge to recommend any of them since they need further improvement in their quality and discriminatory capacity.  **This is an unanswered question** |
