# Supplementary material for: Identifying research priorities in breast cancer surgery: a UK priority setting partnership with the James Lind Alliance
Source: Breast Cancer Res Treat. 2022 Nov 1;197(1):39–49. doi: 10.1007/s10549-022-06756-4 (PMC9628302; doi:10.1007/s10549-022-06756-4)
Supplement: Supplementary file 4 — Supplementary file4 (DOCX 179 KB) [file 10549_2022_6756_MOESM4_ESM.docx]

**APPENDIX 4: Results of Interim Priority Setting Survey by Respondent group**

| **Question** | **Patients and carers** | | | **High risk women** | | | | **Healthcare professionals** | | | |
| --- | --- | --- | --- | --- | --- | --- | --- | --- | --- | --- | --- |
|  | **% PC ranking Q in top 10** | **Patient/Carer rank** | | **% HR ranking Q in top 10** | | **High risk rank** | | **% HCPs ranking Q in top 10** | | **HCP rank** | |
| What is the best way to ensure that patients can make fully informed choices about their breast cancer surgery options, feel involved in the process, and have sufficient time to make their decisions? | 49.1 | 1 | | 26.5 | | 8= | | 15.1 | | 21= | |
| What are the outcomes of mastectomy with and without breast reconstruction; how should these be discussed with patients so that they have realistic expectations of outcomes and can make informed decisions? | 40.4 | 2 | | 23.5 | | 11= | | 7.5 | | 44= | |
| What are the outcomes of mastectomy for symmetry in women with breast cancer but with no additional genetic risk? When should this be performed and how can women’s decision-making be best supported? | 36.5 | 3 | | 14.7 | | 23= | | 12.3 | | 30= | |
| When is symmetrisation (or balancing) surgery required, and should it be performed at the same time as the breast cancer surgery or later? | 36.3 | 4 | | 11.8 | | 27= | | 9.4 | | 39= | |
| How does a breast cancer diagnosis impact on patients’ wellbeing? What information and support do patients want around the time of diagnosis, during and after treatment, and what are the best methods to individualise this? | 34.2 | 5 | | 11.8 | | 27= | | 15.1 | | 21= | |
| What factors increase the risk of breast cancer returning; Is it possible to predict which patients are at higher risk to help them make a more informed decision about breast cancer surgery? | 32.7 | 6 | | 11.8 | | 27= | | 16.0 | | 16= | |
| What are the outcomes of mastectomy after breast cancer and can better surgical techniques be used to improve the appearance of the chest wall? | 31.6 | 7 | | 17.6 | | 17= | | 5.7 | | 50= | |
| What is the best method of follow up imaging to detect whether the cancer has returned following breast cancer surgery and how is this influenced by tumour and patient characteristics (e.g. patient age, hormone receptor status)? | 29.2 | 8 | | 8.8 | | 31= | | 16.0 | | 16= | |
| Can new technologies be used predict whether a breast cancer has been completely removed at the time of surgery to reduce the need for a second operation? | 21.9 | 9 | | 8.8 | | 31= | | 30.2 | | 3 | |
| What is the best type of physiotherapy for patients following breast cancer surgery; when should it start and how should it be delivered? | 21.1 | 10 | | 17.6 | | 17= | | 6.6 | | 46= | |
| What are the risk factors for post-operative seroma (fluid collections) following breast cancer surgery, how can they be prevented? Are drains needed to manage them and if so, when? | 19.9 | 11 | | 14.7 | | 23= | | 16.0 | | 16= | |
| Would risk-based screening based on breast density and family history, and/or the use of additional screening tests, allow new and recurrent breast cancers to be detected at an earlier stage; would this improve cancer outcomes or be cost-effective? | 18.7 | 12= | | 23.5 | | 11= | | 20.8 | | 10= | |
| When should risk-reducing medication such as anti-oestrogen treatment (e.g. Tamoxifen) be used to reduce breast cancer risk and for which women? | 18.7 | 12= | | 17.6 | | 17= | | 11.3 | | 34= | |
| How can the short and long-term complications of breast cancer surgery (e.g. scarring and wound problems) be minimised? | 18.7 | 12= | | 29.4 | | 5= | | 5.7 | | 50= | |
| How many patients develop lymphoedema (swelling of the breast or arm) following breast cancer surgery, how are patients affected by it and how should it best be managed? | 18.1 | 15 | | 5.9 | | 39= | | 21.7 | | 9 | |
| What is the best way of estimating and communicating future breast cancer risk and risk reduction strategies in patients with genetic risk factors and/or a previous breast cancer? | 17.5 | 16 | | 32.4 | | 4 | | 6.6 | | 46= | |
|  | **Patients and carers** | | | **High risk women** | | | | **Healthcare professionals** | | | |
|  | **% PC ranking in top 10** | **Patient/Carer rank** | | **% HR ranking in top 10** | | **High risk rank** | | **% HCPs ranking in top 10** | | **HCP rank** | |
| Can preventative measures, such as lifestyle changes, reduce breast cancer risk and how can this advice be effectively communicated? | 17.0 | 17 | | 29.4 | | 5= | | 17.0 | | 14= | |
| What is the impact of mastectomy with or without breast reconstruction on quality of life for women at high risk of breast cancer, and when and/or at what age should surgery be performed? | 16.7 | 18= | | 47.1 | | 2 | | 5.7 | | 50= | |
| How do the cancer outcomes of breast conserving surgery and radiotherapy compare with those following mastectomy for breast cancer? | 16.7 | 18= | | 8.8 | | 31= | | 13.2 | | 28= | |
| How well do different breast symptoms (e.g. breast pain) predict an eventual breast cancer diagnosis, and what is the best way of investigating different symptoms to identify those who do not have breast cancer and those who do to provide reassurance to patients? | 16.1 | 20 | | 17.6 | | 17= | | 18.9 | | 13 | |
| What is the best management of ductal carcinoma in situ (pre-invasive breast cancer) and how is this influenced by tumour and patient characteristics (e.g. patient age, hormone receptor status)? | 15.8 | 21 | | 5.9 | | 39= | | 27.4 | | 5= | |
| Can complete lymph node removal (axillary clearance) be avoided in patients with spread of cancer to the armpit (axilla); what are the alternatives and the outcomes of this approach? | 15.2 | 22 | | 0.0 | | NR | | 29.2 | | 4 | |
| Are there better ways of identifying people who are at high risk of developing breast cancer? | 14.3 | 23 | | 23.5 | | 11= | | 10.4 | | 37= | |
| How can information on breast density and the implications for future breast cancer risk be made available and discussed with women? | 14.0 | 24 | | 17.6 | | 17= | | 14.2 | | 26= | |
| What type of imaging should people who are at high risk of developing breast cancer have; when should it start and how often should it be performed? | 13.2 | 25 | | 26.5 | | 8= | | 6.6 | | 46= | |
| Do pre-operative preparation, exercise and enhanced recovery programmes reduce time in hospital and improve outcomes for patients having breast cancer surgery? | 12.6 | 26 | | 20.6 | | 15= | | 4.7 | | 54= | |
| What is the best way to prepare patients for pain following breast cancer surgery, manage the pain after surgery and prevent and treat long-term (chronic) pain? | 12.0 | 27 | | 8.8 | | 31= | | 7.5 | | 44= | |
| How can decisions about the order of breast cancer treatments (surgery; chemotherapy and/or radiotherapy) be tailored for individual patients? | 11.4 | 28 | | 11.8 | | 27= | | 16.0 | | 16= | |
| Is it possible to predict survival rates, benefits and the risk of the cancer returning for patients having hormone (endocrine) treatment before breast cancer surgery? | 11.1 | 29 | | 2.9 | | 46= | | 3.8 | | 58 | |
| What are the outcomes (e.g. cancer-related and quality of life) of procedures that combine lumpectomy to remove the breast cancer with plastic surgical techniques to reshape the breast (oncoplastic breast conserving surgery); how do these compare with lumpectomy and mastectomy with and without breast reconstruction; how should this information be discussed with patients to support informed decision-making? | 10.8 | 30 | | 2.9 | | 46= | | 15.1 | | 21= | |
| Is it possible to predict which patients with hormone sensitive breast cancer should have chemotherapy and which should have hormone (endocrine) therapy prior to surgery? | 9.9 | 31= | | 5.9 | | 39= | | 10.4 | | 37= | |
| Which women who do not carry the BRCA gene, are at high risk of breast cancer and would benefit from risk reducing surgery? | 9.9 | 31= | | 14.7 | | 23= | | 16.0 | | 16= | |
|  | **Patients and carers** | | | | **High risk women** | | | | **Healthcare professionals** | | |
|  | **% PC ranking in top 10** | | **Patient/Carer rank** | | **% HR ranking in top 10** | | **High risk rank** | | **% HCPs ranking in top 10** | | **HCP rank** |
| What type of surgery should be performed for patients in whom breast cancer has recurred (come back) in the breast or armpit? | 9.1 | | 33 | | 2.9 | | 46= | | 17.0 | | 14= |
| How can women at high-risk of breast cancer be best supported before, during and after risk reduction surgery? (Breast surgery to reduce the risk of breast cancer) | 8.8 | | 34 | | 61.8 | | 1 | | 4.7 | | 54= |
| Do all patients with breast cancer need investigations (e.g. ultrasound) or surgery (e.g. sentinel lymph node biopsy) to their axilla (armpit) to determine if their breast cancer has spread? | 8.5 | | 35 | | 2.9 | | 46= | | 14.2 | | 26= |
| Can the use of short-course treatments (e.g. hormone therapy for a few weeks) before surgery predict the long-term outcomes of breast cancer and identify patients at high risk of breast cancer returning? | 8.2 | | 36= | | 0.0 | | NR | | 11.3 | | 34= |
| How can the experience and outcomes of breast cancer treatment in men be improved? | 8.2 | | 36= | | 2.9 | | 46= | | 15.1 | | 21= |
| Are minimally invasive, image-guided techniques (e.g. vacuum excision or freezing) to remove or destroy the breast cancer a safe and effective alternative to breast cancer surgery? | 8.2 | | 36= | | 2.9 | | 46= | | 34.0 | | 1= |
| In patients having breast chemotherapy before surgery, what is the best way of monitoring the cancer and is it possible to tell whether the cancer has completely responded to treatment without performing an operation? How long, if at all, after finishing chemotherapy should an operation be performed? | 7.9 | | 39= | | 2.9 | | 46= | | 24.5 | | 7 |
| What impact does radiotherapy have on the short and long-term outcomes of different types of breast reconstruction; when should breast reconstruction be performed if radiotherapy is needed; how can poor outcomes be minimised in this group; how can women be supported to make informed treatment decisions? | 7.9 | | 39= | | 8.8 | | 31= | | 20.8 | | 10= |
| How can people at high risk of breast cancer be helped to make an informed choice about the risks and benefits of breast cancer screening options? | 7.6 | | 41 | | 26.5 | | 8= | | 4.7 | | 54= |
| What is the impact of different risk reduction strategies on quality of life, for women at high risk of developing breast cancer? | 7.0 | | 42 | | 41.2 | | 3 | | 9.4 | | 39= |
| Is it possible to develop new options for breast reconstruction for women not suitable for reconstruction using implants or their own tissues? | 6.7 | | 43= | | 14.7 | | 23= | | 12.3 | | 30= |
| Could 3D scanning be used to improve prostheses for women having breast cancer surgery? | 6.7 | | 43= | | 8.8 | | 31= | | 2.8 | | 59 |
| How does the impact of, and response to, treatments given before breast cancer surgery affect patients’ quality of life and well-being? | 6.4 | | 45 | | 2.9 | | 46= | | 8.5 | | 41= |
| What are the short and long-term outcomes (e.g. cancer-related and quality of life) of breast reconstruction using implants with and without biological and synthetic mesh and how can these be improved? | 5.8 | | 46 | | 17.6 | | 17= | | 12.3 | | 30= |
| Are there some low-risk breast cancers or lesions detected by breast screening that do not need treatment at all and how it possible to work out which ones these are? | 5.6 | | 47= | | 5.9 | | 39= | | 34.0 | | 1= |
| What are the long-term cancer outcomes of skin and nipple-sparing mastectomy for breast cancer? | 5.6 | | 47= | | 29.4 | | 5= | | 12.3 | | 30= |
| What is the best surgical management of rarer types of breast cancer? | 5.0 | | 49 | | 2.9 | | 46= | | 8.5 | | 41= |
| Can mastectomy be safely avoided in patients with more than one breast cancer in different parts of the same breast? | 4.4 | | 50= | | 0.0 | | NR | | 22.6 | | 8 |
|  | **Patients and carers** | | | | **High risk women** | | | | **Healthcare professionals** | | |
|  | **% PC ranking in top 10** | | **Patient/Carer rank** | | **% HR ranking in top 10** | | **High risk rank** | | **% HCPs ranking in top 10** | | **HCP rank** |
| In women having a skin/nipple sparing mastectomy, what is the best way to identify breast tissue so that it can be fully removed while maintaining the blood supply to the skin? | 4.4 | | 50= | | 23.5 | | 11= | | 15.1 | | 21= |
| Is it possible to predict which patients with breast cancer who need a mastectomy (removal of the breast) at diagnosis, will be able to have breast conserving surgery if they have chemotherapy before surgery? | 4.1 | | 52= | | 8.8 | | 31= | | 11.3 | | 34= |
| How soon after radiotherapy for breast cancer should further surgery to improve the appearance of the breast be performed? | 4.1 | | 52= | | 5.9 | | 39= | | 8.5 | | 41= |
| What alternative imaging can women at high risk of breast cancer have during pregnancy and/or breast feeding? | 4.1 | | 52= | | 20.6 | | 15= | | 5.7 | | 50= |
| Are dressings needed following breast cancer surgery and if so, which ones should be used and for how long? | 3.8 | | 55 | | 5.9 | | 39= | | 4.7 | | 54= |
| What is the best way to treat the axillary (armpit) lymph nodes in patients with breast cancer who have had chemotherapy before surgery? | 3.5 | | 56 | | 0.0 | | NR | | 27.4 | | 5= |
| What is the most effective way to mark and localise breast cancers and axillary (armpit) lymph nodes in patients having chemotherapy before breast cancer surgery? | 2.9 | | 57= | | 2.9 | | 46= | | 20.8 | | 10= |
| How long should patients have endocrine (hormone) treatment before breast cancer surgery and when should surgery be performed for these patients? | 2.9 | | 57= | | 5.9 | | 39= | | 13.2 | | 28= |
| Does 3D scanning improve the outcomes of reconstructive breast cancer surgery? | 2.3 | | 59 | | 8.8 | | 31= | | 6.6 | | 46= |

Green – top 10 priority; Yellow – top 11-20 priority; Blue – questions carried forward to final prioritisation workshop

‘=’ indicates same rank
